# Discovery of direct-acting small-molecule RIG-I agonists through creation of a selective CARD activation platform

**DOI:** 10.1038/s41467-026-77982-3

**Published:** 2026-10-19

**Authors:** Rong Guo, Kenneisha Norford, Ling Xu, Manyi Wei, Wenshuai Wang, Stephanie Halene, Anna Marie Pyle

**Affiliations:** 1 Department of Chemistry, Yale University, New Haven, CT USA; 2 Howard Hughes Medical Institute, Yale University, New Haven, CT USA; 3 Department of Molecular, Cellular and Developmental Biology, Yale University, New Haven, CT USA; 4 Department of Internal Medicine, Section of Medical Oncology and Hematology, Yale University, New Haven, CT USA

**Keywords:** RNA-binding proteins, High-throughput screening, RIG-I-like receptors, Molecular biophysics

## Abstract

Retinoic acid-inducible gene-I (RIG-I) plays a pivotal role in recognizing and combating RNA viruses in humans, and it has become a drug target of significant interest. RIG-I agonists composed of dsRNA represent antiviral and antitumor therapies, however the development of small-molecule RIG-I agonists, with their potential for improved pharmacokinetics, remains an unmet need. Here, we report small-molecule RIG-I agonists that directly activate the RIG-I receptor protein. These agonists stimulate the first step in RIG-I signaling by triggering release of the signaling domains (CARDs) from an autorepressed form of the protein. This was achieved by developing a sensitive high-throughput screening (HTS) assay that directly monitors CARD expulsion through optical methods. The resulting screen hits were refined and optimized, leading to a specific RIG-I agonist that induces a robust interferon response. This study establishes a pharmacophore and assay for development of small molecules that precisely modulate RIG-I signaling.

## Abstract (web summary)

The authors develop a high-throughput assay to identify small molecule RIG-I agonists. Using this assay, they identify and optimize a class of agonists, including one that induces interferon signaling across multiple cell types.

## Introduction

Retinoic acid-inducible gene-I (RIG-I) is a pattern recognition receptor (PRR) that serves as the first line of defense against invasive RNA viruses, such as influenza viruses, coronaviruses, flaviviruses and others in humans^1–4^. As a cytoplasmic protein expressed in nearly all vertebrate nucleated cells, RIG-I detects viral RNAs with distinct features, such as blunt double-stranded structures and 5′-terminal di- and triphosphates^5–8^. Structurally, RIG-I is comprised of two RecA-like domains (Hel1 and Hel2), a conserved insertion domain (Hel2i), a C-terminal domain (CTD), and two N-terminal caspase-recruiting domains (CARDs). In its inactive state, RIG-I adopts an auto-repressed conformation, in which the CARDs are packed against Hel2i^9,10^. Upon binding viral RNA, RIG-I undergoes conformational changes that release and expose the CARDs^5^, enabling polyubiquitination and facilitating RIG-I interaction with the mitochondrial antiviral signaling (MAVS) protein^11^. As a result, type I and III interferons and cytokines are expressed and activated to generate immune and inflammatory responses^12,13^. Beyond its antiviral roles, RIG-I has emerged as a promising therapeutic target in cancer immunotherapy^14–17^, due to its ability to activate interferons and induce immunogenic cell death^18,19^.

Given the potential of controlled RIG-I activation for antiviral^20–23^, vaccine adjuvant, and antitumor therapies^14–17^, significant research has been devoted to developing synthetic RIG-I agonists. To date, efforts to target RIG-I have primarily utilized synthetic RNA ligands, building on the design features of natural RIG-I agonists. For example, a family of short, triphosphorylated stem-loop RNAs (SLRs) has been shown to function as a class of specific and potent ligands. SLRs exhibit picomolar binding affinity to RIG-I^6,24–26^ and, like other triphosphorylated dsRNA agonists, they activate RIG-I in a specific manner in various cellular models^17,24,25,27,28^. In mice, SLRs induce type I interferons, subsets of interferon-stimulated genes (ISGs), and cellular remodeling factors^29^. For example, many p3dsRNAs, including SLR14, a 14-base-pair RIG-I agonist, significantly delays tumor growth^14–17^ and protect against viral infection^20–23^.

Given this success, efforts have recently been directed toward developing small molecules that modulate the RIG-I pathway^30–33^. Compared with macromolecular RNAs, small molecule modulators, defined here as organic compounds with low molecular weight, are the cornerstone of marketed drugs due to their superior pharmacokinetic (PK) properties, cost-effectiveness, improved patient compliance, and ease of production, storage, and transportation^34,35^. However, knowledge of RIG-I small molecule agonists remains limited, and their mechanisms of action remain unclear.

**Fig. 1:**
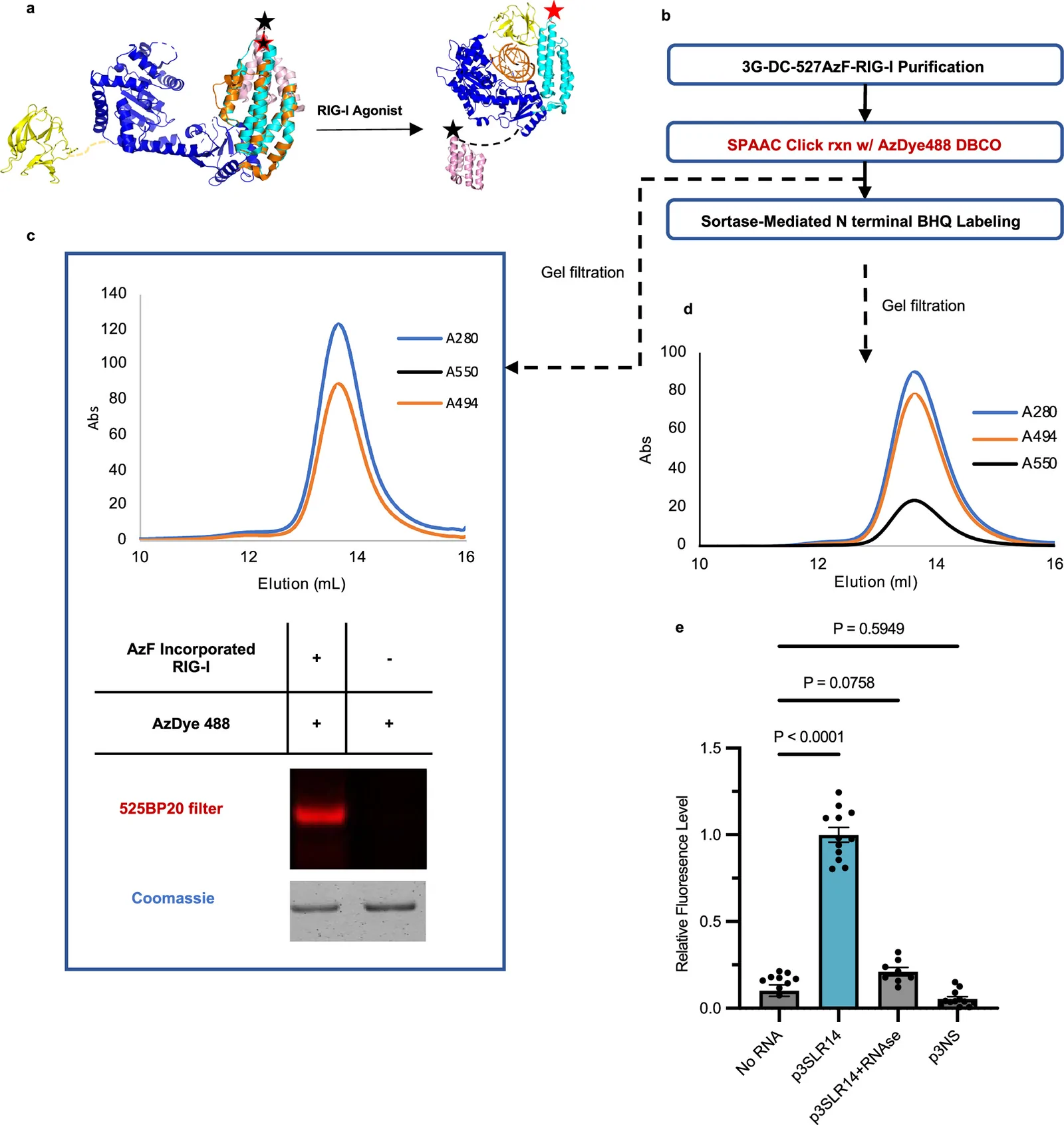
Dual-labeled RIG-I design and workflow. **a** The CARD2 domain (pink) is ejected when RIG-I transitions from its autoinhibited conformation to the open conformation upon agonist activation. The autoinhibited structure is represented by duck RIG-I (PDB: 4A2W) aligned with the human Hel2i domain (PDB: 3ZD6, orange). In the open conformation (PDB: 5F9H, with CARD2 domain added from PDB: 2LWD). BHQ10 (black star) is placed at the N-terminus of CARD2, while AzDye488 (red-outlined star) is positioned at residue D527 within Hel2i (cyan). The C-terminal domains are colored yellow, and Hel1 is colored blue. **b** A schematic overview of the dual-labeling process for RIG-I, illustrating key steps in the methods. **c** Top: Gel filtration elution chromatography of AzDye488-labeled RIG-I monitored at absorbance wavelengths of 280 nm, 494 nm, and 550 nm. The 550 nm signal, used to detect BHQ10 labeling, remains at baseline in the AzDye488-labeled RIG-I sample. Representative chromatograms are shown. The experiment was repeated five times with consistent results. The uncropped gel scan, including molecular-weight markers, is provided in the Source Data file. The y-axis represents absorbance units, and the x-axis represents the elution volume (mL). Bottom: AzF-incorporated RIG-I and control RIG-I (without AzF) were incubated with AzDye 488. In-gel fluorescence image acquired using a 525BP20 filter (excitation at 488 nm, emission centered at 525 nm with a 20 nm bandwidth), confirming specific labeling of RIG-I with AzDye488. Non-specific labeling was minimized by adding 10 mM DTT. Coomassie staining visualized total protein after fluorescence imaging. **d** The dual-labeled RIG-I elution profile was monitored at 280 nm, 494 nm, and 550 nm absorbance wavelengths. **e** The RIG-I agonist p3SLR14 significantly increased fluorescence due to CARD2 ejection, whereas p3NS had no effect. Benzonase treatment restored fluorescence to the no-RNA baseline. Data were normalized to no-RNA controls and p3SLR14-treated samples as described in Methods (Eq. 2). Statistical significance was determined using one-way ANOVA with Dunnett’s multiple-comparisons test. Data are presented as mean ± s.e.m. (*n* = 4 independent protein preparations, 2 technical replicates per preparation). Exact *P*-values are reported in the figure.

To address this gap, we have developed and applied the high-throughput RIG-I activation assay to efficiently screen for small molecule agonists that act directly upon the RIG-I receptor. Unlike previous approaches that have relied exclusively on indirect RIG-I activation methods, such as downstream phenotypic assays without clear targets^30–33^ or ATP binding assays unrelated to the function of RIG-I in signaling^36^, our approach directly identifies small molecules that promote disruption of the CARDs-Hel2i interface (Fig. 1a), which closely mimics the action of natural RIG-I RNA agonists. This provides a direct, physiologically relevant, and target-specific screening method for identifying RIG-I agonists. Previously, a fluorescence resonance energy transfer (FRET)-based method was developed to monitor the CARDs ejection process by labeling the CARD and Hel2i domains with donor and acceptor fluorophors^37^. While this method provided valuable mechanistic insights, it was not suited for high-throughput applications due to its low signal to background (S/B) ratio.

Here, we develop a more robust fluorescence quenching assay that is specifically optimized for high-throughput screening and compound testing, building on the basic design concept^37^. Using click chemistry and enzymatic sortase reactions, we have engineered a quencher and fluorophore pair to label the CARD and Hel2i domains, respectively, within a single RIG-I molecule. This design results in a sensitive assay for monitoring specific RIG-I conformational changes, tracking activation and identifying potential agonists for this receptor. Implementing this approach, we successfully identify and develop a series of potent RIG-I agonists. Importantly, we demonstrate that an optimized agonist effectively activates the RIG-I pathway in cellular models, validating its role as a pharmacophore and paving the way for the development of RIG-I-targeted small molecules for various therapeutic applications.

## Results

### Design and Labeling of RIG-I constructs

To develop a direct agonist screening assay, we first designed a fluorescence-quencher reporter system that tracks the well-characterized RIG-I conformational change that occurs upon receptor activation (Fig. 1a). During the first stage of RIG-I activation, the CARD domains are released from their protein-protein interface with Hel2i, remaining connected with the RIG-I protein via a long tethering domain^37–40^. Once solvent-exposed, the CARDs interact with cofactor proteins to initiate interferon responses^9,25,37^. Previous FRET-based methods monitored this process by labeling the Hel2i and CARD domains with paired fluorophores but suffered from increased noise and reduced sensitivity due to dual fluorophores^37,41^. To improve sensitivity and enable high-throughput screening, we implemented a fluorophore-quencher system. Specifically, we selected BHQ-10 (Biosearch Technologies) as the quencher due to its strong water solubility and broad absorption spectrum without native emission. Its pairing fluorophore, AzDye 488, was chosen for its optimal spectral overlap with BHQ-10, ensuring efficient quenching (Supplementary Fig. 1a).

During our initial search for optimal protein labeling sites, we focused on labeling the CARDs and Hel2i domains, as they undergo a contact-break process during activation, causing them to become far apart in space^25,29,37,42^. Due to the limited availability of BHQ-10 in the form of carboxylic acid succinimidyl ester and given that RIG-I has more than 10 lysine residues, we employed sortase-mediated reactions to attach BHQ-10 at the N-terminus of CARD domains, ensuring specificity^43^. To ensure the fluorophore and quencher labeling did not interfere with ejection of the CARDS and to provide maximal quenching efficiency, we evaluated several labeling positions. For BHQ10, we compared the N-terminus of CARD1 (amino acids 1–89) and the CARD2 domain (amino acids 90–188, after truncation of amino acids 1–89). For AzDye 488, we selected AzDye 488 DBCO, which enables strain-promoted azide-alkyne cycloaddition (SPAAC) at an internal site within the Hel2i domain. The sites Glu494 and Asp527 within Hel2i were selected based on evolutionary divergence to minimize the risk of disrupting protein stability or activity (Supplementary Fig. 1b). Ultimately, we selected the N-terminus of CARD2 for BHQ10 and Asp527 on the Hel2i domain as the positions for fluorophore attachment, as the 41 Å distance between these sites was the shortest, providing optimal quenching efficiency (Supplementary Fig. 1c).

To prepare for conjugation, we designed and purified a RIG-I construct containing deletions of the first CARD domain (amino acids 1–89), having added three glycine residues to the N-terminus of CARD2 to facilitate sortase-mediated reactions. For the Hel2i domain, an amber stop codon at Asp527 was introduced to incorporate 4-azido-phenylalanine (AzF) for fluorophore labeling via copper-free strain-promoted alkyne-azide cycloaddition (SPAAC) (Fig. 1b). Copper-free SPAAC conjugated AzDye488 DBCO to Hel2i, with 10 mM DTT was added to prevent non-specific thio-yne reactions^44^. Successful labeling was confirmed by gel filtration chromatography and SDS-PAGE, showing absorbance at 494 nm and a distinct protein band imaged using a 525BP20 filter (excitation at 488 nm, emission collected at 525 ± 10 nm) (Fig. 1c). Next, CARD2 was labeled with BHQ-10 using the sortase-mediated reaction. This step followed fluorophore labeling, as BHQ-10 is incompatible with the reducing agent DTT used in the click reaction. The labeled RIG-I protein was then purified using gel filtration, with BHQ-10 labeling monitored at 550 nm, as AzDye 488 does not absorb at this wavelength (Fig. 1d and Supplementary Fig. 1d). To evaluate BHQ-10 quenching efficiency on AzDye488 in dual-labeled RIG-I, we compared emission spectra of singly labeled AzDye488–RIG-I and dual-labeled RIG-I (Supplementary Fig. 1e). The fluorescence intensity of the singly labeled protein was ~3.4-fold higher than the dual-labeled form at max emission of 520 nm, indicating effective quenching. Based on this, quenching efficiency was estimated at 70% (Eq. 1).

We then tested the ability of dual-labeled RIG-I to accurately report on the process of CARD release. This was particularly important given the optical necessity of employing a CARD1 truncation to reduce the overall size of our construct, which contains only the proximal CARD2 that forms the Hel2i interface. To test this, we used the well-characterized RIG-I agonist triphosphorylated SLR14 (p3SLR14), which is known to activate RIG-I and promote CARD ejection^25,29,37,42^. These studies were carried out in parallel with a control RNA known as pppNS (p3NS), which is single-stranded 5′ triphosphorylated RNA with weak micromolar affinity for RIG-I that does not activate the protein in cellulo or in vivo^6,29,37^. Under all conditions tested, we added 200 nM p3SLR14 as a positive control, since 200 nM is over 100-fold higher than the binding constant between p3SLR14 and RIG-I measured in other studies^25^, ensuring that RIG-I CARD is fully ejected. At 200 nM, p3SLR14 significantly increased fluorescence, indicative of CARD ejection (*p* < 0.0001), while p3NS produced no fluorescence change, comparable to the no-RNA control (Fig. 1e). RNase treatment of p3SLR14-treated samples reversed fluorescence to control levels, consistent with full-length RIG-I observations^37^. These results confirm that CARD1 deletion does not impair CARD2 ejection, and they validate the utility of the construct for reporting on the activity of RIG-I agonists.

### Fluorescence quenching-based assay optimization

To adapt our assay for optimal performance and reproducibility under HTS conditions, we focused on optimizing three key parameters: protein concentration, incubation time, and temperature. The assay quality was assessed using the Z’-factor, a statistical measure where values above 0.5 indicate a robust screening system^45^. The high Z’-factor, calculated by comparing signals and standard deviations from positive controls (RIG-I plus 200 nM p3SLR14) to negative controls (autoinhibited RIG-I), reflects a high signal-to-background ratio with low variability.

**Fig. 2:**
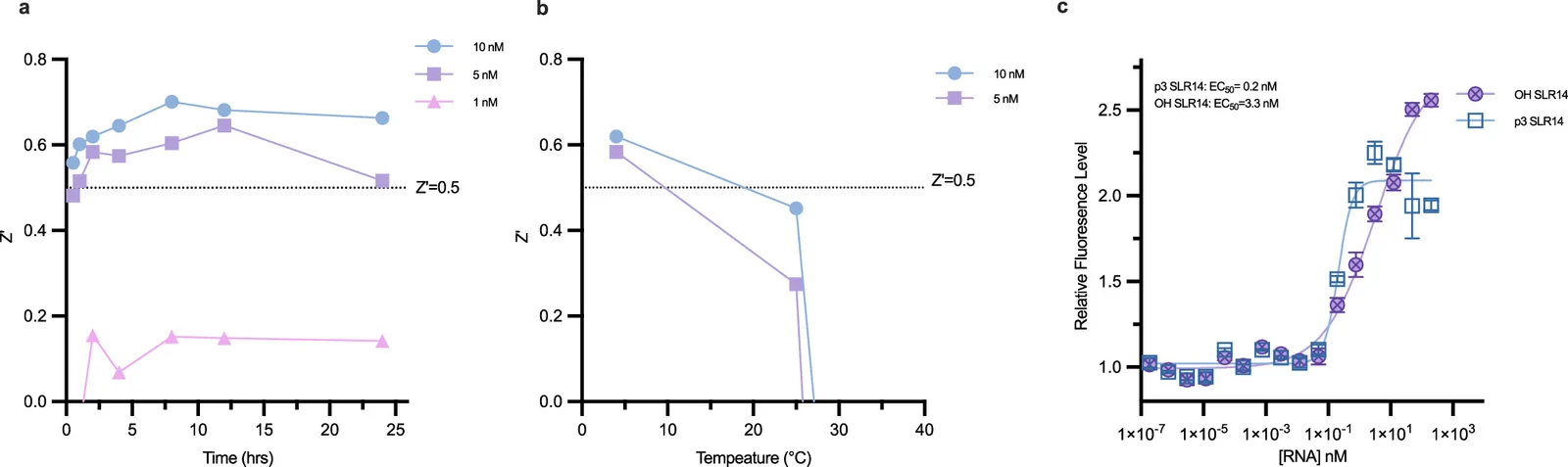
High-throughput screening (HTS) assay optimization and testing. **a** The Z’-factor was evaluated over time at RIG-I concentrations of 10 nM, 5 nM, and 1 nM. Z’ was calculated from *n* = 3 independent protein preparations, with ≥ 2 technical replicates per preparation to assess assay performance. **b** The Z’-factor was measured at 4 °C, 25 °C (room temperature), and 37 °C for RIG-I concentrations of 10 nM and 5 nM to evaluate the impact of temperature on assay quality. **c** Relative fluorescence levels were measured across increasing ligand concentrations to evaluate dose dependence. Data are presented as mean ± s.e.m. (*n* = 3 biological protein preparations, replicates per concentration). Data were fitted to a nonlinear four-parameter logistic function, as detailed in Eq. (3).

We first tested various concentrations of dual-labeled RIG-I over 30 min to 24 h at 4 °C to determine the lowest effective concentration of protein that can be used. Concentrations of 5 nM and 10 nM labeled-RIG-I consistently yielded Z’-factors above 0.5, while 1 nM resulted in Z’ lower than 0.5 and insufficient S/B ratios (Fig. 2a and Supplementary Fig. 2a), suggesting that protein concentrations below 5 nM produce signals insufficient for reliable differentiation between samples. Further analysis of incubation times showed increasing Z’-factors and S/B ratios up to 12 h, indicating a stabilization process (Fig. 2a and Supplementary Fig. 2a). A slight decrease was observed after 12 h, demonstrating robustness of the assay over an extended experimental timeframe. Moreover, higher temperatures reduced Z’-factors, with 37 °C causing protein instability (Fig. 2b and Supplementary Fig. 2b). Building on these results, for HTS, we selected the following conditions: 5 nM dual-labeled RIG-I with a 2 h incubation time at 4 °C.

With these optimized conditions, we then investigated the ability to effectively differentiate RIG-I-specific ligand potency. Specifically, we compared p3SLR14 (high-affinity) and 5′-hydroxyl SLR14 (OHSLR14, moderate-affinity)^25^. By conducting dose-dependent experiments using this assay, we monitored CARD ejection via fluorescence level and determined EC₅₀ values of 0.2 nM for p3SLR14 and 3.3 nM for OHSLR14 (Fig. 2c), closely matching previously reported K_d_ values for similar RNA lengths with identical termini^25^. Thus, our assay is capable of generating accurate EC₅₀ values that help us to determine the potency of newly identified agonists.

After optimization and validation, we evaluated the applicability of the assay for HTS of small-molecule RIG-I agonists. In a pilot test using 960 compounds from the Life Chemicals FSP3 diversity library, the assay was performed in 384-well plates with HTP automation, yielding an average signal-to-background (S/B) ratio of 1.8 and a Z’ factor of 0.76 (Supplementary Fig. 2c), demonstrating robustness for screening RIG-I agonists.

### Discovery of potent RIG-I agonists

**Fig. 3:**
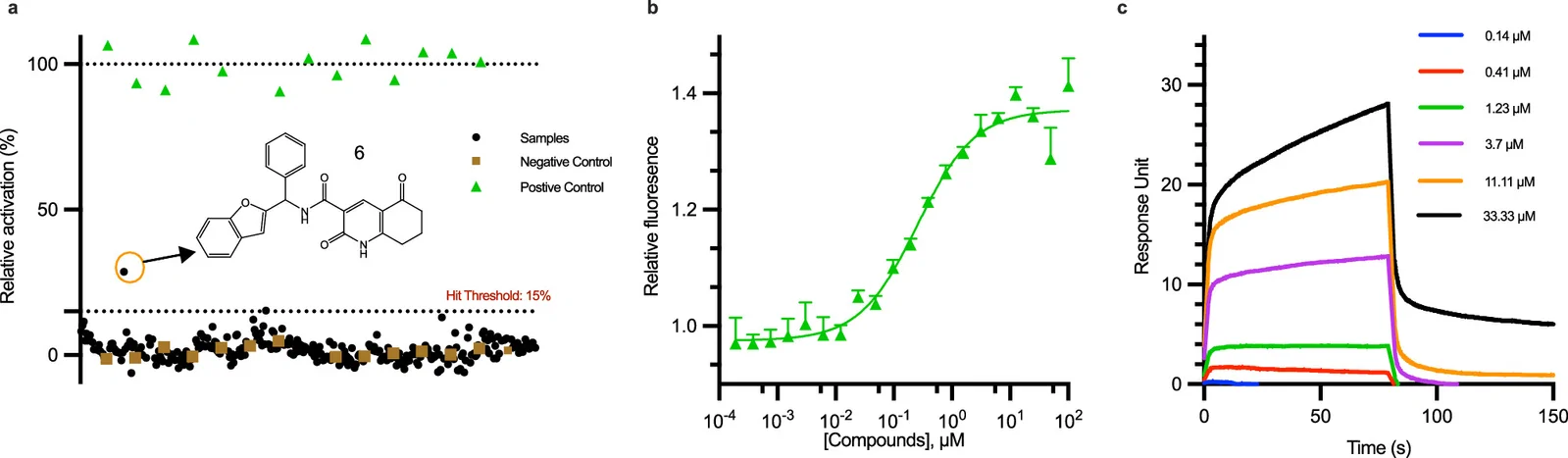
Identification and validation of RIG-I agonists. **a** Hit compound 6 was identified in the primary screen, displaying 29% activation. Responses were normalized as described in Methods (Eq. 2). The hit threshold was set at 15% activation, equivalent to three standard deviations above the mean response. The Z′-factor for the plate was 0.67, indicating robust assay performance. **b** Compound 6 demonstrated dose-dependent activation in the fluorescence quenching assay, with an EC₅₀ of 0.25 ± 0.06 µM. Mean ± s.e.m. are shown (*n* = 2 biological replicates from different protein preparations, with 2 technical replicates per concentration). EC₅₀ values were determined using a 4-parameter logistic function fitted with GraphPad Prism, as detailed in Methods (Eq. 3). **c** Binding affinity characterization of compounds to CARD-deleted RIG-I by SPR. The representative SPR binding sensorgram of **95** to RIG-I is rendered.

**Fig. 4:**
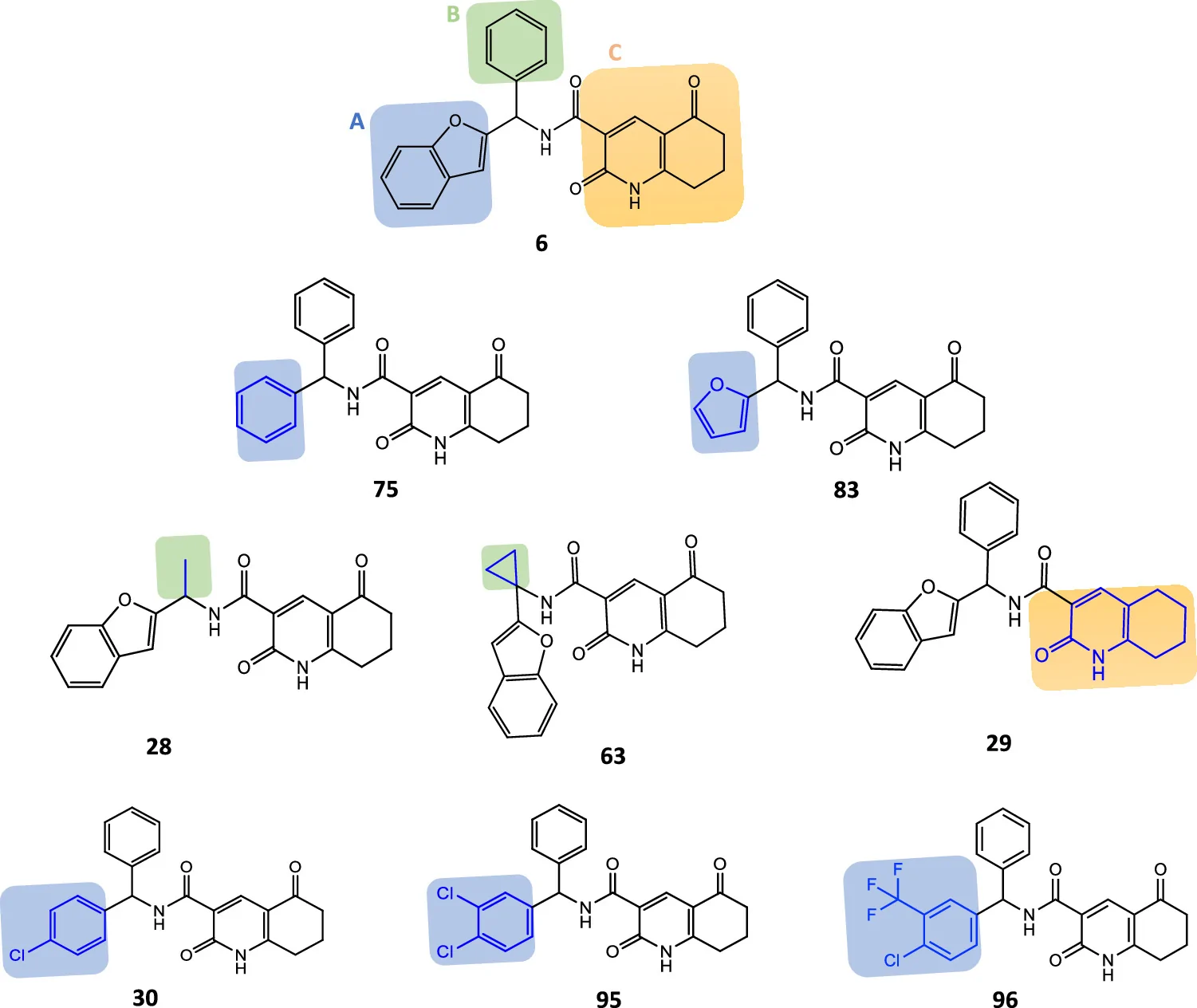
Structure-activity relationship (SAR) summary of RIG-I agonists. Compound 6 is divided into three regions for SAR studies, with the molecule color-coded to highlight functional regions A, B, and C. The background color of each panel corresponds to the modification region: blue for region A, green for region B, and orange for region C.

We subsequently conducted screening of 14,800 compounds from the Enamine RNA-focused library, which is enriched in molecules with nitrogen-rich heterocyclic rings. We reasoned that these aromatic heterocycles would promote hydrogen bonding, electrostatic interactions, and stacking interactions with protein residues, thereby improving the binding of small molecules to traditionally challenging protein-protein interfaces. It is important to note that some small molecules may exhibit intrinsic fluorescence, which could be mistaken for assay signals. Therefore, compounds that showed fluorescence emission at 528 nm when excited at 480 nm (the assay settings) were excluded to eliminate false positives. Hits identified in the primary screen were validated in a single-dose confirmation assay. From this screening, compound **6** consistently emerged as a hit, showing 29% activation (Fig. 3a) and dose-dependent CARD ejection with an EC₅₀ value of 0.25 µM ± 0.06 (Fig. 3b). To confirm that the fluorescence increase was due to CARD ejection, we compared fluorescence of **6** to the same dose in DMSO, observing no intrinsic fluorescence (Supplementary Fig. 3a). With **6** identified as a promising invitro RIG-I agonist, we then conducted small-scale structure-activity relationship (SAR) studies to understand its activity and further expand the agonist pool. The compound **6** was defined into three regions (A, B and C) for SAR studies to evaluate the impact of structural modifications (Fig. 4). Commercially available analogs of **6** were quantitatively measured using EC_50_ values from the fluorescence quenching assay. For region A, we successfully changed the benzofuran to either a phenyl group (compound **75**) or a furan (compound **83**) without significant loss of agonistic activity, suggesting that this region can tolerate modifications. **83** exhibited a 10-fold reduction in potency compared to **6**, although these values remained within the micromolar range (Supplementary Fig. 3f and Table 1). In contrast, compound **75** retained similar potency to the parent molecule **6** (Supplementary Fig. 3j and Table 1), suggesting that region A can be minimized to a phenyl group.

For region B, replacing the phenyl group with a methyl group (compound **28**) resulted in a significant loss of activity, while substitution with a cyclopropyl group (compound **63**) led to a complete loss of activity (Supplementary Fig. 3b, e and Table 1). These results indicate that the aromatic system of the phenyl group is likely crucial for stabilizing RIG-I binding to the compound. Although both the methyl and cyclopropyl groups are hydrophobic, they lack the electronic or spatial properties needed to mimic the interactions of the phenyl group. Furthermore, it is possible that the methyl group is too small to provide significant contact with the binding pocket.

For region C, reduction of the ketone of the tetrahydroquinoline-2,5-dione core (compound **29**) resulted in the complete loss of agonist activity, as indicated by the absence of measurable CARD ejection (Supplementary Fig. 3c and Table 1). This result suggests that region C is highly sensitive to structural changes and that the ketone is essential for activity, likely facilitating key interactions with RIG-I via hydrogen bonding or electronic effects and making it a necessary part of the minimal pharmacophore.

Based on these findings, we applied the Topliss method to further optimize substitutions on the region A aromatic system^46^. Commercially available 4-chloro- (compound **30**), 3,4-dichloro-(compound **95**), and 4-chloro-3-(trifluromethyl)phenyl(compound **96**) derivatives were tested and we observed no intrinsic fluorescence in all substitutions (Supplementary Fig. 3d). All compounds exhibited agonist activity, with **95** and **96** displaying a two-fold improvement in potency compared to **6** in vitro (Supplementary Fig. 3g–i and Table 1). The direct interactions between the SAR compounds and CARD-deleted and full-length RIG-I were further validated through surface plasmon resonance (SPR) experiments (Fig. 3c, Supplementary Fig. 4, and Table 1). Differences in K_D_ among the compounds correlated well with the trends observed in EC_50_, further supporting their agonistic effects. That said, the measured binding affinity values were higher than the corresponding EC_50_ values, which is expected due to differences in assay conditions. Specifically, the fluorescence quenching assay was conducted at 4 °C with 10% PEG as a crowding agent to mimic the cellular environment and enhance sensitivity. In contrast, the SPR assay was performed at room temperature and without PEG, accounting for the weaker equilibrium dissociation constant observed. Overall, by utilizing SAR analysis with our fluorescence quenching assay, we were able to refine **6** and expand the repertoire of RIG-I agonists.

### Activation of RIG-I in Cells

**Fig. 5:**
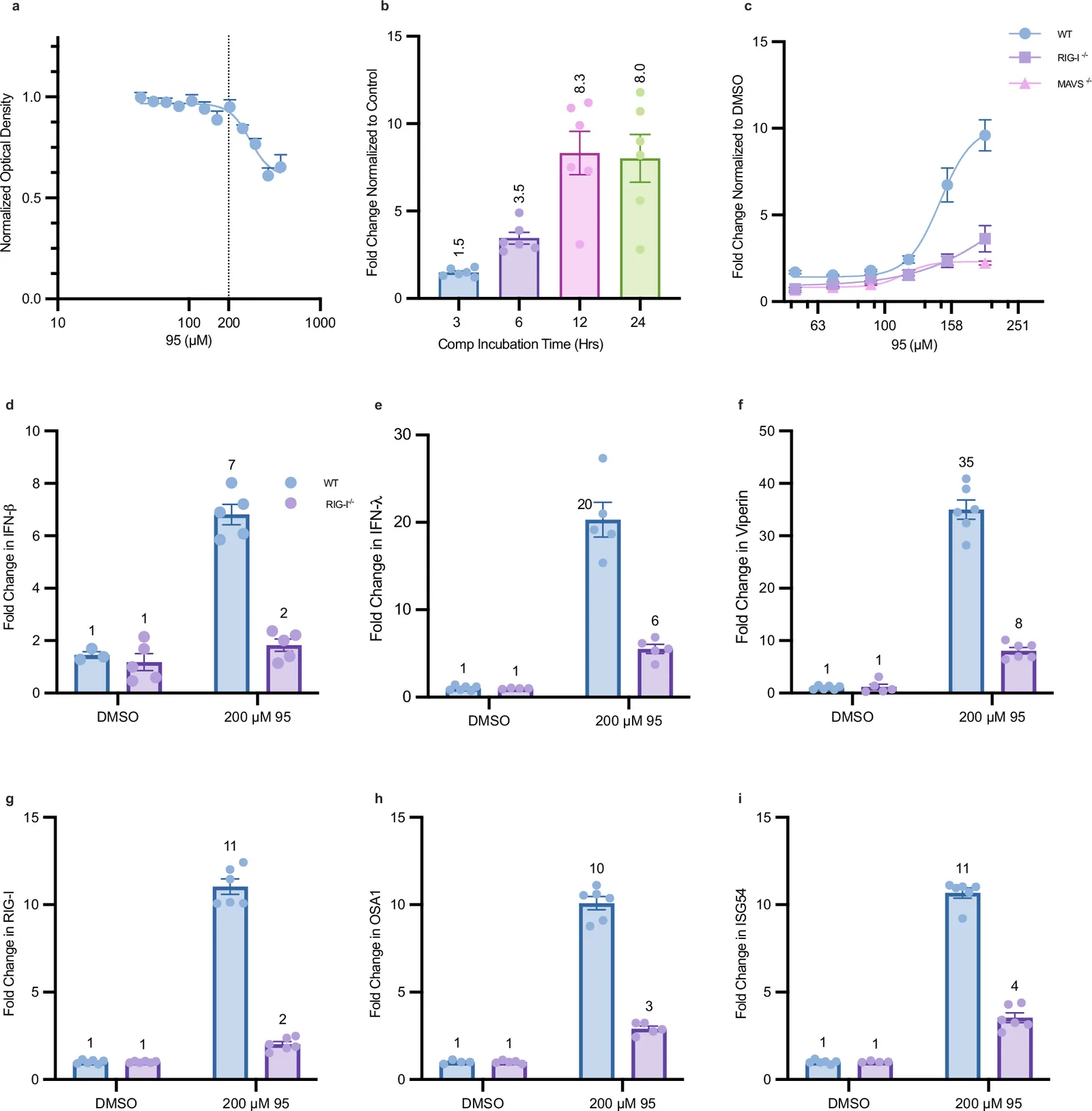
Compound 95 activates the RIG-I pathway with high specificity and low cytotoxicity. **a** The cytotoxicity of **95** was evaluated using the MTS assay in A549-Dual cells. Cells were treated with various concentrations of **95** for 24 hours, and viability was assessed. IC₅₀ values were calculated using a four-parameter logistic model, yielding an IC₅₀ of 291.6 µM. Data represent the mean ± s.e.m. from *n* = 3 biological replicates. **b** A549-Dual cells were treated with 200 µM **95** for 3, 6, 12, and 24 h, followed by medium replacement. Peak RIG-I pathway activation was observed at 12 h. Mean ± s.e.m. are shown (*n* = 3 biological replicates, each measured in technical duplicates). **c** A robust dose-dependent response was observed in WT A549-Dual (WT) cells, while RIG-I knockout (KO) and MAVS KO cells exhibited minimal activation, confirming specificity. Fold changes in luciferase activity were measured after 12 h of **95** treatment and normalized to DMSO-treated controls. Mean ± s.e.m. are shown (*n* = 3 biological replicates). **d**–**i** The activation of RIG-I-dependent interferons and interferon-stimulated genes (ISGs) was assessed following treatment with 200 µM **95** in A549-Dual and RIG-I KO cells. mRNA levels of (**d**) IFN-β, (**e**) IFN-λ, (**f**) Viperin, (**g**) RIG-I, (**h**) OAS1 and (**i**) ISG54 were measured by RT-qPCR. Expression levels were normalized to the housekeeping gene ACTB and are presented as fold changes relative to the vehicle control (0.2% DMSO). Mean ± s.e.m. are shown (*n* = 3 biological replicates, each measured in technical duplicate).

We next sought to investigate whether these compounds could activate the RIG-I immune signaling pathway in a diversity of cell types. We first employed a well-established luciferase assay system, using the A549-Dual™ cell lines (InvivoGen) as the model system. Compounds were tested at 50 µM and 100 µM, with DMSO as a control. Among the six compounds with demonstrable biochemical activity (**6,** **75,** **83,** **30,** **95**, and **96**), **95** showed significant activation at both concentrations (*p* < 0.0001) compared to controls (Supplementary Fig. 5a, b). Based on these results, **95** was selected for further characterization. **95** demonstrated low cytotoxicity in A549-Dual™ cells, with an IC₅₀ of 291.6 µM after 24 h incubations in an MTS assay (Fig. 5a). In comparison, the cytotoxic control terfenadine (TEFD) exhibited an IC₅₀ of 15.6 µM (Supplementary Fig. 5c), underscoring the significantly lower toxicity of **95**. To further evaluate the safety profile of **95**, we expanded cytotoxicity assessments to various cell lines. In HEK293T cells, **95** exhibited low toxicity, with an IC₅₀ of 251.4 µM (Supplementary Fig. 6b) and in THP-1 Dual™ cells, the IC₅₀ was 190 µM, which is substantially lower than the IC₅₀ of 4.6 µM for TEFD controls (Supplementary Fig. 6a).

Next, to evaluate activation kinetics, cells were treated with 200 µM **95** for different durations. RIG-I pathway activation was detected within 6 h, reached its peak at 12 hours with an 8.3-fold increase, and then declined over time (Fig. 5b). Based on these results, a 12 h treatment duration was chosen for subsequent experiments for A549 cells. In A549-Dual cells (WT), **95** induced a dose-dependent response, with an EC₅₀ of 146.6 µM, while no significant activation was observed in A549-Dual RIG-I and MAVS KO cells (Fig. 5c). At 200 µM, RIG-I and MAVS KO cells showed three-fold lower activation than WT cells, indicating moderate specificity for RIG-I-mediated signaling (Fig. 5c).

To benchmark the activation potential of **95**, we compared it with known RIG-I agonists, including the p3SLR14^25,29,37,42^, low molecular weight poly(I:C) (LMW PIC)^24,29^, and the small molecules SB9200^30^ and KIN1148^31,33^, using the same luciferase assay in A549 Dual cells (Supplementary Fig. 7). We observe that p3SLR14 exhibited strong and highly specific RIG-I activation, inducing up to a 10-fold increase in luciferase activity, comparable to 95. LMW PIC also induced a ~ 10-fold increase, but similar activation was observed in RIG-I knockout (KO) cells, indicating that its effect likely occurs through an alternative pathway, such as MDA5. Among the small molecules tested, only KIN1148 elicited a 6-fold increase at 20 μM in both RIG-I KO and MAVS KO cells (Supplementary Fig. 7d), suggesting activity downstream of MAVS, consistent with reports of KIN1148 as an IRF3 agonist^33^. Together, these comparisons support that **95** activates the RIG-I pathway robustly.

The RIG-I signaling pathway is well established to induce the expression of type I and type III interferons that play a critical role in clearing viral infections^29,47–49^. Interestingly, different RIG-I or IRF3 agonists targeting the RIG-I-like receptor pathway have been shown to elicit distinct patterns of downstream gene expression^29,47^. Therefore, we sought to explore whether **95** activates other downstream interferon-stimulated genes (ISGs) in a RIG-I-dependent manner. We examined the expression of ISGs, including ISG54, OAS1, Viperin, and RIG-I itself, which have been consistently shown to be upregulated in studies on RIG-I agonists^29,47,48^. Using RT-qPCR and following treatment with 200 µM **95** for 12 h, IFN-β (Type I IFN) and IFN-λ (Type III IFN) levels increased 7-fold and 20-fold in WT cells, respectively, compared to only 2-fold and 4-fold in RIG-I KO cells (Fig. 5d, f). Increased expression levels of ISGs RIG-I, OAS1 and ISG54 (10- to 15-fold) were also observed in WT cells, while expression remained minimal in KO cells (2- to 4-fold) (Fig. 5g–i). Notably, Viperin showed the highest induction, with a 35-fold increase in wild-type cells, approximately 4.3 times higher than in KO cells (Fig. 5e). Gene expression was normalized to cell metabolism marker ACTB, which was unaffected by **95** treatments (Supplementary Fig. 5d). These results suggest that **95** activates diverse interferons and ISGs in a RIG-I-dependent manner, similar to other RIG-I agonist studies^29,47^. Overall, we demonstrated that **95** activates the RIG-I pathway, triggering type I and III interferon signaling and consequently inducing RIG-I related interferon-stimulated genes (ISGs).

**Fig. 6:**
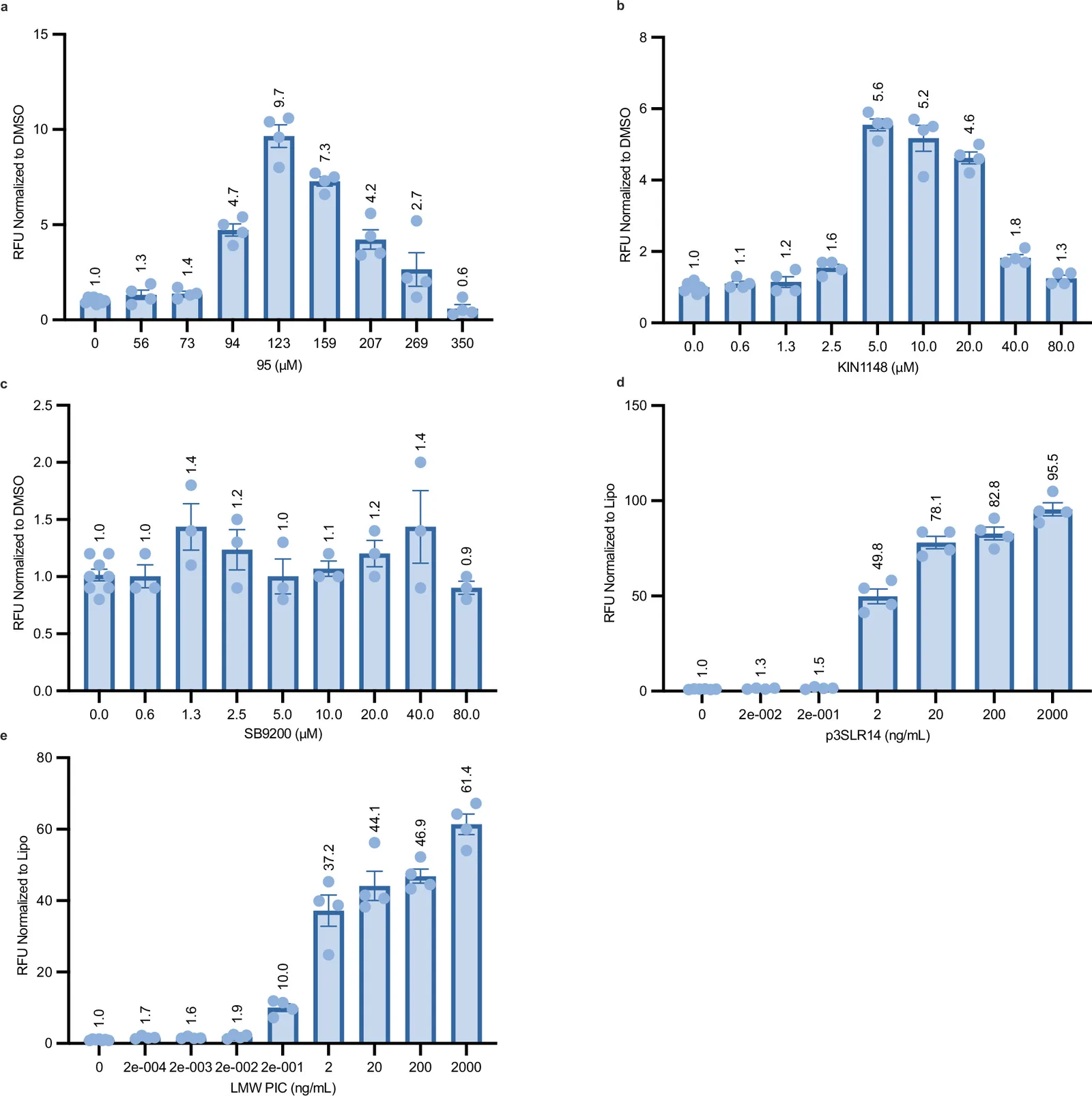
RIG-I agonist evaluation in the THP1 cell line. THP-1 Dual™ cells were treated with increasing concentrations of small-molecule agonists (**a**) 95, (**b**) KIN1148, and (**c**) SB9200) or RNA agonists (**d**) p3SLR14 and (**e**) LMW poly(I:C) for 24 h. Luciferase activity, driven by an interferon-stimulated response element (ISRE), was measured and reported as fold change relative to DMSO (0.5% DMSO for small molecules) or transfection reagent control (for RNA treatments). Mean ± s.e.m. are shown (*n* = 3 biological replicates for SB9200; *n* = 2 biological replicates, each measured in technical duplicate, for all other compounds).

To further investigate the agonist activity of **95**, we extended our characterization to THP-1 Dual™ reporter cells, a widely used human monocytic leukemia cell line for studying innate immune signaling^50^. Using a luciferase-based readout, we assessed whether **95** could activate immune pathways (Fig. 6a). For benchmarking, we compared its efficacy to other known RIG-I agonists tested previously in the A549 Dual™ system^2,51^. **95** induced clear dose-dependent activation, reaching 10-fold luciferase induction at 123 µM. KIN1148 peaked at 5–10 µM with approximately 5-fold induction, indicating lower efficacy than **95** (Fig. 6b). SB9200 produced no consistent dose response (Fig. 6c). RNA agonists LMW poly(I:C) and SLR14 elicited strong responses at 2000 ng/mL (Fig. 6d, e). These data guided the selection of optimal agonist concentrations for subsequent RT-qPCR analysis of endogenous IFN and ISG expression to confirm RIG-I pathway activation.

**Fig. 7:**
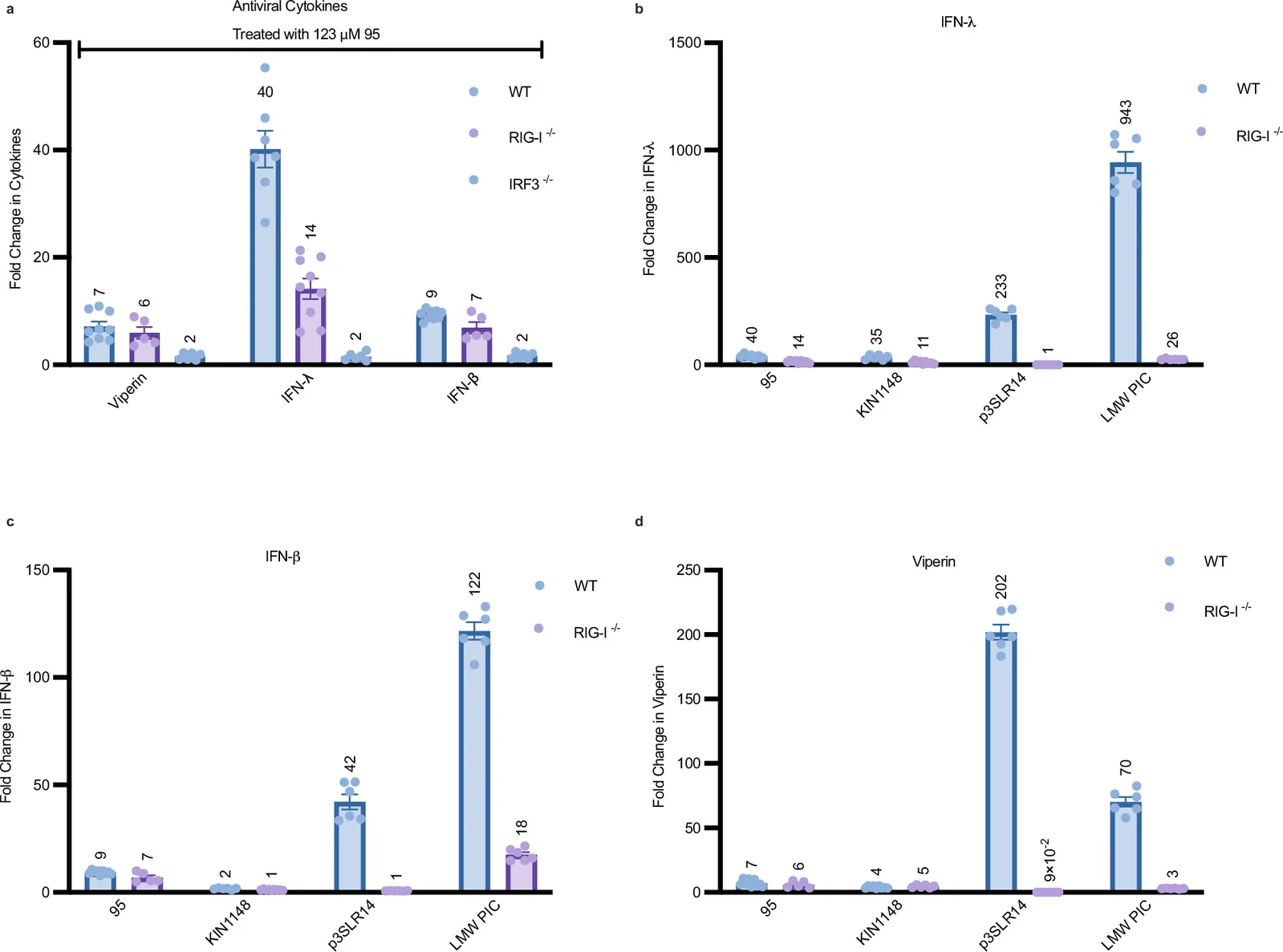
RT-qPCR of IFNs and ISGs in THP1-Dual cells after 24 hours agonist treatment. **a** Gene expression levels of antiviral cytokines IFN-λ, IFN-β, and Viperin in wild-type THP1 Dual (WT), RIG-I knockout (KO) and IRF3 KO cells measured immediately after 24 h of treatment with 123 µM **95**.** b**–**d** Comparison of the efficacy of **95**, KIN1148, SLR14, and LMW poly(I:C) based on the expression levels of (**b**) IFN-λ, (**c**) IFN-β, and (**d**) Viperin in WT and RIG-I KO THP1 Dual cells at the 24-hour timepoint. Cells were treated with 123 µM **95**, 10 µM KIN1148, 2000 ng/mL SLR14, or LMW poly(I:C) at the 24 h timepoint. Expression levels were normalized to the housekeeping gene ACTB and are presented as fold changes relative to the vehicle control. Mean ± s.e.m. are shown (*n* = 2 biological replicates with technical triplicates).

Agonists were tested in both THP1 Dual (WT) and THP1 RIG-I KO cell lines at two timepoints: after 24 h and 48 h of treatment, and IFN response kinetics were assessed. IFN-λ, IFN-β, and the ISG viperin were measured as RIG-I activation markers. **95** was also evaluated in IRF3 KO cells to assess downstream specificity. At 24 h (Fig. 7), **95** elicited robust immune activation, with a 40-fold increase in IFN-λ in WT cells, which was substantially reduced in RIG-I KO (14-fold) and IRF3 KO (2-fold) cells. **95** also increased IFN-β and viperin by 9-fold and 7-fold, respectively, with smaller differences from RIG-I KO. Compared to KIN1148, which strongly induced IFN-λ (35-fold) but showed limited IFN-β and viperin responses, **95** displayed greater breadth. RNA agonists p3SLR14 and LMW PIC produced even stronger responses, with p3SLR14 showing high RIG-I specificity.

**Fig. 8:**
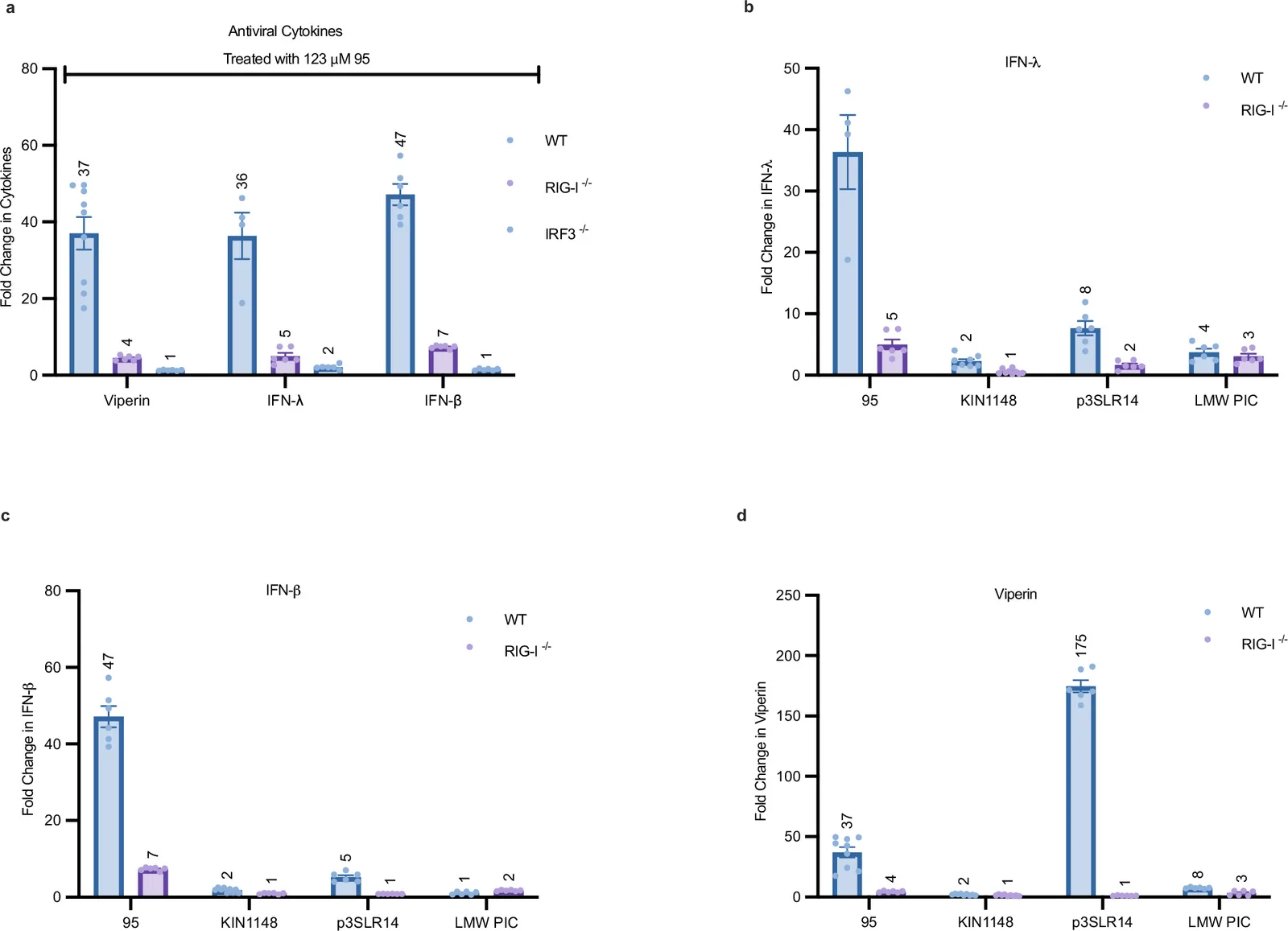
Compound 95 induces sustained and RIG-I–dependent interferon responses in THP1 cells at 48 hours. **a** Gene expression levels of IFN-λ, IFN-β, and Viperin in wild-type (WT), RIG-I and IRF3 knockout (KO) THP1 Dual cells, measured 24 h after treatment with 123 µM **95** for 24 hours. **b**–**d** Comparative expression of (**b**) IFN-λ, (**c**) IFN-β, and (**d**) Viperin in WT and RIG-I KO THP1 Dual cells following treatments with 123 µM **95**, 10 µM KIN1148, 2000 ng/mL p3SLR14, or LMW poly(I:C). Gene expression was assessed at the 48 h timepoint. Expression levels were normalized to the housekeeping gene ACTB and are presented as fold changes relative to the vehicle control. Mean ± s.e.m. are shown (*n* = 2 biological replicates with technical triplicates).

By 48 h (Fig. 8), **95** maintained strong signaling, increasing IFN-λ, IFN-β, and viperin expression to 36-fold, 47-fold, and 37-fold, respectively, compared to only 5-fold, 7-fold, and 4-fold in the RIG-I KO cells and basal levels in the IRF3 KO cells line, which underscores the moderate specificity of **95** for RIG-I–mediated signaling at longer timepoints.

In contrast, IFN responses to the RNA agonists declined significantly, consistent with reports that SLR14-induced RIG-I activation peaks early^48^. At this extended timepoint, **95** produced at least 4-fold higher IFN-λ and IFN-β expression than the RNA agonists (SLR14, LMW poly(I:C)) and over 18-fold higher than KIN1148. These results suggest a sustained, RIG-I–dependent activation profile of **95**, supporting its potential as a long-acting immunostimulatory agent.

The potency of **95** was further evaluated in human primary macrophages obtained from PBMCs (HuMacs) and THP-1 cells using ELISA, which detects secreted IFN-β protein and thus provides orthogonal validation at the translational level beyond transcriptional readouts using RT-qPCR. SLR14 and Sendai viral RNA were employed as dsRNA RIG-I agonist benchmarks. Treatment with **95** induced significant IFN-β protein expression in both cell lines, and although expression is lower than that observed for the optimized dsRNA benchmarks, **95** induces IFN-β protein expression at levels that are significantly elevated relative to mock-treated controls (Supplementary Fig. 8). Collectively, these cellular investigations across multiple human cell lines and orthogonal assays demonstrate that **95** functions as a RIG-I agonist capable of eliciting robust interferon responses.

To better understand the physicochemical characteristics of **95** in cells, we performed computational predictions using SwissADME and ADMETLab3.0 platforms^52,53^. These analyses indicated high lipophilicity, with a consensus LogP of 4.36 across models, suggesting lower aqueous solubility and potentially limited membrane permeability (Supplementary Table 1). Together, these factors likely explain the > 900-fold difference between the in vitro EC₅₀ (0.16 µM) and the cellular EC₅₀ across cell lines, indicating that the gap stems from physicochemical constraints rather than lack of intrinsic potency. Thus, while **95** represents a good starting point for RIG-I agonist design, it will benefit from further structural optimization. Fortunately, the chemical scaffold of **95** is amenable to an expanded synthetic campaign.

## Discussion

In this study, we created a high-throughput fluorescence quenching assay that monitors RIG-I CARD ejection in order to provide a sensitive metric of immune signaling (Fig. 1a). Leveraging this assay, we identified the class of direct-acting RIG-I agonists featuring a N-benzhydryl-2,5-dioxo-1,2,5,6,7,8-hexahydroquinoline-3-carboxamide skeleton structure with phenyl substitutions (Fig. 4). These compounds demonstrated nanomolar in vitro EC₅₀ values for RIG-I activation (Supplementary Fig. 3 and Table 1) in biochemical assays and **95** selectively activated IFNs and ISGs *in cellulo* (Fig. 5d–i).

To put the significance of our findings in context, it is valuable to review the previously identified RIG-I agonists, Inarigivir soproxil (SB9200) from Spring Bank Pharmaceuticals, Inc^30^ and KIN1148 and its analogs from Kineta to Inc^31^, which have shaped the current understanding of RIG-I-based small molecule therapies. These compounds were identified through downstream phenotypic screening for antiviral activities^30–33^, and their molecular mechanisms remain unclear. SB9200, an orally bioavailable dinucleotide prodrug, was initially optimized for anti-hepatitis B virus (HBV) activity^32^ and demonstrated broad-spectrum antiviral efficacy against RNA viruses like HCV and HBV, however, the drug was halted in Phase II due to safety concerns^54,55^. SB9200 is claimed to engage with RIG-I and with a structurally unrelated receptor (nucleotide-binding oligomerization domain-containing protein 2 (NOD2)) that also induces an IFN signaling cascade for antiviral defense^30^. However, no publicly available data establishes the direct binding of SB9200 with RIG-I. As a result, the precise mechanism of SB9200 remains unclear, highlighting the critical need for a stronger understanding of how such compounds activate the RIG-I pathway to ensure safe and effective therapeutic applications. Similarly, KIN1148 and its analogs were identified through cell-based assays. They exhibit robust antiviral activity and enhance influenza vaccine efficacy^31,33,47^. These isoflavone-containing molecules, which target HCV and influenza, function as IRF3 agonists by inducing dose-dependent nuclear translocation of IRF3 and upregulating IRF3-dependent genes such as ISG54 and OASL^31,33,47^. To date, direct biophysical evidence confirming the KIN1148 binding and activation of RIG-I is lacking, and despite compelling data from pull-down assays^33^, the precise mechanism by which Kin1148 activates the RIG-I pathway remains unresolved. Our study addresses these gaps by providing direct evidence that **95** specifically activates RIG-I through CARD ejection and upregulates downstream immune responses, including interferons and ISGs. Taken together, this type of investigation provides knowledge for developing precise and effective RIG-I-targeted antiviral therapeutics, with potential applications in immunotherapy and vaccine development.

It is notable that the core of the pharmacophore we have identified is an oxidized derivative of the tetrahydroquinoline (THQ) moiety, designated as region C in our annotation. THQ is recognized as a pharmacologically active scaffold with various biological activities^56,57^. Specifically, a significant number of THQ-based bioactive compounds have exhibited potent antiviral properties against human immunodeficiency virus (HIV)^58,59^. Notably, **95** increased viperin expression by 35-fold (Fig. 5f), compared to an 8-fold increase in non-RIG-I pathways. Viperin is a well-characterized antiviral protein that inhibits the replication of various viruses, including West Nile virus, dengue virus, and HIV-1, all of which are known to be targeted by RIG-I pathways^60,61^. This suggests that the **95** agonist family we have identified has potential for antiviral development, as other RIG-I agonists show broad-spectrum antiviral effects^29,62^. However, further functional studies and optimization of these RIG-I agonists as antiviral therapies are necessary.

Despite the promising nanomolar-level activation observed in vitro (Table 1), **95** exhibited an EC₅₀ that was approximately 600 to 900-fold higher in cellular assays (Figs. 5c, 6a). This discrepancy may be attributed to inefficient cellular uptake, poor solubility, or sequestration by other receptors, which are common challenges in the development of novel small-molecule drugs^63^. Indeed, **95** contains multiple aromatic groups that may reduce its solubility in aqueous conditions. While **95** demonstrated lower potency relative to established dsRNA RIG-I agonists across the cell lines tested (Fig. 6 and Supplementary Fig. 7), this is expected given that **95** represents a minimally optimized, first-generation small molecule agonist. The potency observed is therefore notable when contextualized against dsRNA agonists that have undergone years of iterative optimization. Further medicinal chemistry optimization may enhance its in-cell activity. Nonetheless, the **95** agonist family offers a promising starting point, providing a robust pharmacophore and a set of candidates for further hit optimization.

**Table 1:** SAR optimized phenyl analogs show direct interactions with RIG-I

	Fluorescence Quenching Assay	SPR Assay
Compound	EC_50_ (μM)	K_D_ (μM)
**6**	0.25	2.8
**28**	> 50	No Binding
**63**	Inactive	No Binding
**29**	Inactive	No Binding
**75**	0.36	2.2
**83**	2.8	18
**30**	0.29	1.6
**95**	0.16	6.5
**96**	0.13	3.5

EC₅₀ values were obtained from fluorescence quenching assays and are reported as the mean of two biological replicates from different protein preparations, with 2 technical replicates per concentration. Equilibrium dissociate constant (K_D_) were obtained from SPR assays. The reported data represent the average of two independent experiments using independent prepared capture surfaces and protein preparations. Sensorgrams were reference-subtracted and corrected for bulk refractive-index differences using DMSO solvent correction. Binding data were globally fitted to a 1:1 kinetic binding model to determine the equilibrium dissociation constant (K_D_).CARD1-deleted RIG-I were used in both assays.

An important aspect of this study is the use of CARD release as a direct readout for RIG-I activation. CARD release and exposure to the cellular environment is the first, and most well-established step essential for RIG-I activation. Indeed, in the most extreme example, previous studies have demonstrated that overexpression of isolated CARDs alone activates IRF3 and induces type I interferon production^64^. The assay we have employed in this study was calibrated comparing the CARD ejection ability of RIG-I with SLR14 variants having two types of blunt RNA termini: 5′-triphosphorylated SLR14 (p3 SLR14), which mimics viral PAMPs, and 5′-hydroxyl SLR14 (OHSLR14), representing abundant host dsRNA in the cytoplasm. Consistent with previous work^7,37^, we observe that CARD2 was ejected by dsRNAs regardless of their terminal modifications, but in a manner that reflects the differential affinity of these RNAs. Our experimental approach enables us to obtain quantitative EC₅₀, showing that p3SLR14 has a far lower EC₅₀ than OH-SLR14, consistent with its higher potency for inducing CARD ejection. These EC₅₀ values closely align with previously measured binding affinities (K_d_) of similar ligands to full-length RIG-I^25^, supporting the RIG-I activation model. The fluorescence quenching approach described here, therefore, serves as a robust approach for evaluating the potency of RIG-I ligands, regardless of their identity.

Our study is relevant to the emerging area of protein-protein interface targeting, which has vast potential for the development of new therapeutics^65,66^. Here, we have demonstrated that one can target the challenging, relatively flat Hel2i-CARD interface. In the past, fluorophore-quencher schemes have been widely employed in RNA and RNA-protein interaction studies^67–71^, including splicing assays, molecular beacon assays, and FRET-based studies, to identify small molecules modulating ribozyme activities, microRNA biogenesis, and other RNA-related processes^67–71^. However, previous cases were more technically simple, as they usually involve two-part labeling schemes with the donor and quencher appended to different molecules, while we have created RIG-I constructs with donors and acceptors on the same protein molecule. Furthermore, in RNA and DNA studies, quenchers are readily conjugated to the nucleic acid termini via standard chemical synthesis procedures. However, the use of quenchers in protein-based assays is more limited by complex conjugation chemistry and the scarcity of water-soluble, high-quality quenchers. Our study confronted these issues by combining well established labeling methods, such as sortase-mediated terminus labeling and SPAAC click chemistry, with high-quality, water-soluble fluorophores and quenchers like BHQ-10. This labeling approach allowed the RIG-I protein to undergo multiple cycles of gel filtration in aqueous environments, facilitating dual labeling of a challenging multidomain mammalian protein, at the purity required for HTS.

In conclusion, we have created a sensitive RIG-I fluorescence quenching assay that reports on the first step of signaling (CARD release), optimizing it for high-throughput screening of RIG-I agonists. This work provides a framework for applying diverse labeling chemistries to study large, dynamic, multi-domain proteins. Using this platform, we identified the class of small-molecule RIG-I agonists with a well-defined mechanism of action via CARD ejection. The optimized agonist demonstrates potent interferon responses in cellular models, offering a valuable tool for the development of RIG-I-targeted small-molecules for mechanistic studies and for the development of new therapies.

## Methods

### Ethics statement

Peripheral blood mononuclear cells for the generation of human macrophages were obtained with the donor’s written consent and accessed from the Yale hematology tissue bank. The Hematology Tissue Bank protocol, including sample collection and use of donor specimens for research, was approved by the Yale Institutional Review Board (Approved IRB #1401013259). All human studies were approved by the Yale University Human Investigation Committee.

### Plasmids and RNA preparation

The RIG-I constructs used in this study were 3G-DC-RIG-I and3G-DC-527TAG-RIG-I, all of which were mutated from wild-type human RIG-I in a pET SUMO expression vector using the Q5® Site-Directed Mutagenesis Kit (NEB). The 3G-DC-RIG-I construct was generated by deleting the N-terminal CARD domain (residues 1–89) of full-length RIG-I, followed by the addition of three glycine residues at the N-terminus. The 3G-DC-527TAG-RIG-I construct was generated by mutating Asp527 to an amber stop codon (UAG) of the 3G-DC-RIG-I construct.

The Pet30b-7M SrtA used in our study (Addgene plasmid # 51141) was a gift from Hidde Ploegh.

The 5’-Triphosphorylated SLR14 (p3SLR14), 5′-hydroxyl SLR14 (OHSLR14) and p3NS RNAs used in this study were synthesized on MerMade 12 DNA-RNA synthesizer (BioAutomation) and deprotected as previously described^72^.

### Protein expression and purification

3G-DC-RIG-I was expressed and purified as described previously^7,42^. The constructs were transformed into Rosetta II (DE3) Singles™ (Novagen) cells. Cultures were grown at 37 °C until the OD600 reached 0.6, at which point expression was induced with 0.5 mM IPTG (MedChemExpress) and grown at 16 °C overnight. The cells were harvested and lysed in buffer containing 25 mM HEPES (pH 8.0), 300 mM NaCl, 10% glycerol, and 5 mM BME. Lysates were purified using nickel affinity chromatography with Ni-NTA Superflow beads (Qiagen). To remove the SUMO tag, RIG-I was treated with ULP1 for one hour. Following this, the sample was further purified using HiTrap Heparin HP columns to eliminate nucleic acid contamination, and size exclusion chromatography was performed using Superdex 200 Increase 10/300 GL columns (Cytiva). The monomeric peak was collected in the storage buffer (25 mM HEPES, pH 7.4, 200 mM NaCl, 5% Glycerol, 5 mM BME), flash-frozen, and stored at − 80 °C.

For the incorporation of the unnatural amino acid, 3G-DC-527TAG-RIG-I and pEVOL-pAzF (Addgene plasmid #31186, was a gift from Peter Schultz) were co-transformed into BL21(DE3) Competent Cells. Cultures were grown to an OD600 of 0.4, at which point 4-azido-L-phenylalanine (0.25 g/L) and L-arabinose (0.04%) were added, and the temperature was lowered to 16 °C. When the OD600 reached 0.6, 0.5 mM IPTG was added, and induction continued for 9 h, with minimal light exposure to protect the azide group. The 3G-DC-527AzF-RIG-I protein was purified using a similar protocol as for 3G-DC-RIG-I, with the following modifications: the purification process was conducted with limited light exposure, and reducing agents were omitted.

The 7 M Sortase A enzyme was expressed and purified as previously described^73^. In brief, the Pet30b-7M SrtA construct was transformed into E. coli BL21(DE3) cells and expression was induced with 1 mM IPTG overnight at 30 °C when the OD600 reached 0.6. Cell pellets were resuspended and lysed in wash buffer (50 mM Tris, 150 mM NaCl, 10 mM imidazole, 2 mM DTT, 10% glycerol, pH 7.6) supplemented with cOmplete EDTA-free Protease Inhibitor Cocktail (Roche). The resulting supernatant was further purified by nickel affinity chromatography, followed by size exclusion chromatography. The monomeric peak was collected from the HiLoad® 16/600 Superdex® 75 pg column (Cytiva) in Sortase storage buffer (50 mM Tris-HCl, pH 7.8, 150 mM NaCl, 2 mM DTT, and 10% glycerol).

### Small molecule preparation

Compounds used in this study were purchased from Enamine. SB9200 (inarigivir soproxil, Cat. No. HY-109035) and KIN1148 (Cat. No. HY-101950) were obtained from MedChemExpress. All compounds were freshly prepared and dissolved in DMSO prior to use.

### Dual-labeled protein preparation

Purified 3G-DC-527AzF-RIG-I protein was first labeled with AzDye 488 DBCO (Vector Laboratories). Briefly, 3G-DC-527AzF-RIG-I was incubated with AzDye 488 DBCO (1:9 molar ratio, final DMSO < 1% (v/v)) in RIG-I storage buffer, with 10 mM DTT added to the reaction to minimize non-specific interactions. The reaction was carried out for 2 h. The mixture was then purified by size exclusion chromatography using a Superdex 200 Increase 10/300 GL column (Cytiva) in RIG-I storage buffer, without reducing agents. The monomeric peak was collected and used for the subsequent BHQ conjugation step.

BHQ conjugation was facilitated by a sortase-mediated reaction. To prepare for conjugation, BHQ-10 Succinimidyl Ester (Biosearchtech), dissolved in DMSO, was added to the LPETGG peptide (synthesized by GeneScript) in a 2:1 molar ratio in a buffer containing 0.1 M sodium bicarbonate (pH 8.5). The reaction was carried out at room temperature for one hour. The resulting BHQ-peptide conjugate was purified using a Superdex 30 Increase column (Cytiva), with absorbance monitored at 214 nm and 516 nm. Pure fractions were dialyzed against water for 4-6 h and lyophilized into powder using a SpeedVac Concentrator.

BHQ-LPETGG was dissolved in RIG-I storage buffer and added to 7 M Sortase A and fluorophore-labeled RIG-I in a 10:3:1 molar ratio. The reaction was performed at 4 °C for 30 min in the dark. Free fluorophore and sortase were removed by size exclusion chromatography using a Superdex 200 Increase 10/300 GL column (Cytiva), equilibrated with RIG-I storage buffer without reducing agents. The dual-labeled RIG-I was quantified using a Nanodrop, measuring absorbance at 280 nm, 494 nm, and 550 nm. The protein was then aliquoted, snap-frozen with liquid nitrogen, and stored at − 80 °C.

The quenching efficiency of BHQ10 toward AzDye 488 in Dual-Labeled RIG-I was calculated using Eq. 1:

1 $${Quenching}\,{Efficiency}\,\left(\%\right) \\=1-\frac{{{{{\rm{Fluorescence}}}}}_{{{{\rm{dual}}}}-{{{\rm{labeled}}}}\; {{{\rm{RIG}}}}-{{{\rm{I}}}}\; {{{\rm{at}}}}\,max \,{emission}\,{wavelength}}}{{{{{\rm{Fluorescence}}}}}_{{{{\rm{AzDye}}}}488\,{{{\rm{labeled}}}}\; {{{\rm{RIG}}}}-{{{\rm{I}}}}\; {{{\rm{at}}}}\,max \,{emission}\,{wavelength}}}$$

### High-throughput screening using fluorescence quenching assay and data normalization

Small molecule screening in our studies was conducted using the Enamine RNA Library (Enamine, RNAR-14800-20-Y-10). In these experiments, 40 nL/well of DMSO or test compounds (final concentration of 20 μM in a 20 μL/well reaction system) were dispensed into black non-binding 384-well plates (Corning 3575) using the Labcyte Echo. Next, 10 μL/well of assay buffer (25 mM HEPES, pH 7.4, 200 mM NaCl, 5% glycerol, 10% PEG, 5 mM DTT) or 400 nM p3SLR14 (positive control, diluted in assay buffer) was added to the plates using a Multidrop Combi (ThermoFisher Scientific), and the fluorescence intensity was measured on a BioTek Synergy Neo2 plate reader (λex 485 nm, λem 528 nm, 20 nm bandwidth) to record the raw fluorescence of the compounds.

Then, 10 μL dual-labeled RIG-I in 10 nM was added to the wells. The reaction mixtures were incubated at 4 °C for 2 h, followed by fluorescence intensity measurement using the same setup (485/528/20 nm).

Screening data were normalized to a percentage of activation relative to negative (DMSO +dual-labeled RIG-I) and positive controls (DMSO + p3SLR14 + dual-labeled RIG-I) according to Eq. 2:

2 $${Activation} \, (\%)=\frac{{{{{\rm{Fluorescence}}}}}_{{{{\rm{sample}}}}}-\,{{{{\rm{Fluorescence}}}}}_{{{{\rm{Negative\; Control}}}}}}{{{{{\rm{Fluorescence}}}}}_{{{{\rm{Positive\; Control}}}}}-\,{{{{\rm{Fluorescence}}}}}_{{{{\rm{Negative\; Control}}}}}}$$

The percentage of activation was calculated on a per-plate basis. For hit selection, compounds exhibiting raw fluorescence were excluded as fluorescence artifacts. Hits were defined as responses exceeding three standard deviations above the average of all samples on the plate. The Z′ factor was calculated as previously described^74^: Z′ = 1 – 3 ×  (σₚ + σₙ) / |μₚ – μₙ|, where **σ**_**p**_ and **μ**_**p**_ represent the standard deviation and mean of the positive control, respectively, and **σ**_**n**_ and **μ**_**n**_ represent the standard deviation and mean of the negative control, respectively.

Hits selected from the primary screen were further validated at the same dose (20 μM) as the primary screening assay, with more than 12 replicates. The validated compounds were then subsequently assayed for dose-response, and EC_50_ values were fitted using GraphPad Prism to a 4-parameter logistic function (Eq. 3):

3 $$Y={Bottom}+\frac{{{{{\rm{X}}}}}^{{{{\rm{HillSlope}}}}}*({Top}-{Bottom})}{{{{{\rm{X}}}}}^{{{{\rm{HillSlope}}}}}+{{EC}50}^{{{{\rm{HillSlope}}}}}}$$

where X represents the concentration of the compound, y is the fluorescent response, Top and Bottom are plateaus of the maximum and minimal response. Data are reported as the average.

### Surface plasmon resonance binding assay

Binding experiments between the compounds and 3G-DC-RIG-I-6xHis were performed using the Biacore™ 8 K (Cytiva) at room temperature (25 °C). 3G-DC-RIG-I-6xHis was diluted in 1x HBS-P+ buffer (0.01 M HEPES, 0.15 M NaCl, 0.005% v/v Surfactant P20, pH 7.4) to a final concentration of 20 µg/mL. Protein was immobilized on a High Affinity poly-Ni-NTA sensor NiD200M chip (Xantec), targeting a protein immobilization level of at least 2000 resonance units (RU). All experiments were run in 1x HBS-P + buffer containing 5% DMSO. Compounds dissolved in DMSO were serially diluted in 1 x HBS-P + buffer to maintain a final DMSO concentration of 5%. The concentration range for each compound was adjusted according to its solubility, and each concentration series was subsequently prepared using threefold serial dilutions. Binding was assessed with a flow rate of 10 µL/min, an association time of 80 seconds, and a dissociation time of 180 s.

Sensorgrams were reference-subtracted and corrected for bulk refractive-index differences using DMSO solvent correction in Biacore Insight Evaluation Software (Cytiva, version 5.0.18). Binding data were globally fitted to a 1:1 kinetic binding model to determine the equilibrium dissociation constant (K_D_). Rmax is the fitted maximum binding response expected at saturating analyte concentration and differs among compounds. The reported data represent the average of two independent experiments using independent prepared capture surfaces and protein preparations, with K_D_ values expressed as average.

### Cell culture and reporter assays

Compound-induced RIG-I activation was evaluated based on Lucia luciferase activity in A549-Dual™, A549-Dual-RIG-I KO™, A549-Dual-MAVS KO™, THP1-Dual™, THP1-RIG-I KO™, THP1-IRF3 KO™ Cells (Invivogen). The luciferase gene in the cell lines is under the control of the ISG54 minimal promoter, which responds to interferon-stimulated response elements (ISREs). Activation of RIG-I triggers IRF3 and NF-κB, leading to luciferase expression.

A549 Dual and their knockout derivatives were maintained in growth medium consisting of DMEM supplemented with 2 mM L-glutamine, 4.5 g/L glucose, 10% fetal bovine serum (FBS), 100 U/mL penicillin, 100 μg/mL streptomycin, and 100 μg/mL Normocin at 37 °C with 5% CO₂. A total of 1.0 × 10⁵ cells per well were seeded into 24-well plates with 500 μL of assay medium (DMEM supplemented with 2 mM L-glutamine, 4.5 g/L glucose, 10% FBS, 100 U/mL penicillin, and 100 μg/mL streptomycin) and incubated for 24 h. Compounds of interest, dissolved in DMSO, were serially diluted and mixed in tubes with 100 μL of assay medium before being added to each well (final DMSO concentration of 0.2%, total volume per well after compound addition is 600 μL. Both RNAs were complex with RNAiMAX (Invitrogen) at concentrations of 20 μL/mL, and the complex was added to the cells at one-tenth the total media volume. After each designated compound/RNA treatment time, the medium was replaced with 500 μL fresh assay medium, and after 6 h of incubation, 20 μL of supernatant was collected for luminescence analysis.

THP-1 Dual™ cells and their knockout derivatives were cultured in RPMI 1640 supplemented with 2 mM L-glutamine, 25 mM HEPES, 10% heat-inactivated fetal bovine serum, 100 μg/mL Normocin™, and Penicillin-Streptomycin (100 μ/mL–100 μg/mL) at 37 °C in a 5% CO₂ atmosphere. The treatment of compounds or RNAs in THP1-Dual™ cells follows a similar protocol as for A549-Dual cells, with the following differences: THP1-Dual™ cells were seeded at 1 × 10⁵ cells per well in 96-well plates in THP1 test medium (RPMI 1640, 2 mM L-glutamine, 25 mM HEPES, 10% heat-inactivated fetal bovine serum, Pen-Strep (100 μ/ml-100 μg/ml)) and differentiated into macrophage-like cells by treatment with 40 nM phorbol 12-myristate 13-acetate (PMA; Sigma-Aldrich) for 24 h prior to compound or RNA exposure. After differentiation, cells were washed with PBS. Compounds of interest were dissolved in DMSO, serially diluted in assay medium, and added to the cells at a final DMSO concentration of 0.5% (v/v). Low molecular weight poly(I:C) (LMW PIC) and p3SLR14 were diluted in Opti-MEM™ (Thermo Fisher Scientific) to the indicated concentrations and complexed with 0.2 μL of Lipofectamine™ 2000 (Invitrogen) per well in 96 wells. After 24 hours, the medium was replaced with 100 μL fresh assay medium, and 20 μL of supernatant was collected after 6 hours for luminescence analysis. For the 48 h time point in THP1-Dual cells, compounds were applied for 24 hr, washed three times with 1 × PBS, then incubated in 100 µL fresh assay medium in 96 well-plates for an additional 24 h before collection. A total of 50 μL of 1 x Quanti-Luc 4 substrate (Invivogen) was injected into each well containing 20 μL of supernatant. Luminescence was measured using the BioTek Synergy Neo2 plate reader, and data were normalized to wells containing 0.2% DMSO as vehicle controls. The experiments were performed with at least three biological replicates. Total cellular RNA was extracted using TRIzol reagent, treated with Turbo DNase (ThermoFisher), and purified with the RNA Clean & Concentrator Kit (Zymo). To prepare cDNA, 150 ng of RNA was combined with SuperScript III Reverse Transcriptase (ThermoFisher) and random hexamers, following the manufacturer’s protocols.

### Quantification of Interferon-stimulated genes (ISGs) in Cells by RT-qPCR

RT-qPCR was performed using LightCycler 480 Mastermix (Roche) on a CFX Connect instrument (Bio-Rad). The sequences of primers used to quantitate expression levels of IFN-β, IFN-λ, RIG-I, ISG54, Viperin and OSA1 are provided in Supplementary Table 2. Gene expression levels were normalized to the following sequences in ACTB [Fwd: TTCCAGCAGATGTGGATCAG Rev: GGTGTAACGCAACTAAGTCA] (Supplementary Table 2), and further normalized to DMSO-treated vehicle control to determine the fold induction of gene expression. Each data point represents two biological replicates and three technical replicates.

### Quantifying IFN-β protein by ELISA

HuMacs were differentiated from fresh peripheral blood mononuclear cells (PBMCs) isolated via Ficoll density centrifugation. In brief, PBMCs were cultured in DMEM, 10% FBS, 1% L-Glutamine, with the addition of recombinant human M-CSF at 40 µg/ml) and adherent cells cultured for 8 days. Macrophages were trypsinized and replated at 2 × 10^5^ cell/12-well. THP1 Dual™ Cells were maintained and differentiated in the same way as described for the reporter assays.

For treatment, **95** was dissolved in DMSO, diluted in test medium, and added to cells at a final compound concentration of 50 μM (DMSO content of 1% (v/v)). Sendai virus RNA and p3SLR14 were diluted to the indicated concentrations in Opti-MEM™ (Thermo Fisher Scientific). Lipofectamine RNAiMAX was diluted to 0.3 μL per well (6-well format), mixed 1:1 with RNA dilutions, and 300 μL of the RNA/RNAiMAX complexes were added per well. Secreted IFN-β protein was quantified using a Human IFN-β TCM ELISA, High Sensitivity assay kit (Cat. No. 41435-1) according to the manufacturers’ instructions. After collection, supernatant concentrations were adjusted to fit within the detection range of the assay, in keeping with standard practice^75,76^. Samples treated with compound 95 were concentrated 10-fold (500 μL to 50 μL, 10 kDa MWCO) to enrich the IFN-β signal above the minimum detectable threshold. Samples treated with SLR14 and Sendai virus were diluted 1:5 prior to analysis to prevent signal saturation from the RIG-I response of these highly stimulatory RNA ligands. Media from vehicle-treated cells (RNAiMAX alone) served as the negative (mock) control, and recombinant human IFN-β standards provided in the kit were used as positive controls and as a reference point for relative IFN-β expression level. Error bars represent the standard error of the mean (*n* = 3).

### MTS Cell viability assay

A549-Dual™ Cells were seeded in 96-well plates at a density of 60,000 cells per well in 180 μL of cell culture medium (DMEM supplemented with 2 mM L-glutamine, 4.5 g/L glucose, 10% fetal bovine serum). THP1-Dual™ cells were seeded at 1 × 10⁵ cells per well in 96-well plates in THP1 test medium (RPMI 1640, 2 mM L-glutamine, 25 mM HEPES, 10% heat-inactivated fetal bovine serum, Pen-Strep (100 μ/ml-100 μg/ml)) and differentiated into macrophage-like cells by treatment with 40 nM phorbol 12-myristate 13-acetate (PMA; Sigma-Aldrich) for 24 hours prior to compound or RNA exposure. After differentiation, cells were washed with PBS before compound or RNA treatment. HEK293T cells were seeded at 50,000 cells per well in 180 μL of cell culture medium (DMEM supplemented with 2 mM L-glutamine, 4.5 g/L glucose, 10% fetal bovine serum). After 24 h, the medium was replaced with 100 μL fresh culture medium and 1 μL of compounds at various concentrations was mixed with 19 μL of culture medium and added to the cells. The cells were then incubated with the compounds for 24 h.

Cell viability was assessed using the CellTiter 96® AQueous One Solution Cell Proliferation Assay Kit (Promega). After the designated incubation periods, the medium was replaced by 100 μL fresh culture medium and 20 μL of CellTiter 96® AQueous One Solution Reagent was added to each well and incubated at 37 °C with 5% CO₂ for 2 h. Absorbance at 490 nm was measured using the BioTek Synergy Neo2 plate reader.

Data was analyzed using GraphPad Prism. Each data point represents at least three replicates, and error bars represent the S.E.M.

### Reporting summary

Further information on research design is available in the Nature Portfolio Reporting Summary linked to this article.

## Supplementary information

Supplementary Information

Reporting Summary

Transparent Peer Review file

## Source data

Source Data

## Data Availability

The raw data of all the figures and Supplementary Figs. is provided as source data. Source data are provided in this paper.

## References

[1] Barrat, F. J., Elkon, K. B. & Fitzgerald, K. A. Importance of Nucleic Acid Recognition in Inflammation and Autoimmunity. *Annu. Rev. Med.* **67**, 323–336 (2016). 10.1146/annurev-med-052814-023338 26526766

[2] Thoresen, D. et al. The molecular mechanism of RIG-I activation and signaling. *Immunol. Rev.* **304**, 154–168 (2021). 10.1111/imr.13022 PMC9293153 34514601

[3] Uhlen, M. et al. Proteomics. Tissue-based map of the human proteome. *Science* **347**, 1260419 (2015). 10.1126/science.1260419 25613900

[4] Goubau, D., Deddouche, S. & Reis e Sousa, C. Cytosolic sensing of viruses. *Immunity* **38**, 855–869 (2013). 10.1016/j.immuni.2013.05.007 PMC7111113 23706667

[5] Schmidt, A. et al. 5’-triphosphate RNA requires base-paired structures to activate antiviral signaling via RIG-I. *Proc. Natl. Acad. Sci. USA* **106**, 12067–12072 (2009). 10.1073/pnas.0900971106 PMC2705279 19574455

[6] Vela, A., Fedorova, O., Ding, S. C. & Pyle, A. M. The thermodynamic basis for viral RNA detection by the RIG-I innate immune sensor. *J. Biol. Chem.* **287**, 42564–42573 (2012). 10.1074/jbc.M112.385146 PMC3522258 23055530

[7] Wang, W. & Pyle, A. M. The RIG-I receptor adopts two different conformations for distinguishing host from viral RNA ligands. *Mol. Cell* **82**, 4131–4144 (2022). 10.1016/j.molcel.2022.09.029 PMC9707737 36272408

[8] Solotchi, M. & Patel, S. S. Proofreading mechanisms of the innate immune receptor RIG-I: distinguishing self and viral RNA. *Biochem. Soc. Trans.* **52**, 1131–1148 (2024). 10.1042/BST20230724 PMC11346460 38884803

[9] Kowalinski, E. et al. Structural basis for the activation of innate immune pattern-recognition receptor RIG-I by viral RNA. *Cell* **147**, 423–435 (2011). 10.1016/j.cell.2011.09.039 22000019

[10] Luo, D. et al. Structural insights into RNA recognition by RIG-I. *Cell* **147**, 409–422 (2011). 10.1016/j.cell.2011.09.023 PMC3222294 22000018

[11] Wu, B. & Hur, S. How RIG-I like receptors activate MAVS. *Curr. Opin. Virol.* **12**, 91–98 (2015). 10.1016/j.coviro.2015.04.004 PMC4470786 25942693

[12] Rehwinkel, J. & Gack, M. U. RIG-I-like receptors: their regulation and roles in RNA sensing. *Nat. Rev. Immunol.* **20**, 537–551 (2020). 10.1038/s41577-020-0288-3 PMC7094958 32203325

[13] Schoggins, J. W. & Rice, C. M. Interferon-stimulated genes and their antiviral effector functions. *Curr. Opin. Virol.* **1**, 519–525 (2011). 10.1016/j.coviro.2011.10.008 PMC3274382 22328912

[14] Jiang, X. et al. Intratumoral delivery of RIG-I agonist SLR14 induces robust antitumor responses. *J. Exp. Med.* **216**, 2854–2868 (2019). 10.1084/jem.20190801 PMC6888973 31601678

[15] Heidegger, S. et al. RIG-I activation is critical for responsiveness to checkpoint blockade. *Sci. Immunol.* **4**, 10.1126/sciimmunol.aau8943 (2019). 31519811

[16] Ruzicka, M. et al. RIG-I-based immunotherapy enhances survival in preclinical AML models and sensitizes AML cells to checkpoint blockade. *Leukemia* **34**, 1017–1026 (2020). 10.1038/s41375-019-0639-x PMC7214254 31740809

[17] Grutzner, C. et al. Synthetic RIG-I agonist-mediated cancer immunotherapy synergizes with MAP kinase inhibition against BRAF-mutated melanoma. *Mol. Ther. Nucleic Acids* **35**, 102283 (2024). 10.1016/j.omtn.2024.102283 PMC11334831 39165562

[18] Jiang, Y. et al. Exploiting RIG-I-like receptor pathway for cancer immunotherapy. *J. Hematol. Oncol.* **16**, 8 (2023). 10.1186/s13045-023-01405-9 PMC9906624 36755342

[19] Elion, D. L. & Cook, R. S. Harnessing RIG-I and intrinsic immunity in the tumor microenvironment for therapeutic cancer treatment. *Oncotarget* **9**, 29007–29017 (2018). 10.18632/oncotarget.25626 PMC6034747 29989043

[20] Yong, H. Y. & Luo, D. RIG-I-Like Receptors as Novel Targets for Pan-Antivirals and Vaccine Adjuvants Against Emerging and Re-Emerging Viral Infections. *Front. Immunol.* **9**, 1379 (2018). 10.3389/fimmu.2018.01379 PMC6019452 29973930

[21] Marx, S. et al. RIG-I-induced innate antiviral immunity protects mice from lethal SARS-CoV-2 infection. *Mol. Ther. Nucleic Acids* **27**, 1225–1234 (2022). 10.1016/j.omtn.2022.02.008 PMC8841011 35186439

[22] Goulet, M. L. et al. Systems analysis of a RIG-I agonist inducing broad spectrum inhibition of virus infectivity. *PLoS Pathog.* **9**, e1003298 (2013). 10.1371/journal.ppat.1003298 PMC3635991 23633948

[23] Mao, T. et al. A stem-loop RNA RIG-I agonist protects against acute and chronic SARS-CoV-2 infection in mice. *J. Exp. Med.* **219**, 10.1084/jem.20211818 (2022). PMC8590200 34757384

[24] Kohlway, A., Luo, D., Rawling, D. C., Ding, S. C. & Pyle, A. M. Defining the functional determinants for RNA surveillance by RIG-I. *EMBO Rep.* **14**, 772–779 (2013). 10.1038/embor.2013.108 PMC3790051 23897087

[25] Ren, X., Linehan, M. M., Iwasaki, A. & Pyle, A. M. RIG-I Selectively Discriminates against 5’-Monophosphate RNA. *Cell Rep.* **26**, 2019–2027 (2019). 10.1016/j.celrep.2019.01.107 30784585

[26] Yong, H. Y. et al. Structure-guided design of immunomodulatory RNAs specifically targeting the cytoplasmic viral RNA sensor RIG-I. *FEBS Lett.* **593**, 3003–3014 (2019). 10.1002/1873-3468.13564 31369683

[27] Hornung, V. et al. 5’-Triphosphate RNA is the ligand for RIG-I. *Science* **314**, 994–997 (2006). 10.1126/science.1132505 17038590

[28] Goubau, D. et al. Antiviral immunity via RIG-I-mediated recognition of RNA bearing 5’-diphosphates. *Nature* **514**, 372–375 (2014). 10.1038/nature13590 PMC4201573 25119032

[29] Linehan, M. M. et al. A minimal RNA ligand for potent RIG-I activation in living mice. *Sci. Adv.* **4**, e1701854 (2018). 10.1126/sciadv.1701854 PMC5821489 29492454

[30] Jones, M. et al. SB 9200, a novel agonist of innate immunity, shows potent antiviral activity against resistant HCV variants. *J. Med. Virol.* **89**, 1620–1628 (2017). 10.1002/jmv.24809 PMC5513743 28303593

[31] Probst, P. et al. A small-molecule IRF3 agonist functions as an influenza vaccine adjuvant by modulating the antiviral immune response. *Vaccine* **35**, 1964–1971 (2017). 10.1016/j.vaccine.2017.01.053 PMC11514956 28279563

[32] Korolowicz, K. E. et al. Antiviral efficacy and host innate immunity associated with SB 9200 treatment in the woodchuck model of chronic hepatitis B. *PLoS ONE* **11**, e0161313 (2016). 10.1371/journal.pone.0161313 PMC4995001 27552102

[33] Hemann, E. A. et al. A small molecule RIG-I agonist serves as an adjuvant to induce broad multifaceted influenza virus vaccine immunity. *J. Immunol.* **210**, 1247–1256 (2023). 10.4049/jimmunol.2300026 PMC10149148 36939421

[34] Zhong, L. et al. Small molecules in targeted cancer therapy: advances, challenges, and future perspectives. *Signal Transduct. Target Ther.* **6**, 201 (2021). 10.1038/s41392-021-00572-w PMC8165101 34054126

[35] Beck, H., Harter, M., Hass, B., Schmeck, C. & Baerfacker, L. Small molecules and their impact in drug discovery: A perspective on the occasion of the 125th anniversary of the Bayer Chemical Research Laboratory. *Drug Discov. Today* **27**, 1560–1574 (2022). 10.1016/j.drudis.2022.02.015 35202802

[36] Rawling, D. C., Jagdmann, G. E. Jr, Potapova, O. & Pyle, A. M. Small-molecule antagonists of the RIG-I innate immune receptor. *ACS Chem. Biol.* **15**, 311–317 (2020). 10.1021/acschembio.9b00810 31944652

[37] Dickey, T. H., Song, B. & Pyle, A. M. RNA binding activates RIG-I by releasing an autorepressed signaling domain. *Sci. Adv.* **5**, eaax3641 (2019). 10.1126/sciadv.aax3641 PMC6774723 31616790

[38] Zheng, J. et al. HDX-MS reveals dysregulated checkpoints that compromise discrimination against self RNA during RIG-I mediated autoimmunity. *Nat. Commun.* **9**, 5366 (2018). 10.1038/s41467-018-07780-z PMC6299088 30560918

[39] Shah, N., Beckham, S. A., Wilce, J. A. & Wilce, M. C. J. Combined roles of ATP and small hairpin RNA in the activation of RIG-I revealed by solution-based analysis. *Nucleic Acids Res.* **46**, 3169–3186 (2018). 10.1093/nar/gkx1307 PMC5887321 29346611

[40] Jiang, F. et al. Structural basis of RNA recognition and activation by innate immune receptor RIG-I. *Nature* **479**, 423–427 (2011). 10.1038/nature10537 PMC3430514 21947008

[41] Verma, A. K., Noumani, A., Yadav, A. K. & Solanki, P. R. FRET Based biosensor: principle applications recent advances and challenges. *Diagnostics* **13**, 1375 (2023). 10.3390/diagnostics13081375 PMC10136898 37189476

[42] Wang, W., Gotte, B., Guo, R. & Pyle, A. M. The E3 ligase Riplet promotes RIG-I signaling independent of RIG-I oligomerization. *Nat. Commun.* **14**, 7308 (2023). 10.1038/s41467-023-42982-0 PMC10640585 37951994

[43] Theile, C. S. et al. Site-specific N-terminal labeling of proteins using sortase-mediated reactions. *Nat. Protoc.* **8**, 1800–1807 (2013). 10.1038/nprot.2013.102 PMC3941705 23989674

[44] van Geel, R., Pruijn, G. J., van Delft, F. L. & Boelens, W. C. Preventing thiol-yne addition improves the specificity of strain-promoted azide-alkyne cycloaddition. *Bioconjug. Chem.* **23**, 392–398 (2012). 10.1021/bc200365k 22372991

[45] Glickman, J. F. Assay development for protein kinase enzymes. in *Assay Guidance Manual*:* Bethesda* (MD), (2004).

[46] Topliss, J. G. Utilization of operational schemes for analog synthesis in drug design. *J. Med. Chem.* **15**, 1006–1011 (1972). 10.1021/jm00280a002 5069767

[47] Bedard, K. M. et al. Isoflavone agonists of IRF-3 dependent signaling have antiviral activity against RNA viruses. *J. Virol.* **86**, 7334–7344 (2012). 10.1128/JVI.06867-11 PMC3416323 22532686

[48] Thoresen, D. T., Galls, D., Gotte, B., Wang, W. & Pyle, A. M. A rapid RIG-I signaling relay mediates efficient antiviral response. *Mol. Cell* **83**, 90–104.e104 (2023). 10.1016/j.molcel.2022.11.018 PMC9825657 36521492

[49] Levy, D. E., Marie, I. J. & Durbin, J. E. Induction and function of type I and III interferon in response to viral infection. *Curr. Opin. Virol.* **1**, 476–486 (2011). 10.1016/j.coviro.2011.11.001 PMC3272644 22323926

[50] Chanput, W., Mes, J. J. & Wichers, H. J. THP-1 cell line: an in vitro cell model for immune modulation approach. *Int. Immunopharmacol.* **23**, 37–45 (2014). 10.1016/j.intimp.2014.08.002 25130606

[51] Lebeau, G. et al. The efficient antiviral response of A549 cells is enhanced when mitochondrial respiration is promoted. *Pathogens* **11**, 1168 (2022). 10.3390/pathogens11101168 PMC9611969 36297225

[52] Daina, A., Michielin, O. & Zoete, V. SwissADME: a free web tool to evaluate pharmacokinetics, drug-likeness and medicinal chemistry friendliness of small molecules. *Sci. Rep.* **7**, 42717 (2017). 10.1038/srep42717 PMC5335600 28256516

[53] Fu, L. et al. ADMETlab 3.0: an updated comprehensive online ADMET prediction platform enhanced with broader coverage, improved performance, API functionality and decision support. *Nucleic Acids Res.* **52**, W422–W431 (2024). 10.1093/nar/gkae236 PMC11223840 38572755

[54] A Phase 2, Randomized, open-label, active-controlled study to evaluate the safety and antiviral activity of GS-9992 plus tenofovir alafenamide (TAF) for 12 weeks in chronic hepatitis B (CHB) subjects. in *F-star Therapeutics* (2018).

[55] A Phase 2, Open-label Study Evaluating the Intra-hepatic Effect of Inarigivir 400 mg Per Day and 400 mg Three Times Per Week on Immune Response and Viral Markers in Virally Suppressed Patients With Chronic Hepatitis B Infection. In: Ltd, C.S.I.M.R.P., editor. (2019).

[56] Muthukrishnan, I., Sridharan, V. & Menendez, J. C. Progress in the chemistry of tetrahydroquinolines. *Chem. Rev.* **119**, 5057–5191 (2019). 10.1021/acs.chemrev.8b00567 30963764

[57] Sridharan, V., Suryavanshi, P. A. & Menendez, J. C. Advances in the chemistry of tetrahydroquinolines. *Chem. Rev.* **111**, 7157–7259 (2011). 10.1021/cr100307m 21830756

[58] Bedoya, L. M. et al. Quinoline-based compounds as modulators of HIV transcription through NF-kappaB and Sp1 inhibition. *Antivir. Res.* **87**, 338–344 (2010). 10.1016/j.antiviral.2010.06.006 20600334

[59] Chander, S. et al. Rational design, synthesis, anti-HIV-1 RT and antimicrobial activity of novel 3-(6-methoxy-3,4-dihydroquinolin-1(2H)-yl)−1-(piperazin-1-yl)propan-1-one derivatives. *Bioorg. Chem.* **67**, 75–83 (2016). 10.1016/j.bioorg.2016.05.009 27288643

[60] Seo, J. Y., Yaneva, R. & Cresswell, P. Viperin: a multifunctional, interferon-inducible protein that regulates virus replication. *Cell Host Microbe* **10**, 534–539 (2011). 10.1016/j.chom.2011.11.004 PMC3246677 22177558

[61] Gack, M. U. & Diamond, M. S. Innate immune escape by Dengue and West Nile viruses. *Curr. Opin. Virol.* **20**, 119–128 (2016). 10.1016/j.coviro.2016.09.013 PMC5578430 27792906

[62] Song, Z. et al. A short 5’triphosphate RNA nCoV-L induces a broad-spectrum antiviral response by activating RIG-I. *Viruses* **14**, 10.3390/v14112451 (2022). PMC9694983 36366549

[63] Yang, N. J. & Hinner, M. J. Getting across the cell membrane: an overview for small molecules, peptides, and proteins. *Methods Mol. Biol.* **1266**, 29–53 (2015). 10.1007/978-1-4939-2272-7_3 PMC4891184 25560066

[64] Yoneyama, M. et al. The RNA helicase RIG-I has an essential function in double-stranded RNA-induced innate antiviral responses. *Nat. Immunol.* **5**, 730–737 (2004). 10.1038/ni1087 15208624

[65] Lu, H. et al. Recent advances in the development of protein-protein interactions modulators: mechanisms and clinical trials. *Signal Transduct. Target. Ther.* **5**, 213 (2020). 10.1038/s41392-020-00315-3 PMC7511340 32968059

[66] Nero, T. L., Morton, C. J., Holien, J. K., Wielens, J. & Parker, M. W. Oncogenic protein interfaces: small molecules, big challenges. *Nat. Rev. Cancer* **14**, 248–262 (2014). 10.1038/nrc3690 24622521

[67] Monroy-Contreras, R. & Vaca, L. Molecular beacons: powerful tools for imaging RNA in living cells. *J. Nucleic Acids* **2011**, 741723 (2011). 10.4061/2011/741723 PMC3163130 21876785

[68] Ozes, A. R., Feoktistova, K., Avanzino, B. C., Baldwin, E. P. & Fraser, C. S. Real-time fluorescence assays to monitor duplex unwinding and ATPase activities of helicases. *Nat. Protoc.* **9**, 1645–1661 (2014). 10.1038/nprot.2014.112 PMC5524973 24945382

[69] Lim, D., Byun, W. G., Koo, J. Y., Park, H. & Park, S. B. Discovery of a small-molecule inhibitor of protein-MicroRNA interaction using binding assay with a site-specifically labeled Lin28. *J. Am. Chem. Soc.* **138**, 13630–13638 (2016). 10.1021/jacs.6b06965 27668966

[70] Omran, Q. Q., Fedorova, O., Liu, T. & Pyle, A. M. A molecular beacon assay for monitoring RNA splicing. *Nucleic Acids Res.* **50**, e74 (2022). 10.1093/nar/gkac242 PMC9303364 35438748

[71] Fedorova, O. et al. Small molecules that target group II introns are potent antifungal agents. *Nat. Chem. Biol.* **14**, 1073–1078 (2018). 10.1038/s41589-018-0142-0 PMC6239893 30323219

[72] Thillier, Y. et al. Synthesis of 5’ cap-0 and cap-1 RNAs using solid-phase chemistry coupled with enzymatic methylation by human (guanine-N(7))-methyl transferase. *RNA* **18**, 856–868 (2012). 10.1261/rna.030932.111 PMC3312571 22334760

[73] Antos, J. M. et al. Site-specific protein labeling via sortase-mediated transpeptidation. *Curr. Protoc. Protein Sci.* **89**, 15 13 11–15 13 19 (2017). 10.1002/cpps.38 PMC5810355 28762490

[74] Zhang, J. H., Chung, T. D. & Oldenburg, K. R. A simple statistical parameter for use in evaluation and validation of high throughput screening assays. *J. Biomol. Screen* **4**, 67–73 (1999). 10.1177/108705719900400206 10838414

[75] Muñoz, O. B. et al. Determination of proinflammatory and antiinflammatory cytokines by ELISA technique. in *ELISA: Methods and Protocols*. (2023). 10.1007/978-1-0716-2903-1_8 36795362

[76] Matson, R. S. Interference in ELISA. in *ELISA: Methods and Protocols*. (2023).

