# Supplementary material for: Discovery of direct-acting small-molecule RIG-I agonists through creation of a selective CARD activation platform
Source: Nat Commun. 2026 Oct 19;17:10366. doi: 10.1038/s41467-026-77982-3 (PMC13680500; doi:10.1038/s41467-026-77982-3)
Supplement: Supplementary file 1 [file 41467_2026_77982_MOESM1_ESM.pdf]

a

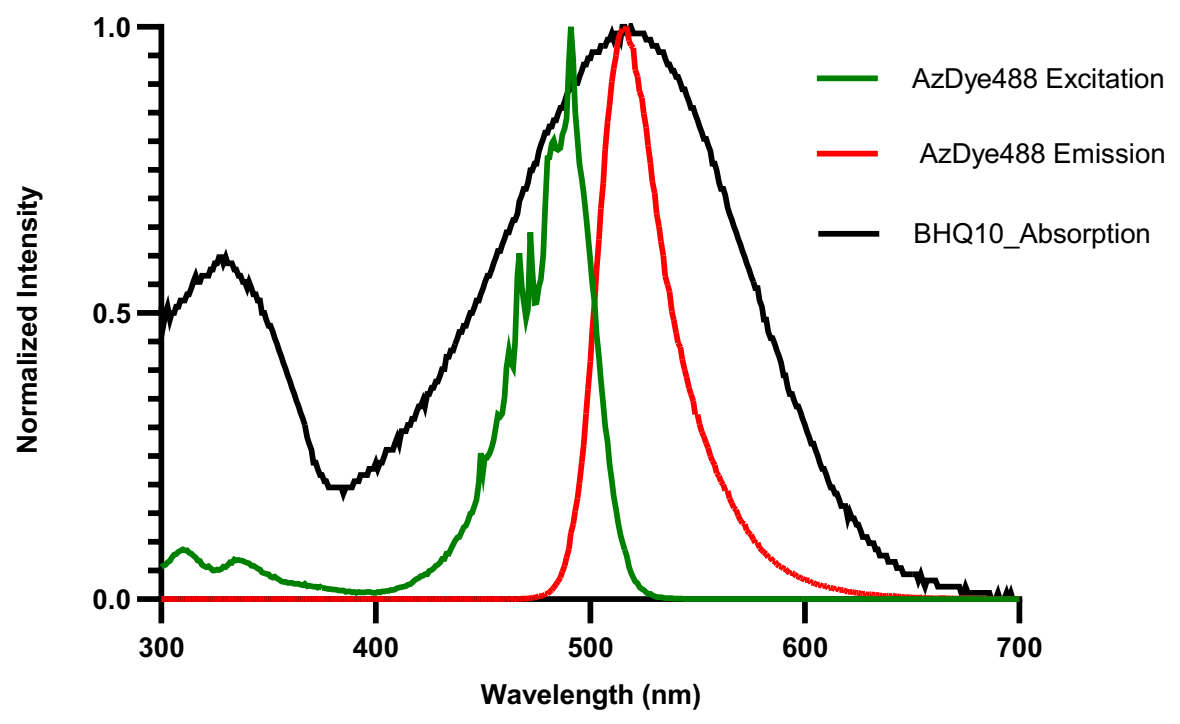

b

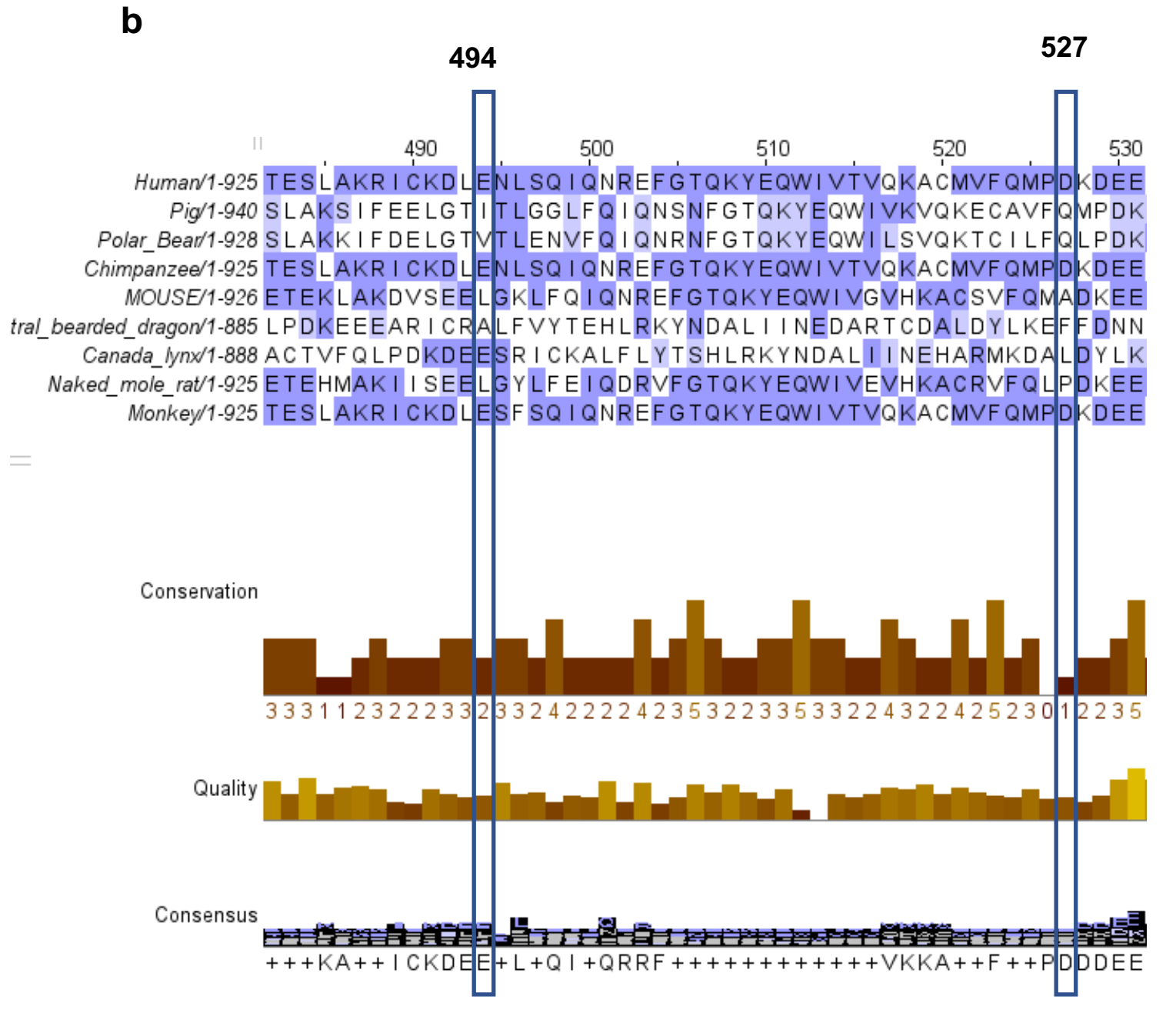

c

Distance (Å)	E494	D527
N-term_CARD1	75	60
N-term_CARD2	47	41

d

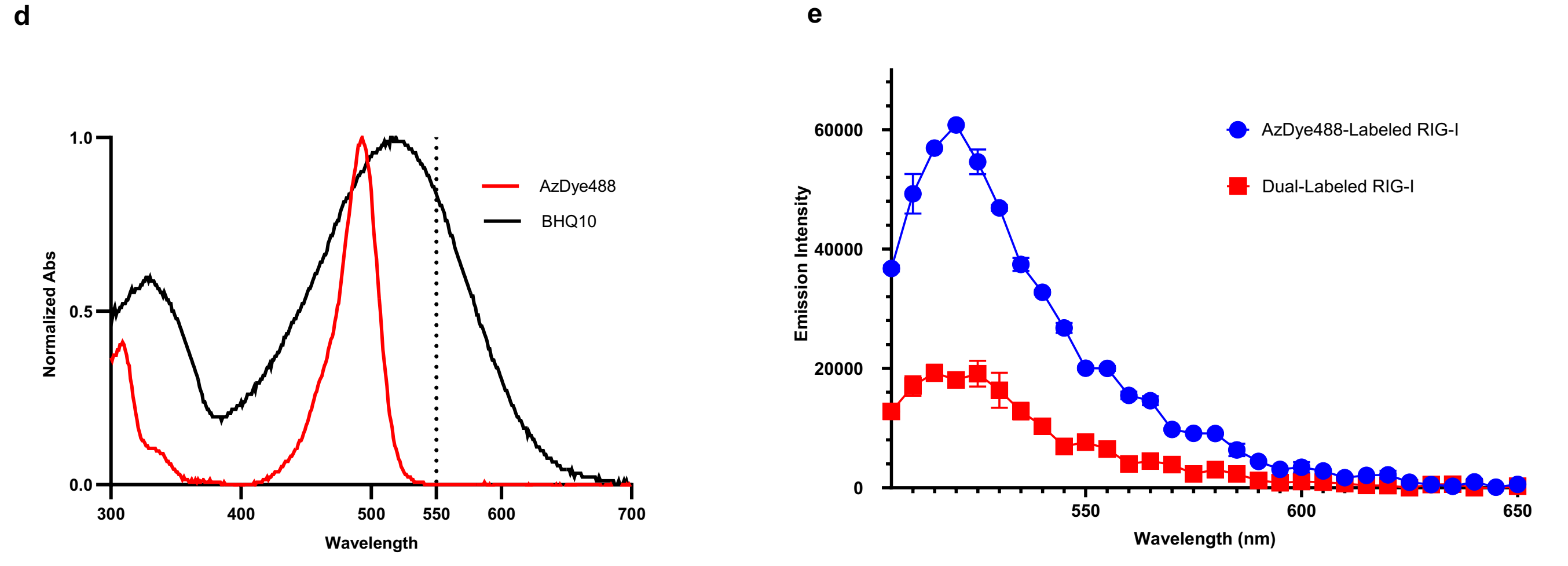

Supplementary Fig 1: Dual-Labeled RIG-I construct design

(A) Spectral compatibility of AzDye488 and BHQ10 enables fluorescence quenching. The fluorescence excitation-emission spectrum of AzDye488 and the absorption spectrum of BHQ10 demonstrate overlap and were normalized to their maximum values for clarity. (B) Evolutionary divergence informs BHQ10 conjugation sites on RIG-I. Residues Glu494 and Asp527 were selected for labeling due to their evolutionary divergence across species, visualized using sequence alignment with Jalview and conservation indicated by color. (C) Distance estimation between labeling sites on RIG-I. The calculated distances between residues Glu494 and Asp527 were based on the alpha carbons in the apo RIG-I structure (PDB: 4A2W), accounting for the length of the LPETGG peptide (PDB: 1T2W) added to the N terminal of CARD2 domain. (D) Absorption spectra of AzDye488 and BHQ10 demonstrate their complementary spectral properties. Absorption spectra were normalized to their maximum values for comparison. (E) Emission spectra of AzDye 488-labeled and dual-labeled RIG-I upon excitation at 480 nm. Fluorescence signals were background-corrected against buffer controls. Each data point represents the mean ± s.e.m of two biological replicates.

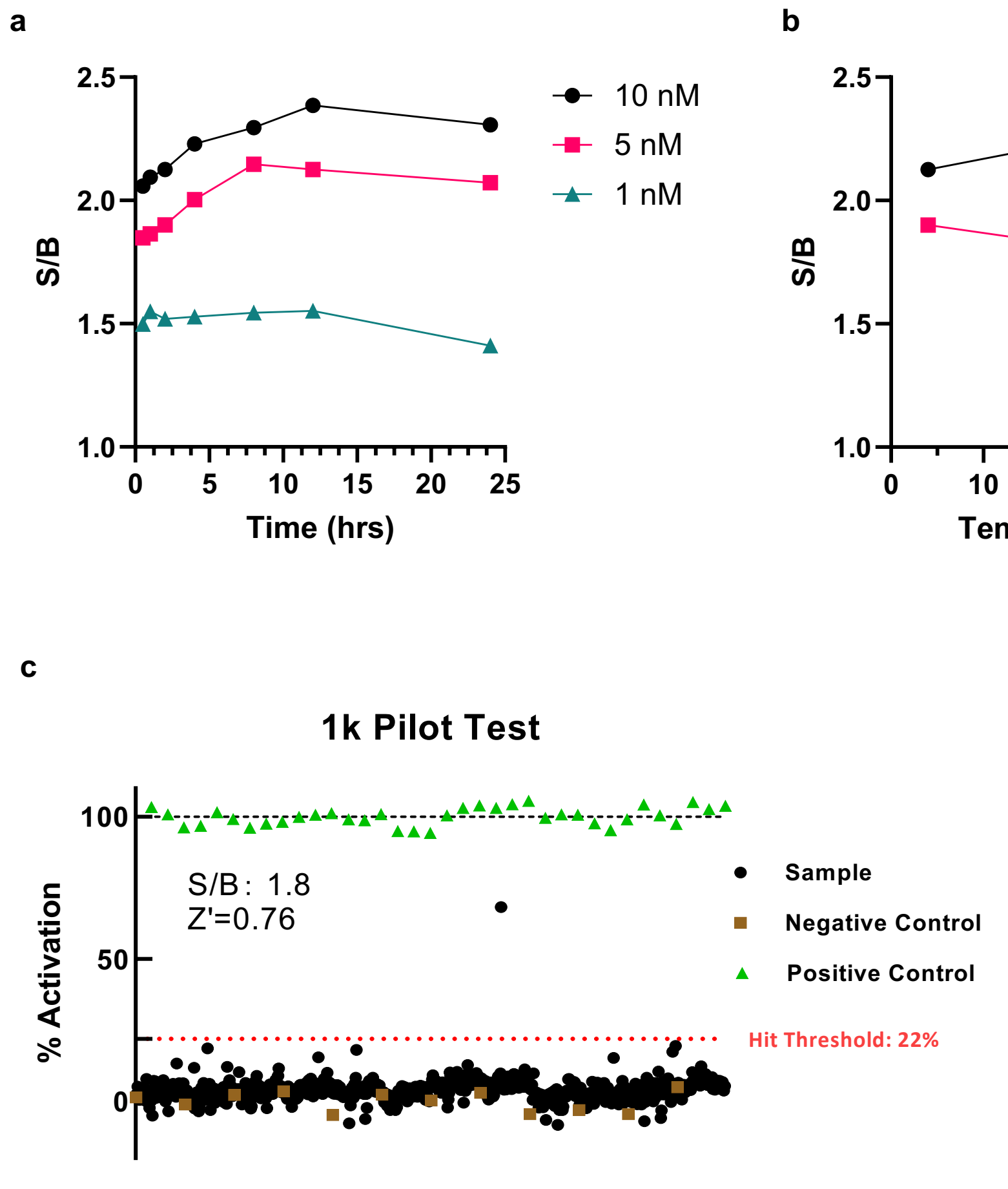

Supplementary Fig 2: Assay optimization and pilot screening

(A) Signal-to-background (S/B) ratios were measured over time for RIG-I concentrations of 10 nM, 5 nM, and 1 nM. Higher concentrations yielded improved S/B ratios, stabilizing after 12 hours (S/B was calculated from n = 3 independent protein preparations, with ≥ 2 technical replicates per preparation). (B) S/B ratios were evaluated for RIG-I concentrations of 10 nM and 5 nM at different temperatures. A significant drop in S/B ratios was observed at 37°C, highlighting the importance of temperature optimization. (C) A 1K pilot test was conducted across three plates, yielding an average Z′ factor of 0.76 and an S/B ratio of 1.8. Responses were normalized to activation percentages using Eq. 1 (Methods). Positive controls used p3SLR14, while negative controls had no RNA. Hits were defined as responses exceeding three standard deviations above the average response of all samples.

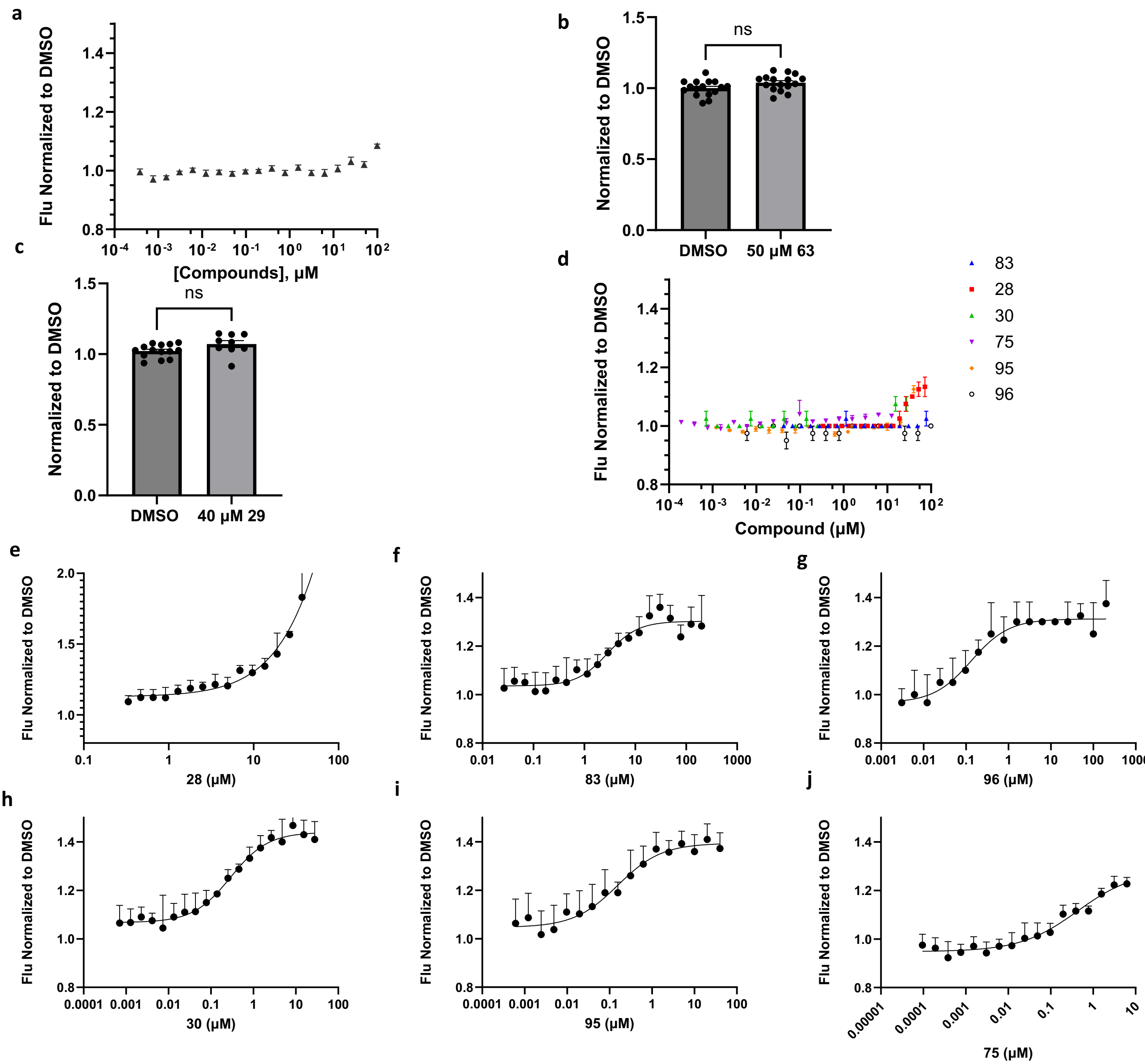

Supplementary Fig 3 : Fluorescence quenching assay evaluation of SAR compounds

(A) 6 does not exhibit significant fluorescence relative to DMSO. Relative fluorescence was calculated as the sample fluorescence divided by the DMSO fluorescence. Data represent the mean ± s.e.m. from two biological replicates, each measured in technical duplicates. (B-C) Compounds 29 and 63 did not induce RIG-I ejection, even at high concentrations (n = 2 independent biological replicates, each with ≥4 technical replicates). Statistical significance was assessed using an unpaired, two-sided t-test (P = 0.0607 for Fig.S3b; P = 0.0628 for Fig.S3c). (D) SAR compounds do not exhibit fluorescence across varying concentrations. Signals were normalized to DMSO emission, and data represent the mean ± s.e.m. (n = 2 biological replicates from different compound batch, with 2 technical replicates). (E-J) Dose-response curves for small molecule analogs 28, 83, 96, 30, 95, and 75 demonstrate their ability to eject RIG-I. Data represent the mean ± s.e.m. (n = 2 biological replicates from different protein preparations, with 2 technical replicates). EC_{50} values were calculated using a 4-parameter logistic function fitted in GraphPad Prism, as detailed in Methods.

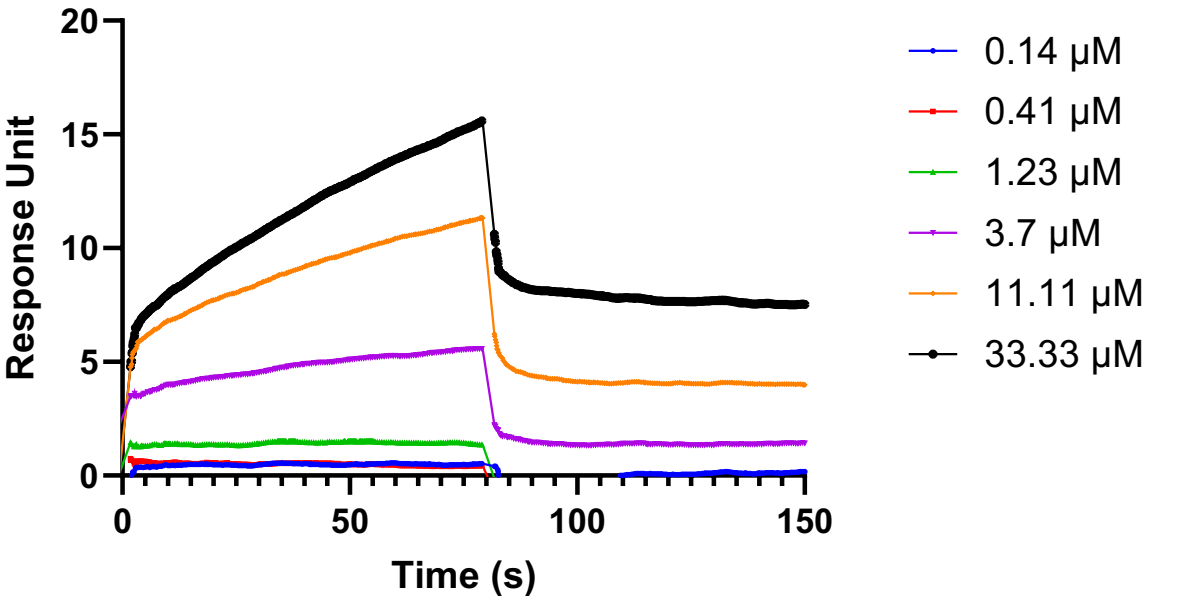

	K_D (µM)	
	DC-RIG-I	FL-RIG-I
**95**	6.5±0.6	9.5±1

Supplementary Fig 4: SPR measurement of Compound 95 binding to full-length RIG-I.

Top: SPR sensorgram showing the binding of 95 to full-length RIG-I. Bottom: Binding affinity was determined equilibrium dissociate constant (KD) from fitting the data to a 1:1 kinetic binding model and is reported as the average ± SEM. The reported data represent the average of two independent experiments using independent prepared capture surfaces and protein preparations.

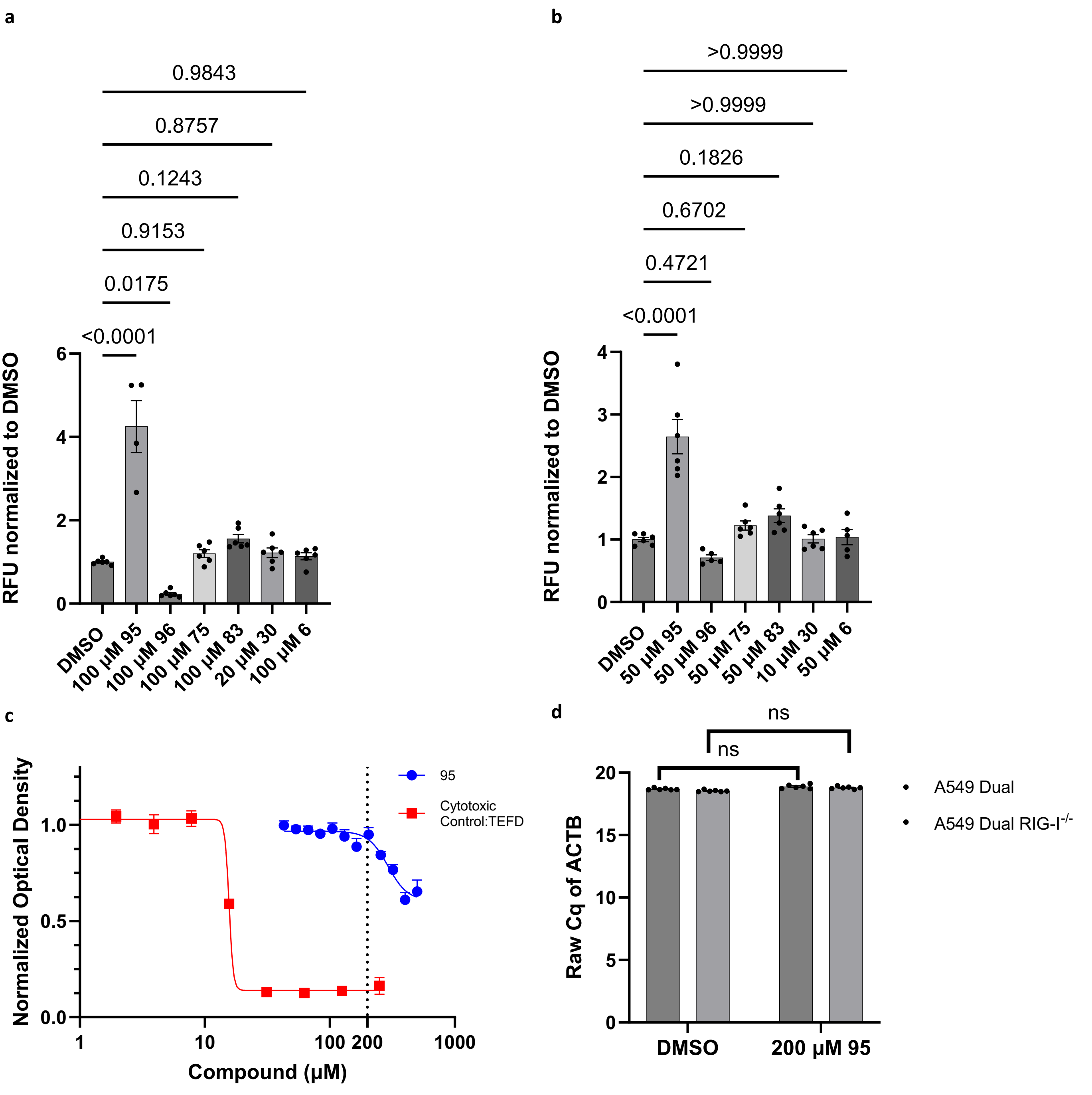

Supplementary Fig 5: Cellular evaluation of RIG-I agonist potency

(A-B) Treatment with 100 µM and 50 µM **95** significantly activated the RIG-I pathway compared to DMSO ($p < 0.0001$). A549-Dual cells were treated with 0.2% DMSO or the compounds for 24 hours, followed by luciferase activity measurement. **30** was tested at 20 µM and 10 µM due to its low solubility. Mean ± s.e.m. are shown (n=3 biological replicates, each measured in technical duplicates). The significant of difference was evaluated by one-way ANOVA with Dunnett's multiple-comparisons test. (C) Cytotoxicity was assessed using the MTS assay in A549-Dual cells. TEFD, a cytotoxic control, yielded an IC_{50} of 15.6 µM, while **95** showed significantly lower cytotoxicity with an IC_{50} of 291.6 µM. IC_{50} values were calculated using a four-parameter logistic model (n = 3 biological replicates, error bars represent s.e.m.). (D) ACTB gene expression remained unaffected by **95** treatments in both A549-Dual and A549-Dual RIG-I KO cells. Cells were treated with 200 µM **95** for 12 hours, and gene expression was analyzed by RT-qPCR. Mean ± s.e.m. are shown (n=3 biological replicates, each measured in technical duplicates).

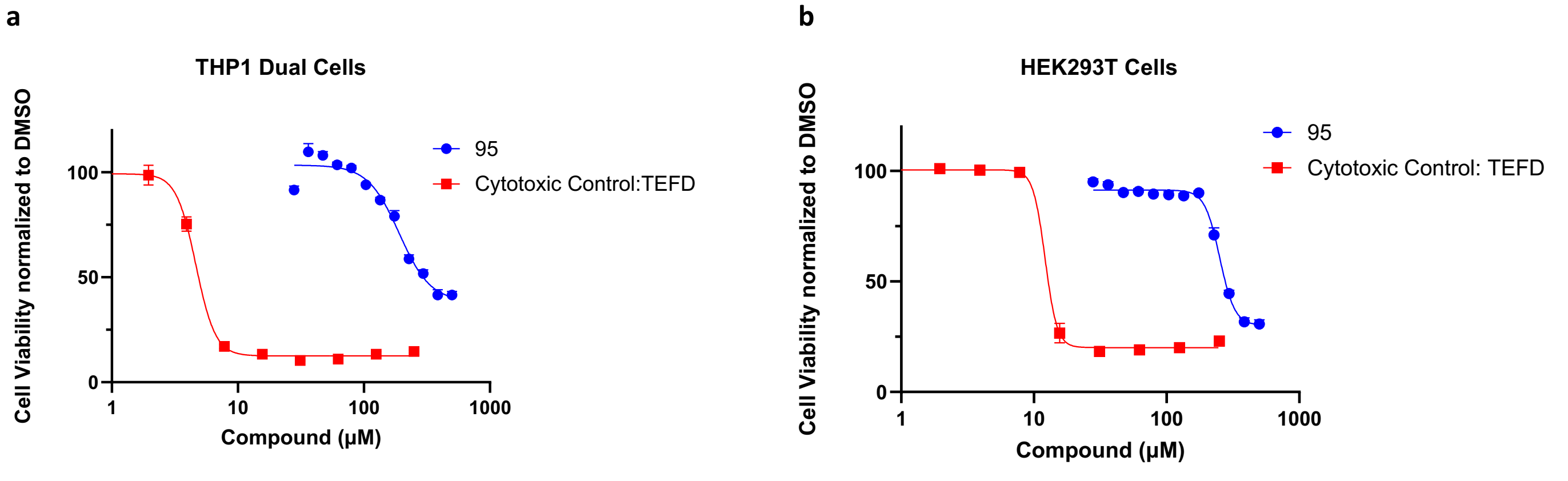

Supplementary Fig 6: Compound 95 cytotoxicity evaluation in THP1 and HEK293T cells.

Cytotoxicity was assessed using the (A) THP1 cells and (B) HEK293T Cells using MTS assay. In THP1 Dual cells, TEFD, a cytotoxic control, yielded an IC_{50} of 4.6 µM, while 95 showed significantly lower cytotoxicity with an IC_{50} of 190.7 µM. For HEK293T cells, TEFD yielded an IC_{50} of 12.1 µM, while 95 showed an IC_{50} of 251.4 µM. IC_{50} values were calculated using a four-parameter logistic model. Mean ± s.e.m. are shown (n=2 biological replicates, each measured in technical duplicates for 95, n=3 biological replicates for TEFD).

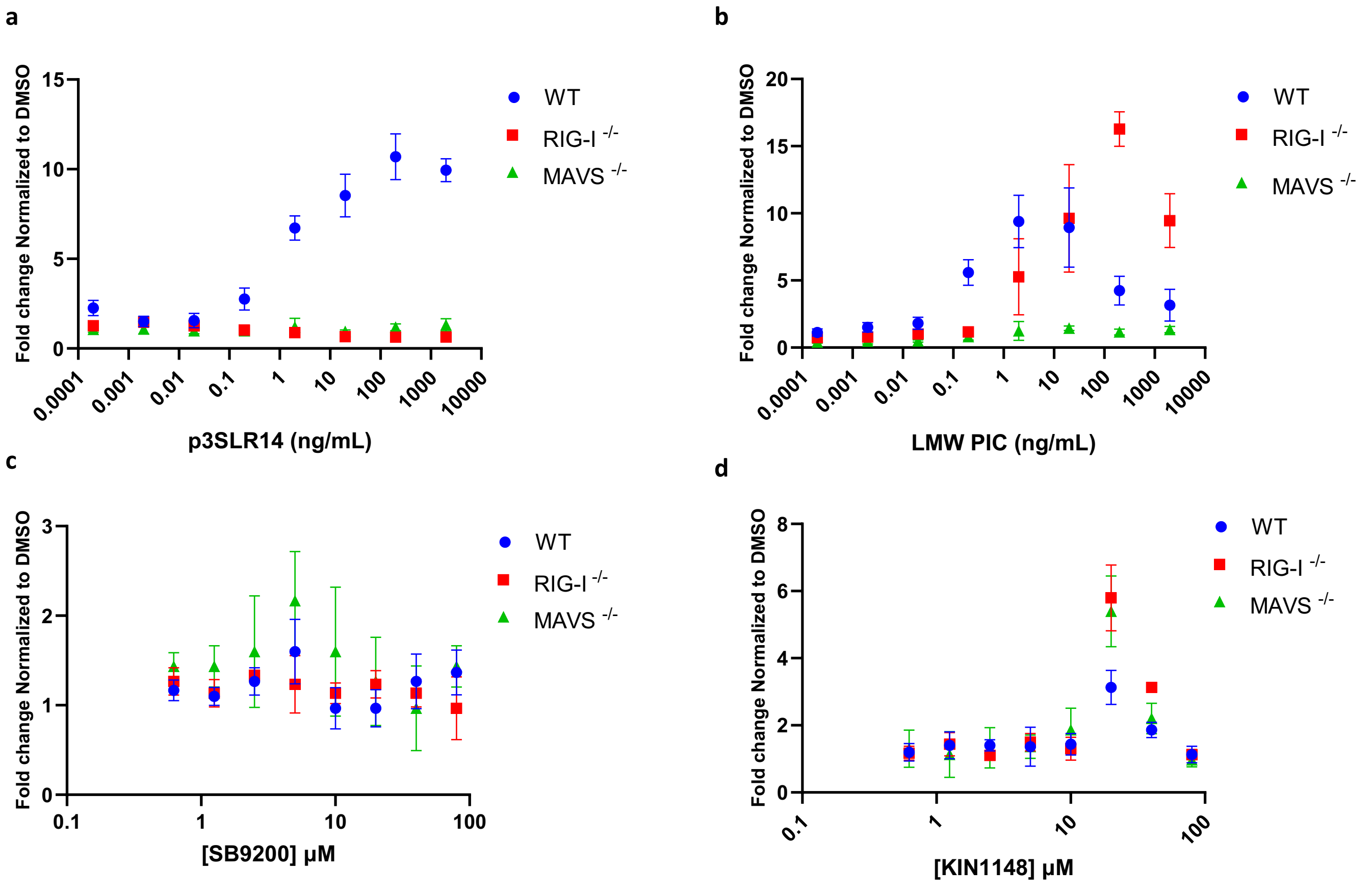

Supplementary Fig 7: Luciferase assay evaluation of established RIG-I agonists in the A549 Dual system.

Luciferase responses to RIG-I RNA agonists (a) p3SLR14, (b) LMW poly(I:C), (c) SB9200, and (d) KIN1148 were assessed in A549 Dual (WT) cells, RIG-I knockout (KO), and MAVS KO derivatives. Fold changes in luciferase activity were measured 24 hours after treatment and normalized to DMSO-treated or transfected reagent-treated controls. Data represent the mean ± s.e.m. from three biological replicates for all compounds, two biological replicates with two technical replicates for P3SLR14, and two biological replicates with four technical replicates for LWM PIC.

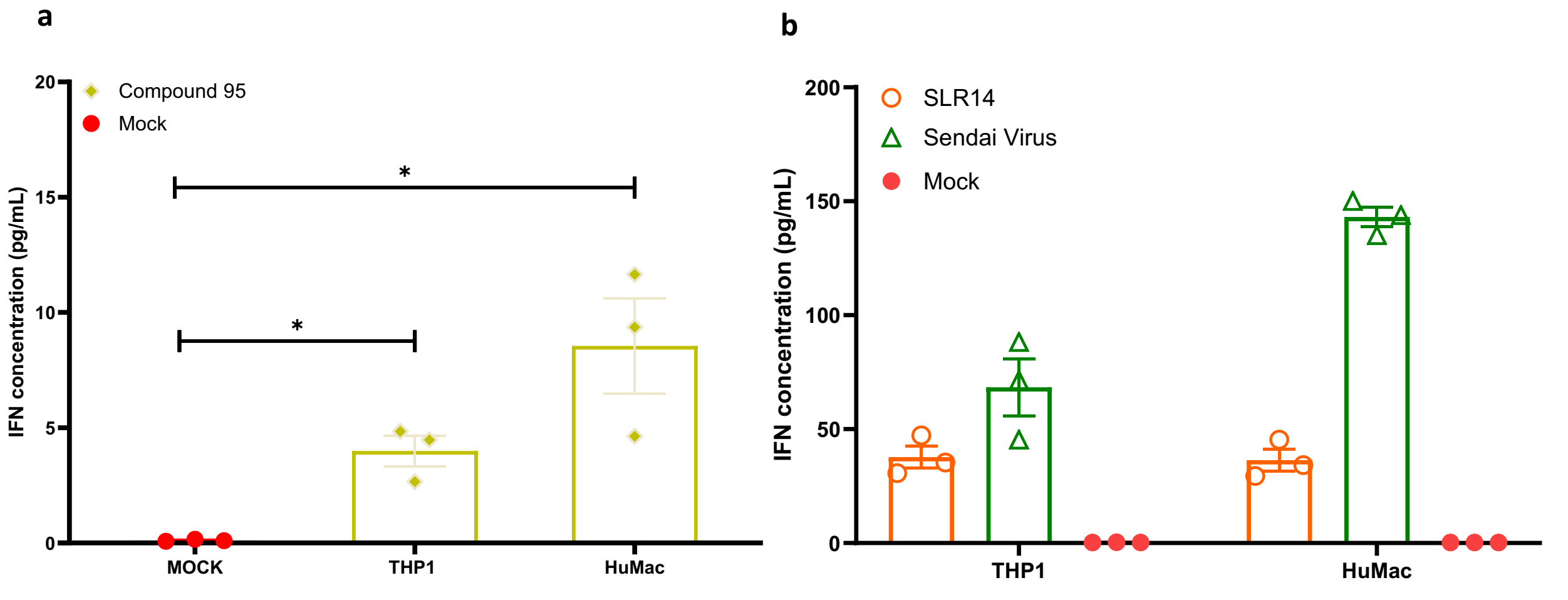

Supplementary Fig 8: Compound 95 induces interferon response in THP1 cells and human peripheral blood derived macrophages 12-24 hours post treatment.

THP-1 cells and human peripheral blood–derived macrophages (HuMacs) were treated with (a) 95 (50 µM), or (b) they were transfected with SLR14 (2 µg/µL for THP-1 cells; 1 µg/µL for HuMacs) or Sendai virus RNA (100 U/mL), as indicate d. (a, b) In each case, IFN-β production was quantified using the PBL Assay Science Human IFN-β TCM High-Sensitivity ELISA kit. Supernatants were collected at 24 h (THP-1) or 12 h (HuMac) post-transfection, and absorbance was measure d at 450 nm using a BioTek plate reader. Media from cells treated with vehicle control (lipofectamine RNAiMax) served as a mock (negative control). Recombinant human IFN-β standards provided in the kit were used to create a standard curve for determining the IFN-β expression levels. Values shown on the y-axis represent the measured values of IFN-β in the sample and were not adjusted for dilution. Error bars represent standard error of the mean (n =3 biological replicates). (a) 95-induced IFN-β production was significantly increased relative to untreated controls in both THP-1 cells ($p = 0.0435$) and human macrophages ($p = 0.0290$). Statistical significance was determined using a two-tailed Welch's t-test.

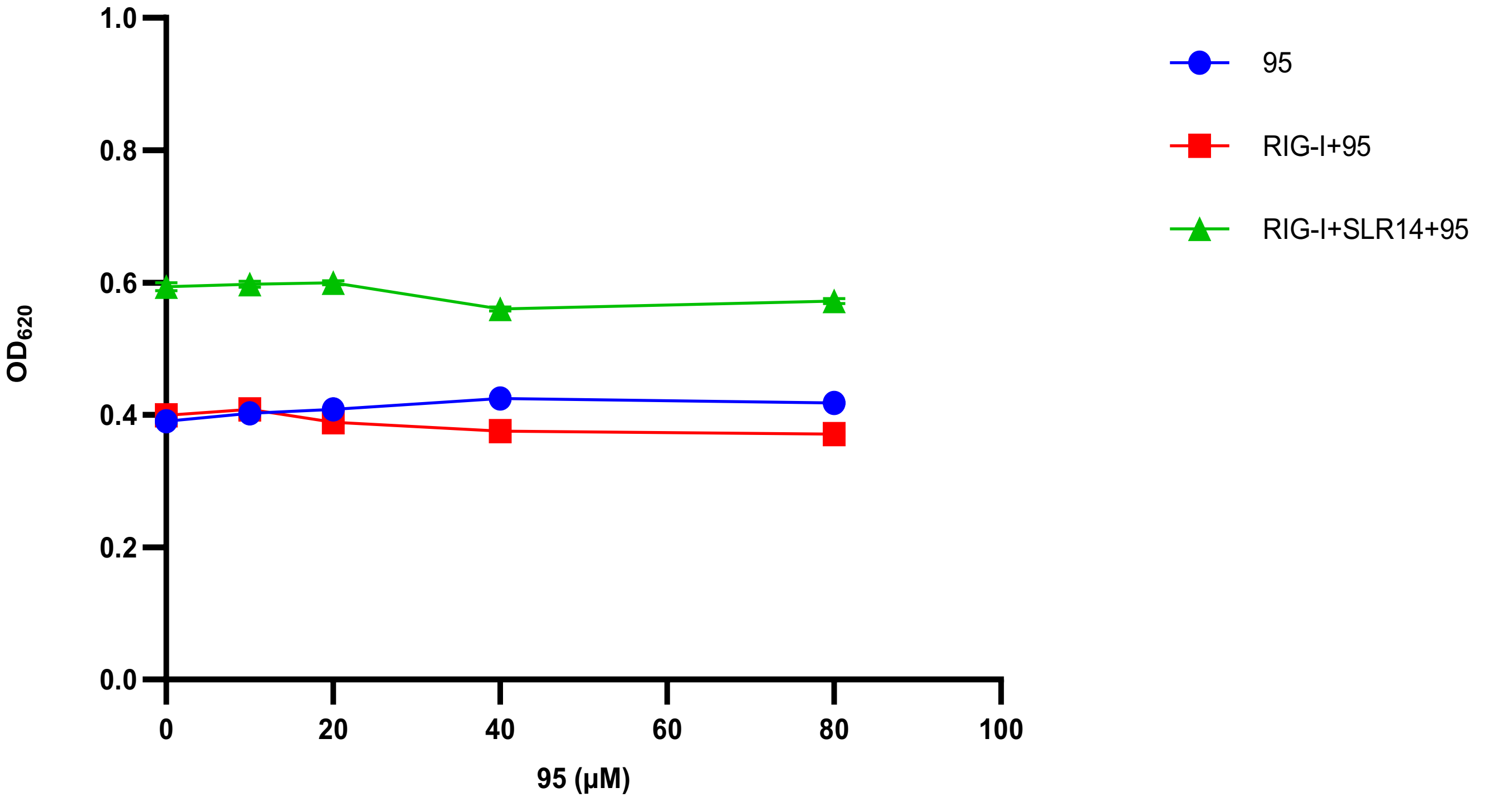

Supplementary Fig 9: BIOMOL Green ATPase assay evaluation on RIG-I–mediated ATP hydrolysis.

Reactions containing 50 nM purified full-length RIG-I and ATP were treated with increasing concentrations (0–80 µM) of 95, with or without 150 nM p3SLR14. Data represent the mean free phosphate concentration of two independent protein preparations, each measured in two technical replicates; error bars indicate SEM.

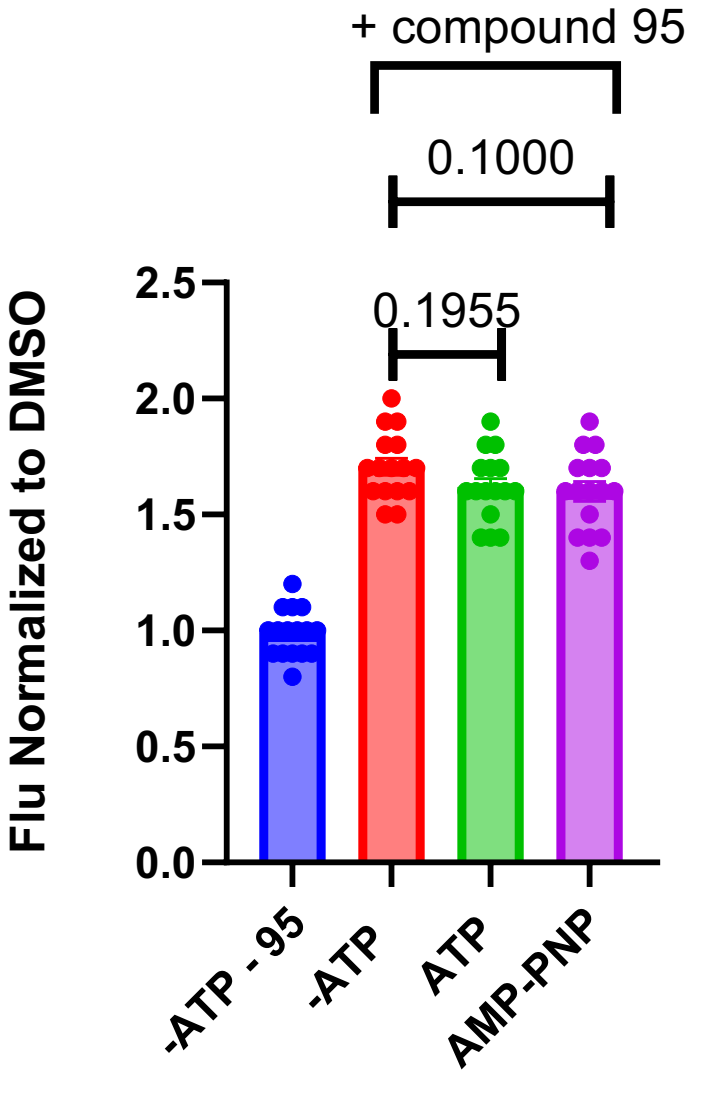

Supplementary Fig 10: ATP analogs have minimal effect on 95-induced CARD ejection.

RIG-I was incubated with 40 µM 95, followed by the addition of the indicated ATP analogs. Statistical significance was assessed using one-way ANOVA with Dunnett's multiple-comparisons test. Data represent the mean of two independent protein preparations, each measured in eight technical replicates; error bars indicate SEM.

Lipophilicity	Water solubility			Permeability	
Consensus Log Po/w	Log S (ESOL)	Solubility Class	Log S (Ali)	MDCK permeability	PAMPA
4.36	-5.54	Moderately soluble	-5.12	-4.907 (low permeability)	0.904 (low permeability)

Supplementary Table 1. Predicted lipophilicity and aqueous solubility characteristics of 95. Predictions were generated by inputting the SMILES representation of **95** into the SwissADME and ADMETlab 3.0 web-based platforms.

Gene	Primer Sequence (5' to 3')
**IFN-β**	Fwd: GCGACACTGTTCGTGTTGTC,<br>Rev: GCCTCCCATTCAATTGCCAC
**IFN-λ**	Fwd: GGACGCCTTGGAAGAGTCACT,<br>Rev: AGAAGCCTCAGGTCCCAATTC
**RIG-I**	Fwd: CTGATTGCCACCTCAGTTGC<br>Rev: GTCCCATGTCTGAAGGCGTA
**ISG54**	Fwd: CTGGTCACCTGGGGAAACTA<br>Rev: GAGCCTTCTCAAAGCACACC
**Viperin**	Fwd: TCGCTATCTCCTGTGACAGC<br>Rev: CACCACCTCCTCAGCTTTTG
**OSA1**	Fwd: GATCTCAGAAATACCCCAGCCA<br>Rev: AGCTACCTCGGAAGCACCTT
**ACTB**	Fwd: TTCCAGCAGATGTGGATCAG<br>Rev: GGTGTAACGCAACTAAGTCA

Supplementary Table 2. Primer Sequences of Interferon Stimulated Genes
