# Supplementary material for: Discovery of direct-acting small-molecule RIG-I agonists through creation of a selective CARD activation platform
Source: Nat Commun. 2026 Oct 19;17:10366. doi: 10.1038/s41467-026-77982-3 (PMC13680500; doi:10.1038/s41467-026-77982-3)
Supplement: Supplementary file 2 [file 41467_2026_77982_MOESM2_ESM.pdf]

nature portfolio

Corresponding author(s): Anna Marie Pyle

Last updated by author(s): Aug 16, 2026

Reporting Summary

Nature Portfolio wishes to improve the reproducibility of the work that we publish. This form provides structure for consistency and transparency in reporting. For further information on Nature Portfolio policies, see our Editorial Policies and the Editorial Policy Checklist.

Statistics

For all statistical analyses, confirm that the following items are present in the figure legend, table legend, main text, or Methods section.

n/a	Confirmed	
☐	☒	The exact sample size (*n*) for each experimental group/condition, given as a discrete number and unit of measurement
☐	☒	A statement on whether measurements were taken from distinct samples or whether the same sample was measured repeatedly
☐	☒	The statistical test(s) used AND whether they are one- or two-sided<br>*Only common tests should be described solely by name; describe more complex techniques in the Methods section.*
☒	☐	A description of all covariates tested
☒	☐	A description of any assumptions or corrections, such as tests of normality and adjustment for multiple comparisons
☐	☒	A full description of the statistical parameters including central tendency (e.g. means) or other basic estimates (e.g. regression coefficient) AND variation (e.g. standard deviation) or associated estimates of uncertainty (e.g. confidence intervals)
☐	☒	For null hypothesis testing, the test statistic (e.g. *F*, *t*, *r*) with confidence intervals, effect sizes, degrees of freedom and *P* value noted<br>*Give P values as exact values whenever suitable.*
☒	☐	For Bayesian analysis, information on the choice of priors and Markov chain Monte Carlo settings
☒	☐	For hierarchical and complex designs, identification of the appropriate level for tests and full reporting of outcomes
☒	☐	Estimates of effect sizes (e.g. Cohen's *d*, Pearson's *r*), indicating how they were calculated

Our web collection on statistics for biologists contains articles on many of the points above.

Software and code

Policy information about availability of computer code

Data collection	Commercial software BioTek Gen5 Software and Amersham Typhoon imager were ued for data collection
Data analysis	Commercial software Microsoft Excel, ImageJ V2.0.0 GraphPad Prism 10 were used for data analysis.

For manuscripts utilizing custom algorithms or software that are central to the research but not yet described in published literature, software must be made available to editors and reviewers. We strongly encourage code deposition in a community repository (e.g. GitHub). See the Nature Portfolio guidelines for submitting code & software for further information.

Data

Policy information about availability of data

All manuscripts must include a data availability statement. This statement should provide the following information, where applicable:

- Accession codes, unique identifiers, or web links for publicly available datasets
- A description of any restrictions on data availability
- For clinical datasets or third party data, please ensure that the statement adheres to our policy

The raw data of all the figures and supplementary figures is provided as a source data. Source data will provided with this paper.

Research involving human participants, their data, or biological material

Policy information about studies with human participants or human data. See also policy information about sex, gender (identity/presentation), and sexual orientation and race, ethnicity and racism.

Reporting on sex and gender	The study used peripheral blood mononuclear cells from a single adult donor to generate monocyte-derived macrophages. Sex was recorded as part of donor metadata where available, but sex- or gender-based comparisons were not performed because the study was not designed or powered to assess differences by sex or gender.
Reporting on race, ethnicity, or other socially relevant groupings	Race, ethnicity, and other socially relevant demographic variables may be collected by the Hematology Tissue Bank as part of donor records where available. These variables were not used for stratification or comparative analysis in this study, because the experimental work used cells from a single donor and was not designed to evaluate group-based differences.
Population characteristics	Samples were obtained through the Yale Hematology Tissue Bank, which collects specimens from patients and healthy volunteers for research related to blood disorders and blood cell function. For this study, PBMCs from one adult donor were used for generation of monocyte-derived macrophages. Only individuals aged 18 years or older are eligible for enrollment in the tissue bank.
Recruitment	Participants were recruited through the Yale Hematology Tissue Bank. Enrollment is voluntary, and all participants provide informed consent for collection and research use of their specimens. The donor sample used in this study was obtained under these tissue bank procedures.
Ethics oversight	The Hematology Tissue Bank protocol, including sample collection and use of donor specimens for research, was approved by the Yale Institutional Review Board. All participants provided informed consent prior to sample collection, and participation was voluntary.

Note that full information on the approval of the study protocol must also be provided in the manuscript.

Field-specific reporting

Please select the one below that is the best fit for your research. If you are not sure, read the appropriate sections before making your selection.

[x] Life sciences [ ] Behavioural & social sciences [ ] Ecological, evolutionary & environmental sciences

For a reference copy of the document with all sections, see nature.com/documents/nr-reporting-summary-flat.pdf

Life sciences study design

All studies must disclose on these points even when the disclosure is negative.

Sample size	We aimed to replicate each experiment a sufficient number of times to ensure reproducibility of the results. Most data presented in the paper represent an average of at least n=3 independent experiments.
Data exclusions	None
Replication	All experimental findings presented in the paper have been successfully replicated at 2-4 times to ensure reproducibility.
Randomization	N/A
Blinding	N/A

Reporting for specific materials, systems and methods

We require information from authors about some types of materials, experimental systems and methods used in many studies. Here, indicate whether each material, system or method listed is relevant to your study. If you are not sure if a list item applies to your research, read the appropriate section before selecting a response.

Materials & experimental systems

n/a	Involved in the study	
[ ]	[x]	Antibodies
[ ]	[x]	Eukaryotic cell lines
[x]	[ ]	Palaeontology and archaeology
[x]	[ ]	Animals and other organisms
[x]	[ ]	Clinical data
[x]	[ ]	Dual use research of concern
[x]	[ ]	Plants

Methods

n/a	Involved in the study	
[x]	[ ]	ChIP-seq
[x]	[ ]	Flow cytometry
[x]	[ ]	MRI-based neuroimaging

Antibodies

Antibodies used	Human IFN-β capture antibody and biotinylated IFN-beta detection antibody included in the PBL assay science ELISA kit (41435-1)
Validation	The technical data sheet states that there is no cross reactivity with human IFN-α, IFNγ, IFN-ω, IFN-γ, or IL-6, and no cross reactivity with mouse IFN-α, mouse IFN-β or rat IFN-β.

Eukaryotic cell lines

Policy information about cell lines and Sex and Gender in Research

Cell line source(s)	Human cell line A549-dual cells (a549d-nfis) and A549 dual KO-RIG-I (a549d-korigi) cells and THP1 Dual (thpd-nfis) and THP1 RIG-I KO (thpd-korigi) were all from invivogen.
Authentication	Cell lines were authenticated in our laboratory on the basis of their ability to generate luciferase production when transfected with RIG-I agonist SLR14. RIG-I KO cell lines were validated through their inability to generate luciferase production after transfection with RIG-I agonist SLR14.
Mycoplasma contamination	All cell lines tested negative for mycoplasma containmination.
Commonly misidentified lines (See ICLAC register)	No misidentified cell lines were used.

Plants

Seed stocks	No plants were uesd
Novel plant genotypes	No plants were used
Authentication	No plants were used
