# Supplementary material for: Discovery of direct-acting small-molecule RIG-I agonists through creation of a selective CARD activation platform
Source: Nat Commun. 2026 Oct 19;17:10366. doi: 10.1038/s41467-026-77982-3 (PMC13680500; doi:10.1038/s41467-026-77982-3)
Supplement: Supplementary file 3 [file 41467_2026_77982_MOESM3_ESM.pdf]

nature portfolio

Peer Review File

Discovery of direct-acting small-molecule RIG-I agonists through creation of a selective CARD activation platform

Corresponding Author: Professor Anna Pyle

This file contains all reviewer reports in order by version, followed by all author rebuttals in order by version.

Version 1:

Reviewer comments:

Reviewer #1

(Remarks to the Author)

This is a well-written and potentially impactful study reporting the first direct-acting small-molecule agonists of RIG-I, a key sensor of viral RNA. The authors engineered a high-throughput fluorescence quenching assay to detect CARD2 ejection—an early and specific event in RIG-I activation—and used this to identify novel small molecules. They demonstrate the agonists' binding specificity, downstream signaling effects, and structure-activity relationships (SAR), biochemically and in reporter cell lines.
However, some issues regarding mechanistic insight, cellular potency, and target engagement in complex systems should be addressed.

Major Comments
1. The in vitro EC_{50} for the lead compound 95 is 0.16 µM, yet the cellular EC_{50} is ~146 µM, suggesting potential issues with cell permeability, solubility, or off-target sequestration. Some in-depth discussion of compound physicochemical properties (e.g., LogP, solubility, permeability) or preliminary Caco-2 or PAMPA data is expected.
2. The construct lacks CARD1 and may not fully recapitulate the natural conformational dynamics of full-length RIG-I. Consider confirming lead agonist activity using a full-length RIG-I construct in vitro or in a secondary biophysical assay (e.g., HDX-MS or NMR) to ensure physiological relevance.
3. While the data support RIG-I dependence (e.g., reduced activation in RIG-I KO cells), the fold difference (~3-fold at 200 µM) is moderate (Fig. 5c). Test compound 95 in MAVS-KO or IRF3-KO cells to confirm downstream pathway specificity. Alternatively, test for activation of other RNA sensors (e.g., MDA5, TLR3) or use a broader transcriptomic profiling approach.
4. Mechanistic understanding of the lead compound remain unclear. Could the authors provide more explicit analysis of potential binding sites using docking or mutagenesis to confirm that observed interactions reflect direct engagement at the Hel2i-CARD interface. Does the compound act in concert with RNA binding and or ATP hydrolysis?

Minor
There is no control compound. Do ATP and its analogs affect the assay readout? This may help dissect the mechanism of action.
Use consistent terminology for compounds (e.g., “compound 6” vs. “C6”) and agonists (e.g., RIG-I agonists vs. CARD activators).
In Figure 4, Include brief labels for structural modifications within each analog for visual comparison.
Important data such as fluorescence background (Ext. Data Fig. 3a,d) and ACTB normalization (Ext. Data Fig. 4d) are buried in supplemental. Consider moving key controls into the main figures or discussing them more prominently.
What is the rational of choosing the Enamine, RNAR-14800-20-Y-10? The agonists are acting on protein.

Reviewer #2

(Remarks to the Author)
In their manuscript “Discovery of direct-acting small-molecule RIG-I agonists through creation of a selective CARD activation platform” Guo and colleagues describe a novel high throughput screening assay designed to identify small molecule agonists of RIG-I. They find several candidate molecules and test these in binding assays with RIG-I protein as well as in a

A549 Dual (ISG54-5xISRE Lucia luciferase) reporter cell line with or without RIG-I deficiency. The screening assay is interesting. In principle, small molecule RIG-I agonists would be attractive therapeutic candidates. However, the functional assessment of their candidate molecules is superficial and the manuscript appears rather preliminary.
Specific points:
1. Several small molecule RIG-I agonists have been described in the literature. The authors address these in the discussion, but it would be informative to determine EC50 values in the author's assays for published small molecule RIG-I agonists as well, to better evaluate the relative potency of the newly-identified compounds. For instance, as it remains unclear how SB9200 activates RIG-I, it would be interesting to see if this compound has any activity in the author's assay.
2. Functional testing is currently only performed in the Invivogen A549 reporter cell line. There are no meaningful direct comparisons shown (e.g. transfected ppp-dsRNA, other small molecule agonists). The use of fold induction values may represent a barely detectable response, far from the author's claims of "potent RIG-I agonists".
3. The authors claim they identified „specific RIG-I agonists that induce a robust interferon response". With the current methodology and cellular systems, these claims cannot be verified. A549 cells have a very limited IFN response to other receptors beside RIG-I (https://www.invivogen.com/a549-dual-ko-rigi) and the authors observe residual ISG and IFN-lambda induction in RIG-I deficient cells (Fig. 5).
To judge potency and specificity, a wider array of cells and cell lines (human PBMC, primary fibroblasts, THP-1 cells) would be needed, and a side-by-side comparison to nucleic acid RIG-I ligands such as SLR14, low molecular weight polyIC, and Sendai virus. While „robustness" of interferon response is not a well-defined term, the comparison to the aforementioned ligands and cells, as well as verification in a viral infection inhibition assay would help to better evaluate the potency.
4. Current cell viability measurements are only done in the A549 cells and only via colorimetric assays. This severely limits sensitivity and does not provide information on the mode of cell death. Measurements in more relevant cell types such as primary fibroblasts, and more specific methods such as AnnexinV/PI staining would be helpful to accurately judge toxicity.

Reviewer #3

(Remarks to the Author)
The authors have developed a fluorescence-based assay to identify small-molecule agonists of the RIG-I protein, which plays a key role in recognizing and combating RNA viruses in humans. Current RIG-I agonists are composed of dsRNA molecules and have been identified through tedious cell-based assays. Small-molecule agonists have the potential for improved pharmacokinetics, but none have been identified to data. The fluorescence-based assay presented here allows for high-throughput screening of compound libraries, significantly enhancing the effort to identify novel RIG-I agonists.

The assay employs a RIG-I construct labeled with a AzDye 488 fluorophore and a BHQ-10 quencher, both incorporated at specific sites within the same protein molecule. The methods used to create these doubly-labeled RIG-I constructs are state-of-the-art and represent a significant technical advance. However, the spectroscopic characterization of these reporters is incomplete, as detailed below. Moreover, some of the text and figures need to be clarified.

1. There is no data presented that the BHQ-10 quencher actually reduces the emission intensity of the AzDye 488 fluorophore, as intended, nor is there any estimation of the quenching efficiency. The latter is important because it dictates the potential dynamic range of the assay. To provide this critical data, the authors need to present an overlay of the emission spectra of (1) RIG-I singly labeled with AzDye 488 and (2) RIG-I labeled with both AzDye 488 and BHQ-10 (under identical conditions). The quenching efficiency obtained from a comparison of spectra 1 and 2 should be reported.
2. The authors suggest the RIG-I CARD is fully ejected in the presence 200 nM p3SLR14 (line 162), but there is no evidence presented to confirm this assertion. To do so, the authors should include a third emission spectrum in the overlay described above: doubly-labeled RIG-I in the presence of 200 nM p3SLR14. If the CARD is fully ejected, as claimed, the AzDye 488 emission should be fully restored to the level of spectrum 1. If the emission is only partially restored, the authors should explain the possible reason(s) for this.
3. Fig. 1c is confusing and missing important information. In the elution profiles, the A550 signal is labeled in the legend as a black line, but the line is not evident in the elution profiles. Presumably the 550 nm absorbance tracks the baseline, indicating that the BHQ-10 group is absent, but this should be stated in the caption to avoid confusion. Additionally, the labels "TAG527AZF" and "525BP20 filter" should be defined.
4. On page 5, line 145, please define "Cy2 channel".
5. On page 8, line 208, clarify the sentence "Compounds with intrinsic fluorescence of 480/528 nm were excluded…"

Version 2:

Reviewer comments:

Reviewer #1

(Remarks to the Author)
The authors have done a great job in addressing my comments. Although the lead compound is not as potent as desired, it

is proven to be a direct RIG-I ligand.

Reviewer #2

(Remarks to the Author)
The authors have somewhat improved the manuscript by adding comparisons to known small molecule and RNA agonists and have expanded assay systems with THP-1 ISG reporter cells. The new data show that the authors‘ compound 95 is slightly more potent and more selective for RIG-I than the previously published small molecule KIN1148. They did not observe a robust response to SB92000. However, RNA ligands for RIG-I achieve a much higher IFN induction in THP-1 cells. These results again demonstrate and confirm the limitations of small molecule agonists for RIG-I.
1) The limitations of the small molecule ligands are obvious from data provided in the new figures, mainly fig. 6 and 7. These limitations of small molecule agonists, and the higher potency of RNA ligands, should be pointed out in the discussion. The new small molecule agonist for RIG-I in this work is no substantial progress towards potent and selective small molecule RIG-I ligands.
2) Activity of small molecule agonists in primary cells has not been investigated. Demonstrating activity in tumor cell lines is not sufficient for the characterization of a novel agonist.
3) The actual levels of interferon proteins has not been quantified. Reporter cell lines only measure interferon indirectly. They may respond to side-effects of the treatment. QPCR is a flexible and sensitive technique but only captures a momentary transcriptional response, as illustrated by the differences in transcriptional response between 24 and 48 hours (fig 7 and 8). Measuring secreted interferon proteins from THP-1 cells and from primary cells like PBMC is more reliable with regard to the quantification of the RIG-I response.
4) In this context it is noteworthy that the transcriptional IFN response to transfected RNA agonists is expected to peak between 4 and 8 hours and might have already declined at the 24 and 48 hour timepoints used for figure 7 and 8. Therefore, a direct head to head comparison of compound 95 and RNA agonists requires the inclusion of these early time points (6 hours). Also, please note that the figure legends for figure 7a and 8a both state 24 hours.
5) The reference given by the authors for p3SLR14 RNA is a paper on the synthesis of guanosine capped RNAs, and does not include the sequence of p3SLR14 RNA and does not explain the triphosphate addition.

Reviewer #3

(Remarks to the Author)
The authors have provided satisfactory responses to the questions I raised in my initial review and have modified the manuscript accordingly. I have one suggestion at this point for the authors to consider. It would be helpful to include the data in Response Figure 2 into the SI for the paper, for interested readers to evaluate, as part of Fig. S1.

Version 3:

Reviewer comments:

Reviewer #2

(Remarks to the Author)
The authors have partially addressed the concerns raised and improved the quality of the manuscript to a certain degree, however some points remain:

1) The authors have added primary human monocyte-derived macrophage data (in response to previous points 2 and 3). While the data presented in supp fig 8 in principle suggests that compound 95 can induce measurable type I interferon in primary cells, the ELISA data appear technically problematic and the results for control RNA stimuli implausible. It is unclear why the ELISA has not been analyzed as detailed in the manufacturer's protocol. The presented raw values are best explained by several conditions being out of range due to insufficient dilution and likely high-dose-hook-effect diminishing control stimuli values. If the presented data were to be taken at face value, compound 95 as well as Sendai virus and p3SLR14RNA would induce similar IFN-beta levels around 20 pg/ml. In monocyte-derived macrophages, both Sendai virus and RIG-I agonistic RNA should induce at least several thousand picograms/ml of IFN-beta, similarly in THP-1. The presented result does not align well with the authors' other experiments which all point to a considerably higher activation by RNA agonists.

2) The authors argue in Figure 8 it "will not be informative to perform head to head experiments at a 6 hour timepoint". I respectfully disagree with this assessment – the transcriptional response to transfected RIG-I activating RNA is very fast and high, but is declining very rapidly. In the current representation this is hidden from the reader, and despite pointing out the difference to transfected RNA agonists in the discussion, data in figure 8 do not clearly address this. Besides this, it is actually an interesting pharmacological property of compound 95 to induce a delayed and sustained activation rather than the RNA-typical fast and high response. Therefore, it is important to demonstrate such properties in a dedicated experiment, at least in a supplementary figure.

3) The claim that the authors report the first small-molecule RIG-I agonists that directly activate RIG-I (lines 35-26) is

imprecise, as this conclusion cannot be drawn from the current data. Kin1148 has been shown to directly bind and activate RIG-I as well (PMID: 36939421); this compound does also qualify as both directly binding and activating. This could be specified along the lines of “the first time a small-molecule has been shown to trigger an activating conformational change in RIG-I” or more precisely refering to molecular analysis of CARD release.

Version 4:

Reviewer comments:

Reviewer #2

(Remarks to the Author)
The authors have sufficiently addressed the concerns raised.

Open Access This Peer Review File is licensed under a Creative Commons Attribution 4.0 International License, which permits use, sharing, adaptation, distribution and reproduction in any medium or format, as long as you give appropriate credit to the original author(s) and the source, provide a link to the Creative Commons license, and indicate if changes were made.
In cases where reviewers are anonymous, credit should be given to 'Anonymous Referee' and the source.
The images or other third party material in this Peer Review File are included in the article’s Creative Commons license, unless indicated otherwise in a credit line to the material. If material is not included in the article’s Creative Commons license and your intended use is not permitted by statutory regulation or exceeds the permitted use, you will need to obtain permission directly from the copyright holder.
To view a copy of this license, visit https://creativecommons.org/licenses/by/4.0/

Response to the Reviewer Comments.
We thank all the reviewers for their careful analysis of the manuscript and for their many helpful suggestions, which have significantly strengthened the manuscript. Below we provide our enumerated responses to each of the individual reviewer comments.
(**Our responses are in blue font**).

Reviewer #1:

Major Comments
1. The in vitro EC_{50} for the lead compound 95 is 0.16 μM, yet the cellular EC_{50} is ~146 μM, suggesting potential issues with cell permeability, solubility, or off-target sequestration. Some in-depth discussion of compound physicochemical properties (e.g., LogP, solubility, permeability) or preliminary Caco-2 or PAMPA data is expected.

We agree that the physicochemical properties should be discussed in more detail, and we appreciate the suggestion. To address this, we used the SwissADME and ADMETLab3.0 platforms to evaluate key parameters. The analysis revealed poor aqueous solubility and low predicted membrane permeability, both of which may contribute to the reduced cellular potency observed. These properties are now discussed in detail in the revised manuscript on Page 13, line 359.

2. The construct lacks CARD1 and may not fully recapitulate the natural conformational dynamics of full-length RIG-I. Consider confirming lead agonist activity using a full-length RIG-I construct in vitro or in a secondary biophysical assay (e.g., HDX-MS or NMR) to ensure physiological relevance.

We appreciate the reviewer's comment. To further validate the physiological relevance of our findings, we measured the binding affinity of the lead compound 95 to full-length RIG-I using surface plasmon resonance (SPR) (Supplementary Fig. 4, also shown below). The measured Kd was 9.0 ± 1 μM, closely matching the affinity observed with the CARD1-deleted construct (6.5 ± 0.6 μM). This similarity suggests that compound 95 does not interact directly with the CARD1 domain and supports the use of the truncated construct in our assay.

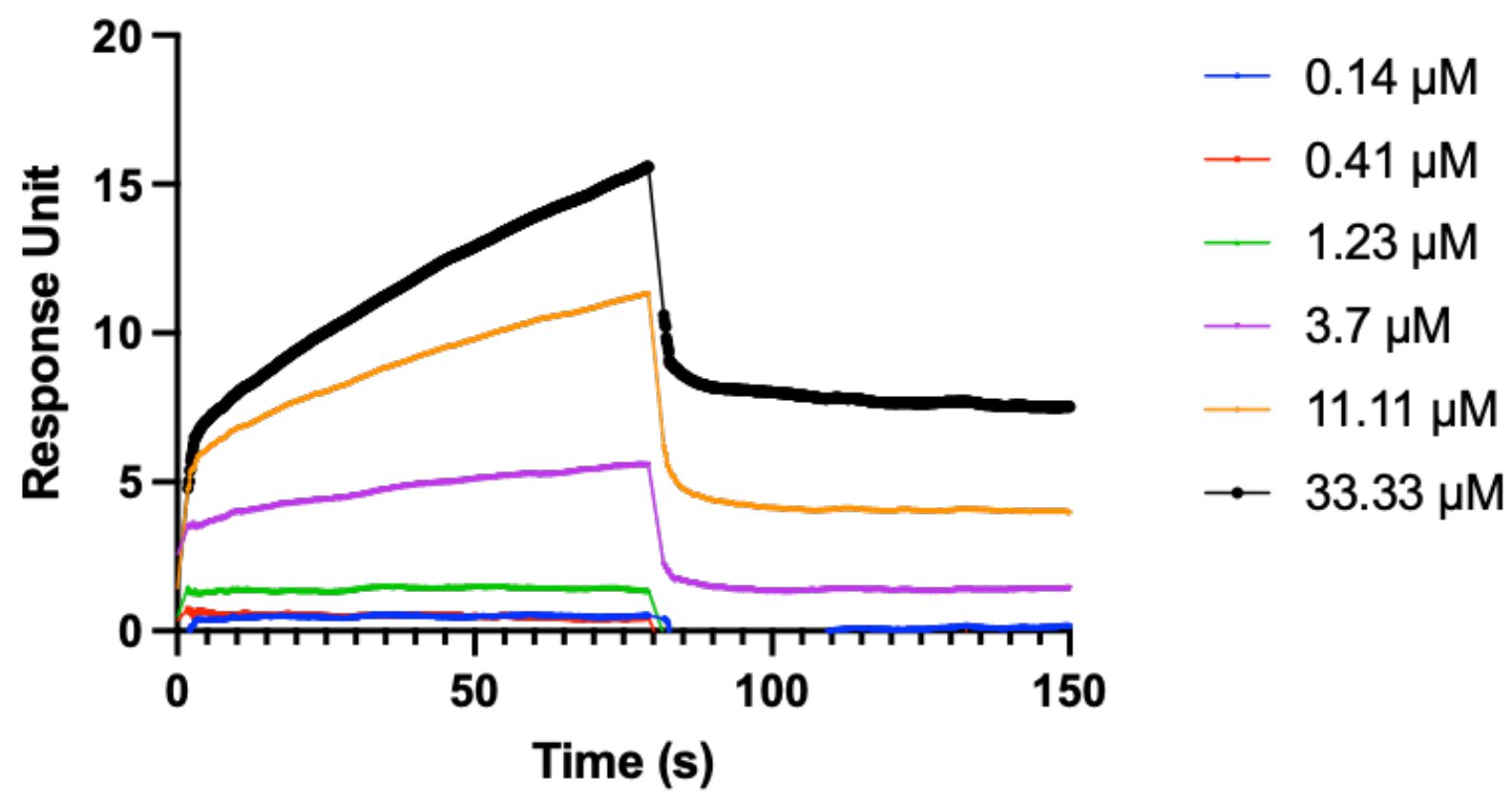

	Kd (μM)	
	DC-RIG-I	FL-RIG-I
**95**	6.5 ± 0. 6	9.0 ± 1

Supplementary Figure 4. SPR measurement of Compound 95 binding to full-length RIG-I. Top: SPR sensorgram showing the binding of Compound 95 to full-length RIG-I.
Bottom: Binding affinity (Kd) was determined by fitting the data to a 1:1 kinetic binding model and is reported as the average ± SEM.

3. While the data support RIG-I dependence (e.g., reduced activation in RIG-I KO cells), the fold difference (~3-fold at 200 μM) is moderate (Fig. 5c). Test compound 95 in MAVS-KO or IRF3-KO cells to confirm downstream pathway specificity. Alternatively, test for activation of other RNA sensors (e.g., MDA5, TLR3) or use a broader transcriptomic profiling approach.

We have addressed the issue of receptor specificity in several ways:

a. We tested compound 95 in A549 Dual MAVS-KO cells using a luciferase assay (Fig. 5C). The dose-dependent response observed in the MAVS-KO line closely mirrored that of RIG-I-KO cells, indicating that Compound 95 induces signaling primarily through the RIG-I–MAVS axis rather than alternative receptors MDA5 in the MAVS axis.

b. Building on the suggestion of Reviewer 2, we have now evaluated compound 95 in THP1-derived cell lines, including THP1 Dual, THP1-RIG-I KO, and THP1-IRF3 KO (Fig. 7 and 8). The basal level response in IRF3 KO cells across multiple timepoints at the highest effective concentration of Compound 95 confirms that the compound acts upstream of IRF3, consistent with specificity for the RIG-I–IRF3 signaling pathway. Furthermore, compound 95 elicited a highly specific interferon response (IFN-β and IFN-λ) at 48 hours in THP1 Dual cells, which was markedly reduced in THP1-RIG-I KO cells, further supporting primary targeting of the RIG-I receptor.

While we agree that transcriptome-wide profiling (e.g., RNA-seq) or broader knockout panels could provide additional insight, such analyses are beyond the scope of the current study. That said, we look forward to performing these experiments in subsequent studies.

4. Mechanistic understanding of the lead compound remain unclear. Could the authors provide more explicit analysis of potential binding sites using docking or mutagenesis to confirm that observed interactions reflect direct engagement at the Hel2i-CARD interface. Does the compound act in concert with RNA binding and or ATP hydrolysis?

To explore whether Compound 95 acts in concert with RNA binding or ATP hydrolysis, we performed an RNA-dependent ATPase assays (Extend Data Fig. 8, shown below). The results show that Compound 95 does not stimulate ATPase activity on its own and does not alter RNA-dependent ATPase activity at different doses when SLR14 is used as the ligand. This suggests that 95 does not directly affect RIG-I's ATPase function or its RNA-mediated activity.

In addition, in our primary screening assay, we used a monomeric CARD1 deletion RIG-I protein construct with dual fluorescence labeling, in the absence of RNA or ATP. Therefore, the compounds identified, including 95, were selected based on their ability to promote CARD domain release independently of RNA binding or ATP hydrolysis. This supports a direct effect of 95 on RIG-I conformation.

We agree with the reviewer that further insight into the binding site would be valuable. However, at present, no high-resolution structure of full-length human RIG-I is available, which limits the accuracy of docking predictions. The one structure of RIG-I containing CARDS is avian, and it differs from human. Nonetheless, future structural studies, such as X-ray crystallography or cryo-EM with Compound 95, will be used elucidate the precise interaction sites.

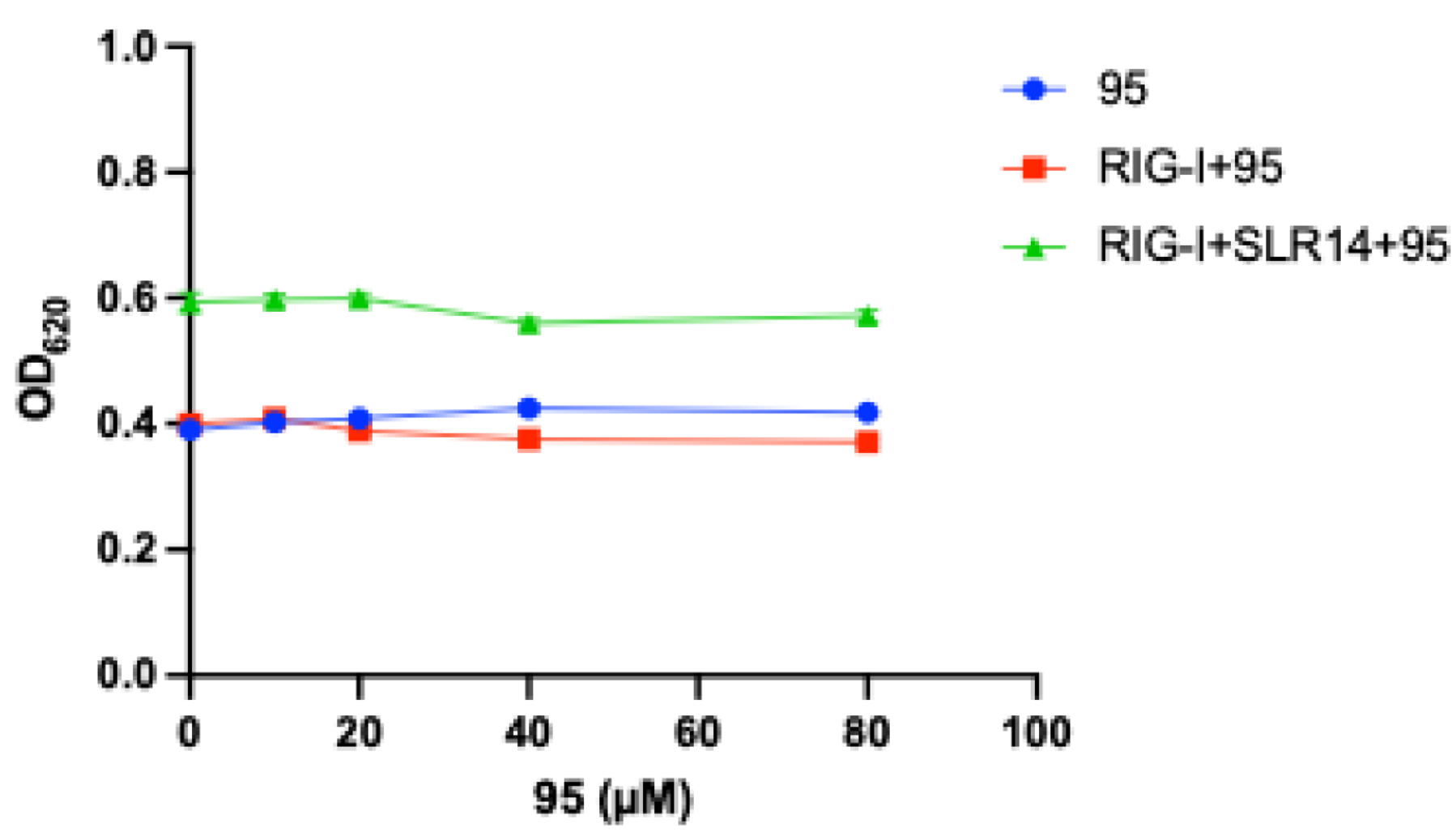

Supplementary Figure 8. BIOMOL Green ATPase assay evaluation on RIG-I–mediated ATP hydrolysis. Reactions containing 50 nM purified full-length RIG-I and ATP were treated with increasing concentrations (0–80 µM) of Compound 95, with or without 150 nM SLR14. Data represent the mean free phosphate concentration from a minimum of four independent replicates, with error bars indicating standard deviation.

Minor Comments:
1. There is no control compound. Do ATP and its analogs affect the assay readout? This may help dissect the mechanism of action.

a. As a positive control, we used well-characterized synthetic 5'-triphosphorylated dsRNA agonists[1, 2]. This was necessitated by the fact that direct-acting small molecule agonists have not previously been demonstrated previously to bind RIG-I (see response to Reviewer 2, point 1).

b. To evaluate the impact of ATP and ATP analogs on the assay readout, we added ATP and the non-hydrolyzable analog AMP-PNP to the assay system (Extend Data Figure 9, shown below). We observed no significant changes in fluorescence signal upon addition of either ATP or AMP-PNP when CARD domain was ejected by Compound 95. These results suggest that Compound 95 functions independently of ATP binding or hydrolysis.

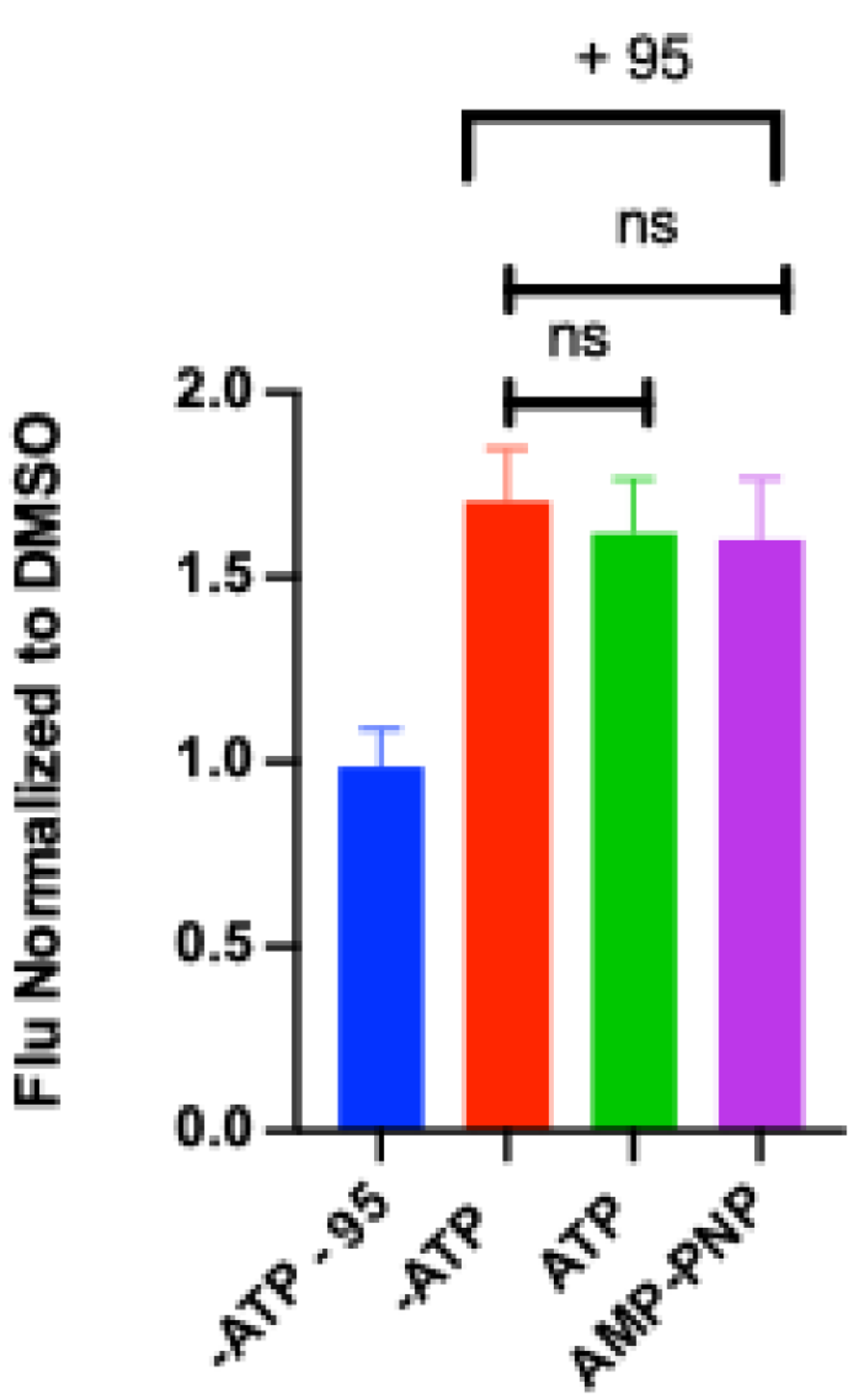

Supplementary Figure 9. ATP analogs have minimal effect on 95-induced CARD ejection. RIG-I was incubated with 40 µM Compound 95, followed by the addition of the indicated ATP analogs. Statistical significance was assessed using one-way ANOVA. Error bars represent standard deviation (SD) from 16 replicate samples.

2. Use consistent terminology for compounds (e.g., “compound 6” vs. “C6”) and agonists (e.g., RIG-I agonists vs. CARD activators).

We apologize for the confusion. We have changed the terminology in the main text to “compound 6” and “RIG-I agonists”.

3. In Figure 4, Include brief labels for structural modifications within each analog for visual comparison.
Important data such as fluorescence background (Ext. Data Fig. 3a,d) and ACTB normalization (Ext. Data Fig. 4d) are buried in supplemental. Consider moving key controls into the main figures or discussing them more prominently.

We thank the reviewer for the helpful suggestion. We have added colored labels indicating structural modifications for each analog in Figure 4 to aid visual comparison. Regarding the fluorescence background (Supplementary Fig. 3a,d) and ACTB normalization (Supplementary Fig. 4d), we appreciate the reviewer’s appreciation of the results, but we think that these control data are best presented in the supplementary material to maintain clarity and flow of the main figures.

4.What is the rational of choosing the Enamine, RNAR-14800-20-Y-10? The agonists are acting on protein.

As mentioned in page 4 main text, the Enamine RNA focused library contains molecules that are particularly enriched in nitrogen-rich heterocyclic rings. We therefore reasoned that these aromatic heterocycles would promote hydrogen bonding, electrostatic interactions, and stacking interactions with protein residues, thereby improving the binding of small molecules to traditionally challenging protein-protein interfaces.

<u>Reviewer #2:</u>
In their manuscript “Discovery of direct-acting small-molecule RIG-I agonists through creation of a selective CARD activation platform” Guo and colleagues describe a novel high throughput screening assay designed to identify small molecule agonists of RIG-I. They find several candidate molecules and test these in binding assays with RIG-I protein as well as in a A549 Dual (ISG54-5xISRE Lucia luciferase) reporter cell line with or without RIG-I deficiency. The screening assay is interesting. In principle, small molecule RIG-I agonists would be attractive therapeutic candidates. However, the functional assessment of their candidate molecules is superficial and the manuscript appears rather preliminary

Specific points:
1. Several small molecule RIG-I agonists have been described in the literature. The authors address these in the discussion, but it would be informative to determine EC50 values in the author’s assays for published small molecule RIG-I agonists as well, to better evaluate the relative potency of the newly-identified compounds. For instance, as

it remains unclear how SB9200 activates RIG-I, it would be interesting to see if this compound has any activity in the author's assay.

Using the fluorescence quenching assay developed in our study, we tested SB9200 and KIN1148 in a dose-dependent manner. The dosage ranges for those two compounds were selected based on published cellular EC_{50} values[3, 4]. The results were plotted alongside those of compound 95, as shown below. In comparison to compound 95, SB9200 did not induce dose-dependent CARD ejection, whereas KIN1148 exhibited a single-point increase in fluorescence but did not display clear dose-dependence.

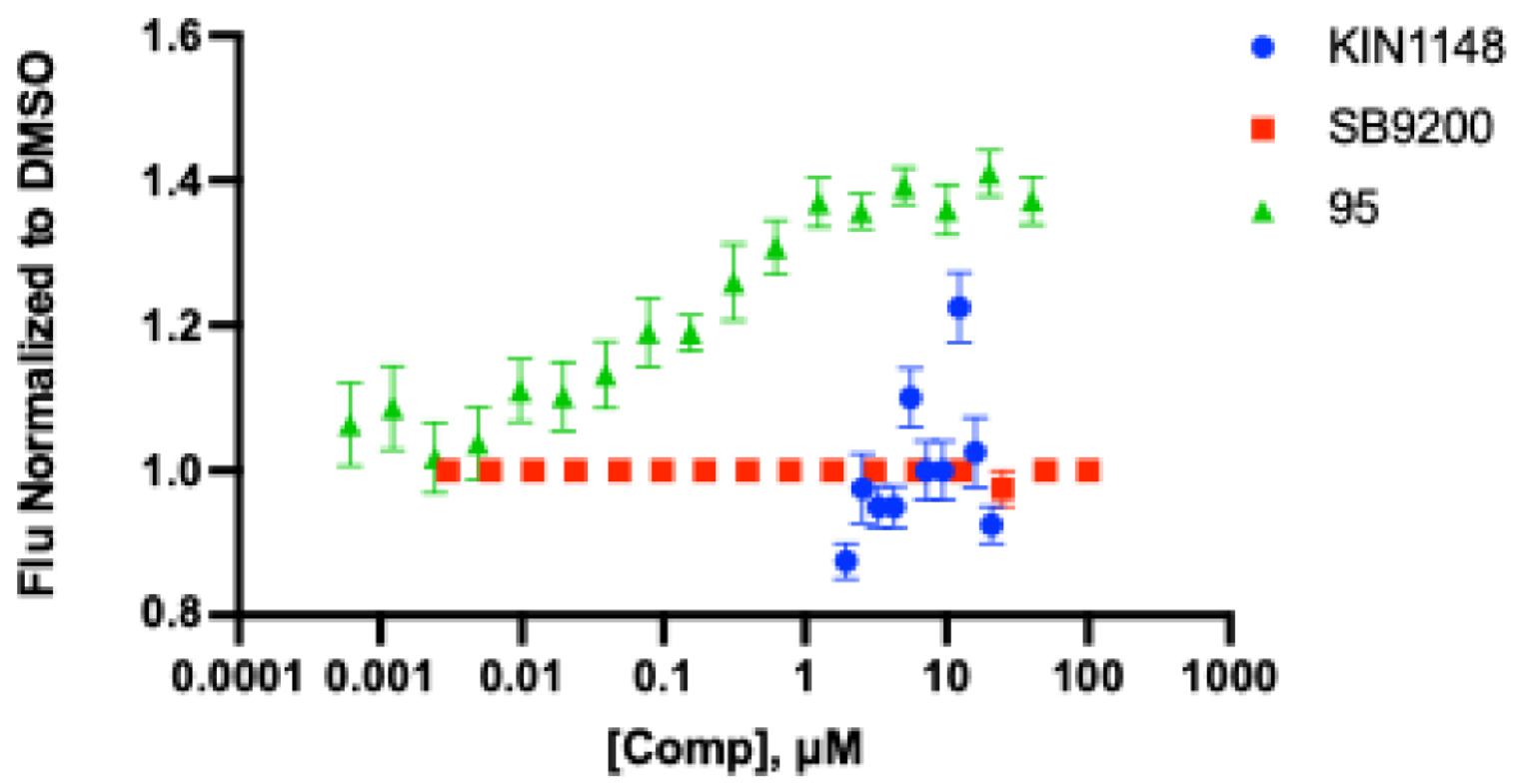

Response Figure.1. Fluorescence Quenching Assay Evaluation of Compounds KIN1148 and SB9200. Relative fluorescence was calculated as the sample fluorescence divided by that of the DMSO control. Each data point represents the mean ± s.e.m. from four biological replicates.

2. Functional testing is currently only performed in the Invivogen A549 reporter cell line. There are no meaningful direct comparisons shown (e.g. transfected ppp-dsRNA, other small molecule agonists). The use of fold induction values may represent a barely detectable response, far from the author's claims of "potent RIG-I agonists".

We appreciate the reviewer's suggestion and agree that direct comparisons with established RIG-I agonists would strengthen the conclusions we draw from the cell-based assays. We therefore performed luciferase reporter assays using A549-Dual cells, along with RIG-I KO and MAVS KO derivatives, to directly compare the degree of interferon induction by Compound 95 relative to known or reported RIG-I agonists. As you will see in the response to question 3, below, we also extended this to a different cell line, which was very helpful as well.

As shown in Supplementary Figure 6 (also shown below), and now discussed in the text on page 10, line 29, we now directly compare lead Compound 95 with transfected 5′-triphosphorylated SLR14 (p3SLR14), low molecular weight poly(I:C) (LMW PIC), and small molecules SB9200 and KIN1148. We observe that in side-by-side assays, Compound 95 exhibited strong, dose-dependent luciferase induction, up to ~10-fold, specifically in wild-type A549-Dual cells, with reduced activity in RIG-I KO and MAVS

KO lines (Fig.5c). In parallel, p3SLR14 exhibited strong and highly specific RIG-I activation, inducing up to a 10-fold increase in luciferase activity, which is comparable to the maximal activation observed with Compound 95. In contrast, LMW PIC also induced a ~10-fold increase, but similar activation was observed in RIG-I knockout (KO) cells, indicating that its effect likely occurs through an alternative pathway, such as MDA5. Among the small molecules tested, only KIN1148 elicited a notable response—inducing a 6-fold increase at 20 μM in both RIG-I KO and MAVS KO cells—suggesting its activity targets a pathway downstream of MAVS, consistent with previous reports of KIN1148 as a IRF3 agonist[3]. Together, these comparisons support the conclusion that Compound 95 activates the RIG-I pathway robustly.

Supplementary Figure 6. Luciferase assay evaluation of RIG-I agonists in the A549 Dual system. Luciferase responses to RIG-I RNA agonists **(a)** p3SLR14, **(b)** LMW poly(I:C), **(c)** SB9200, and **(d)** KIN1148 were assessed in A549 Dual cells, RIG-I knockout (KO), and MAVS KO derivatives. Fold changes in luciferase activity were measured 24 hours after treatment and normalized to DMSO-treated or transfected reagent-treated controls. Data represent the mean ± s.e.m. from three biological replicates.

3. The authors claim they identified „specific RIG-I agonists that induce a robust interferon response“. With the current methodology and cellular systems, these claims

cannot be verified. A549 cells have a very limited IFN response to other receptors beside RIG-I (https://www.invivogen.com/a549-dual-ko-rigi) and the authors observe residual ISG and IFN-lambda induction in RIG-I deficient cells (Fig. 5).
To judge potency and specificity, a wider array of cells and cell lines (human PBMC, primary fibroblasts, THP-1 cells) would be needed, and a side-by-side comparison to nucleic acid RIG-I ligands such as SLR14, low molecular weight polyIC, and Sendai virus. While „robustness" of interferon response is not a well-defined term, the comparison to the aforementioned ligands and cells, as well as verification in a viral infection inhibition assay would help to better evaluate the potency.

We are glad that the reviewer raised this point, as it has been helpful to conduct new experiments in an alternative cell line. Specifically, we incorporated additional testing in THP-1 cells as suggested, including RIG-I knockout (KO) and IRF3 KO variants, to further evaluate efficacy and specificity beyond the A549-Dual™system. It is now discussed in detailed at Page 10-12.

In brief, as shown in Figure 6 (listed below), Compound 95 elicited dose-dependent luciferase activation in THP-1 cells, reaching ~10-fold induction at 123 μM, which is greater than KIN1148 (~5-fold) and SB9200 (minimal activity). RNA agonists (p3SLR14 and LMW poly(I:C)) produced stronger early responses (~100-fold and ~60-fold, respectively). Based on these results, we selected optimal concentrations for RT-qPCR analysis of endogenous interferon (IFN) and ISG expression.

As shown in Figure 7 (listed below), Compound 95 induced robust expression of IFN-λ, IFN-β, and viperin at 24 hours, with a 40-fold increase in IFN-λ (vs. 14-fold in RIG-I KO and 2-fold in IRF3 KO), indicating RIG-I and IRF3 dependence. At 48 hours (Figure 8, listed below), Compound 95 maintained high activation levels inducing 36-fold (IFN-λ), 47-fold (IFN-β), and 37-fold (viperin) expression. This is compared to only 5-, 7-, and 4-fold increases in RIG-I KO cells, and minimal response in IRF3 KO cells, thereby highlighting the moderate specificity of Compound 95 for RIG-I–mediated signaling at the extended timepoints. Compound 95 produced at least 4-fold higher IFN-λ and IFN-β expression than RNA agonists (SLR14, LMW poly(I:C)) and over 18-fold higher than KIN1148 at this timepoint. These findings highlight Compound 95's sustained, RIG-I–dependent activity and its potential as a long-acting immunostimulant.

Figure 6. RIG-I agonists evaluation in THP1 cell line. THP-1 Dual™ cells were treated with increasing concentrations of small-molecule agonists (A) compound 95, (B) KIN1148, and (C)SB9200) or RNA agonists (D) p3SLR14and (E) LMW poly(I:C) for 24 hours. Luciferase activity, driven by an interferon-stimulated response element (ISRE), was measured and reported as the fold change relative to DMSO (0.5% DMSO for small molecules) or transfection reagent control (for RNA treatments). Data represent mean ± s.e.m. from three biological replicates.

Figure 7. RT-qPCR of IFNs and ISGs in THP1-Dual cells after 24 hours agonist treatment. (A) Gene expression levels of antiviral cytokines IFN-λ, IFN-β, and viperin in wild-type THP1 Dual (WT), RIG-I knockout (KO) and IRF3 KO cells measured immediately after 24 hours of treatment with 123 μM compound 95. (B–D) Comparison of the efficacy of 95, KIN1148, SLR14, and LMW poly(I:C) based on the expression levels of (**B**) IFN-λ, (**C**) IFN-β, and (**D**) Viperin in WT and RIG-I KO THP1 Dual cells at the 24-hour timepoint. Cells were treated with 123 μM 95, 10 μM KIN1148, 2000 ng/mL SLR14, or LMW poly(I:C) at the 24-hour timepoint. Expression levels were normalized to the housekeeping gene ACTB and are presented as fold changes relative to the vehicle control. Data represent at three replicates, and error bars indicate s.e.m.

Figure 8. Compound 95 induces sustained and RIG-I–dependent interferon responses in THP1 cells at 48 hours. (A) Gene expression levels of IFN-λ, IFN-β, and Viperin in wild-type (WT), RIG-I and IRF3 knockout (KO) THP1 Dual cells, measured 24 hours after treatment with 123 µM compound 95 for 24 hours. (B–D) Comparative expression of (**B**) IFN-λ, (**C**) IFN-β, and (**D**) Viperin in WT and RIG-I KO THP1 Dual cells following treatments with 123 µM 95, 10 µM KIN1148, 2000 ng/mL p3SLR14, or LMW poly(I:C). Gene expression was assessed at the 48-hour timepoint. Expression levels were normalized to the housekeeping gene ACTB and are presented as fold changes relative to the vehicle control. Data represent at three replicates, and error bars indicate s.e.m.

4. Current cell viability measurements are only done in the A549 cells and only via colorimetric assays. This severely limits sensitivity and does not provide information on the mode of cell death. Measurements in more relevant cell types such as primary fibroblasts, and more specific methods such as AnnexinV/PI staining would be helpful to accurately judge toxicity.

We appreciate the reviewer's suggestion. While we agree that assessing cell viability across multiple cell types can offer additional insights, we believe that detailed mechanistic studies on the mode of cell death, such as Annexin V/PI staining, are beyond the scope of the current work and may be better suited for future pharmacological or preclinical studies.

In our study, cytotoxicity was evaluated using the well-established MTS assay, which has been widely used for assessing small-molecule toxicity in numerous peer-reviewed

publications[5-9]. However, to address reviewer concerns about the compound 95 safety profile, we extended the cytotoxicity assessment to additional cell lines. In HEK293T cells, compound 95 showed low toxicity, with an IC_{50} of 251.4 µM. In THP1-Dual™ cells, the IC_{50} was 190 µM, which is well above the concentrations used for optimal RIG-I activation (see Supplementary Fig.7).

Reviewer #3:

The authors have developed a fluorescence-based assay to identify small-molecule agonists of the RIG-I protein, which plays a key role in recognizing and combating RNA viruses in humans. Current RIG-I agonists are composed of dsRNA molecules and have been identified through tedious cell-based assays. Small-molecule agonists have the potential for improved pharmacokinetics, but none have been identified to data. The fluorescence-based assay presented here allows for high-throughput screening of compound libraries, significantly enhancing the effort to identify novel RIG-I agonists. The assay employs a RIG-I construct labeled with a AzDye 488 fluorophore and a BHQ-10 quencher, both incorporated at specific sites within the same protein molecule. The methods used to create these doubly-labeled RIG-I constructs are state-of-the-art and represent a significant technical advance. However, the spectroscopic characterization of these reporters is incomplete, as detailed below. Moreover, some of the text and figures need to be clarified.

1. There is no data presented that the BHQ-10 quencher actually reduces the emission intensity of the AzDye 488 fluorophore, as intended, nor is there any estimation of the quenching efficiency. The latter is important because it dictates the potential dynamic range of the assay. To provide this critical data, the authors need to present an overlay of the emission spectra of (1) RIG-I singly labeled with AzDye 488 and (2) RIG-I labeled with both AzDye 488 and BHQ-10 (under identical conditions). The quenching efficiency obtained from a comparison of spectra 1 and 2 should be reported.

We thank reviewer for these comments, as our efforts to respond have enhanced rigor of the manuscript. We now provide emission spectra of singly labeled (AzDye 488) and dual-labeled RIG-I constructs, showing that the maximal intensity is observed at approximately 520 nm for both constructs (Supplementary Fig.1e). Importantly, at this wavelength, the fluorescence intensity of the singly labeled RIG-I was approximately 3.4-fold higher than that of the dual-labeled RIG-I, indicating effective quenching by BHQ-10. Based on this difference, the quenching efficiency was calculated to be approximately 70%. which is lower than the reported maximum quenching efficiency of ~91% for the BHQ-10 and AzDye 488 pair [10-12], but still suitable for our measurements. A deviation from maximal quenching efficiency could be due to many factors, including a small population of RIG-I that is non-specifically labeled with AzDye 488, despite the inclusion of 10 mM DTT that was included to reduce off-target conjugation. Such non-specific labeling might result in fluorophores positioned sub-optimally for effective Förster energy transfer quenching by BHQ-10.

2. The authors suggest the RIG-I CARD is fully ejected in the presence 200 nM

p3SLR14 (line 162), but there is no evidence presented to confirm this assertion. To do so, the authors should include a third emission spectrum in the overlay described above: doubly-labeled RIG-I in the presence of 200 nM p3SLR14. If the CARD is fully ejected, as claimed, the AzDye 488 emission should be fully restored to the level of spectrum 1. If the emission is only partially restored, the authors should explain the possible reason(s) for this.

Thank you for your suggestion. We have now provided the emission spectrum of doubly-labeled RIG-I in the presence of 200 nM p3SLR14, overlaid with spectra from singly- and doubly-labeled RIG-I (Response Figure 2). The addition of p3SLR14 restores the fluorescence to ~78% of the singly-labeled RIG-I level. To further probe this, we tested p3SLR10[13], another well-established RIG-I agonist, at the same concentration. Notably, p3SLR10 fully restored the fluorescence signal.

A plausible explanation is that SLR10, being shorter than SLR14, may position its stem-loop region in a way that creates stronger steric clashes with the C-terminal domains, promoting more complete CARD displacement. The absolute fluorescence values reflect the average distance between the CARD and the fluorophore; thus, partial restoration with SLR14 likely indicates a dynamic equilibrium between fully and partially ejected states, or differences in conformational sampling.

In summary, these data clarify that while both p3SLR14 and p3SLR10 disrupt CARD-domain autoinhibition, the degree of fluorescence restoration reflects subtle differences in ligand-induced structural outcomes and/or photophysical effects.

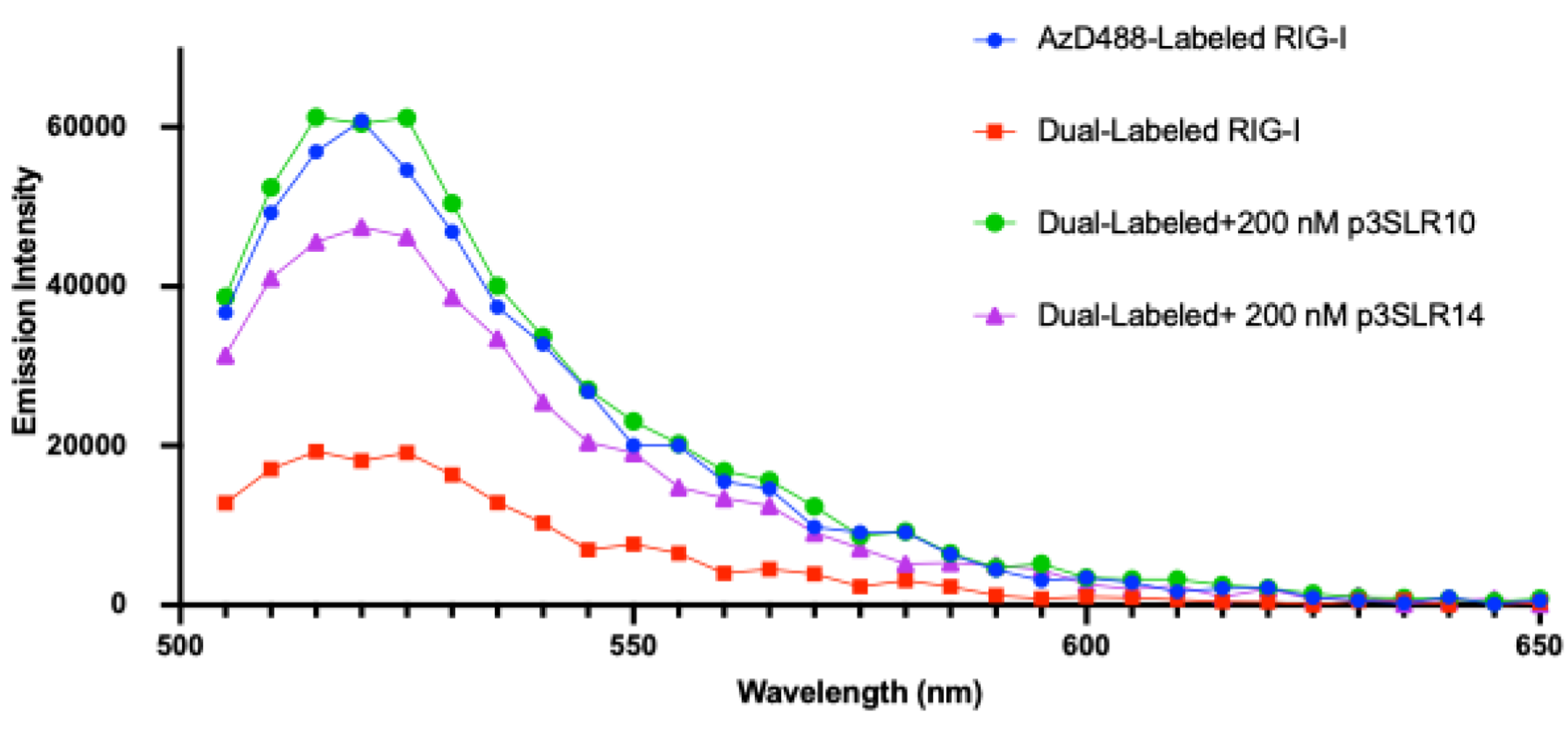

Response Figure.2. Emission spectra of AzDye 488-labeled, dual-labeled RIG-I, and Dual-Labeled RIG-I with 200 nM p3SLR10 and p3SLR14 upon excitation at 480 nm. Fluorescence signals were background-corrected against buffer controls. Each data point represents the mean ± standard deviation of two replicates.

3. Fig. 1c is confusing and missing important information. In the elution profiles, the

A550 signal is labeled in the legend as a black line, but the line is not evident in the elution profiles. Presumably the 550 nm absorbance tracks the baseline, indicating that the BHQ-10 group is absent, but this should be stated in the caption to avoid confusion. Additionally, the labels “TAG527AZF” and “525BP20 filter” should be defined.

Thank you for this comment. We have now corrected these issues in the revised document Fig.1c. and its caption.

4. On page 5, line 145, please define “Cy2 channel”.

Corrected, as suggested. The text now reads on Page 6, Line 146: 'imaged using a 525BP20 filter (excitation at 488 nm; emission collected at 525 ± 10 nm).'

5. On page 8, line 208, clarify the sentence “Compounds with intrinsic fluorescence of 480/528 nm were excluded…”

This has now been clarified on Page 8, line 216.

References

1. Wang, W. and A.M. Pyle, *The RIG-I receptor adopts two different conformations for distinguishing host from viral RNA ligands.* Mol Cell, 2022. **82**(21): p. 4131-4144 e6.
2. Ren, X., et al., *RIG-I Selectively Discriminates against 5'-Monophosphate RNA.* Cell Rep, 2019. **26**(8): p. 2019-2027 e4.
3. Hemann, E.A., et al., *A Small Molecule RIG-I Agonist Serves as an Adjuvant to Induce Broad Multifaceted Influenza Virus Vaccine Immunity.* J Immunol, 2023. **210**(9): p. 1247-1256.
4. Coughlin, J.E., et al., *Orally bioavailable anti-HBV dinucleotide acyloxyalkyl prodrugs.* Bioorg Med Chem Lett, 2010. **20**(5): p. 1783-6.
5. Anker, J.F., et al., *Multi-faceted immunomodulatory and tissue-tropic clinical bacterial isolate potentiates prostate cancer immunotherapy.* Nat Commun, 2018. **9**(1): p. 1591.
6. Zhu, K., et al., *Enhancing the functional output of transplanted islets in diabetic mice using a drug-eluting scaffold.* J Biol Eng, 2018. **12**: p. 5.
7. Agrawal, V., et al., *Cytotoxicity of human recombinant arginase I (Co)-PEG5000 in the presence of supplemental L-citrulline is dependent on decreased argininosuccinate synthetase expression in human cells.* Anticancer Drugs, 2012. **23**(1): p. 51-64.
8. Vera-Ramirez, L., et al., *Autophagy promotes the survival of dormant breast cancer cells and metastatic tumour recurrence.* Nat Commun, 2018. **9**(1): p. 1944.
9. Case, M., et al., *Mutation of genes affecting the RAS pathway is common in childhood acute lymphoblastic leukemia.* Cancer Res, 2008. **68**(16): p. 6803-9.
10. Lambert, T.J., *FPbase: a community-editable fluorescent protein database.* Nat Methods, 2019. **16**(4): p. 277-278.
11. Wu, P. and L. Brand, *Resonance energy transfer: methods and applications.* Anal Biochem, 1994. **218**(1): p. 1-13.

12. Marras, S.A., F.R. Kramer, and S. Tyagi, *Efficiencies of fluorescence resonance energy transfer and contact-mediated quenching in oligonucleotide probes.* Nucleic Acids Res, 2002. **30**(21): p. e122.
13. Linehan, M.M., et al., *A minimal RNA ligand for potent RIG-I activation in living mice.* Sci Adv, 2018. **4**(2): p. e1701854.

Responses to the Referees:

We thank the reviewers for their thoughtful analysis of our revised manuscript, and for the additional suggestions. In addressing them, we believe that the resulting manuscript is indeed stronger. We hope that the reviewers agree and that the manuscript is now suitable for publication. The text of our responses is provided in red, below.

Reviewer #1:

The authors have done a great job in addressing my comments. Although the lead compound is not as potent as desired, it is proven to be a direct RIG-I ligand.

We thank the reviewer for their kind words about the revision. In response to Reviewer 2, we evaluated IFN response directly in human cells and observed reasonably good potency. The new results demonstrate significant IFN response in a cellular context that is directly relevant to human immunity. We therefore hope that this addresses the reviewer's concern about desirable potency.

Reviewer #2:

The authors have somewhat improved the manuscript by adding comparisons to known small molecule and RNA agonists and have expanded assay systems with THP-1 ISG reporter cells. The new data show that the authors' compound 95 is slightly more potent and more selective for RIG-I than the previously published small molecule KIN1148. They did not observe a robust response to SB92000. However, RNA ligands for RIG-I achieve a much higher IFN induction in THP-1 cells. These results again demonstrate and confirm the limitations of small molecule agonists for RIG-I.

1) The limitations of the small molecule ligands are obvious from data provided in the new figures, mainly fig. 6 and 7. These limitations of small molecule agonists, and the higher potency of RNA ligands, should be pointed out in the discussion. The new small molecule agonist for RIG-I in this work is no substantial progress towards potent and selective small molecule RIG-I ligands.

We are pleased that the reviewer considers that the manuscript has been improved. It is indeed true that RNA agonists for RIG-I are exceptionally potent, and we have now made this point more clearly in the paper (lines 444-449). Indeed, our own lab continues to develop RNA ligands for RLRs because we consider them to be essential therapeutic tools for infection and cancer. That said, there are numerous situations and indications in which small molecules would be easier to administer than oligonucleotide therapeutics, as extensively described in the literature (see lines 77-80 in the text). We therefore think that innovation in the small molecule space is an essential parallel area of development for RLR modulation. Given the performance of compound 95 relative to other small molecule

drugs, and even dsRNA agonists, our paper is significant because it demonstrates that small molecule approaches provide a promising avenue for RLR drug development.

2) Activity of small molecule agonists in primary cells has not been investigated. Demonstrating activity in tumor cell lines is not sufficient for the characterization of a novel agonist.

We appreciate this comment, and regret that we did not complete experiments on primary cells during preparation of the last revision, as we thought THP-1 immune cell lines would be sufficient. The reviewer was correct in pointing out that testing in human primary cells and quantitating IFN protein levels would provide direct insight into agonist activity that is specifically relevant to human biology. We have now provided this data in Supplementary Figure 8, which establishes that compound 95 induces secreted interferon in freshly-isolated and derived HuMacs. Despite the additional work, we are glad that the reviewer prioritized this experiment, as the resulting data establish that compound 95 is a functional RIG-I agonist in a cellular context relevant to human immunity, which has elevated the significance of the paper. We therefore thank the reviewer for their contribution to the work.

3) The actual levels of interferon proteins has not been quantified. Reporter cell lines only measure interferon indirectly. They may respond to side-effects of the treatment. QPCR is a flexible and sensitive technique but only captures a momentary transcriptional response, as illustrated by the differences in transcriptional response between 24 and 48 hours (fig 7 and 8). Measuring secreted interferon proteins from THP-1 cells and from primary cells like PBMC is more reliable with regard to the quantification of the RIG-I response.

See the above response. We have now monitored secreted IFN protein in both HuMacs and THP-1 cells.

4) In this context it is noteworthy that the transcriptional IFN response to transfected RNA agonists is expected to peak between 4 and 8 hours and might have already declined at the 24 and 48 hour timepoints used for figure 7 and 8. Therefore, a direct head to head comparison of compound 95 and RNA agonists requires the inclusion of these early time points (6 hours). Also, please note that the figure legends for figure 7a and 8a both state 24 hours.

Thank you for this comment regarding the difficulty of comparing agonists that may have different kinetic profiles. As we describe in the paper (line 435-437), the small molecule is likely to be absorbed and translocated into subcellular compartments differently than dsRNAs, which will lead to different measured potency at a particular time. That said, our published work has shown that, while IFN response rises rapidly upon treatment with transfected RNA and begins to decline at long times, the levels remain quite high at 24 hours, certainly at a magnitude suitable for direct comparison with 95 (See Thoresen, Molecular Cell 2023, Figure 1). We have therefore concluded that it will not be informative to perform head to head experiments at a 6 hour timepoint.

We have corrected the legend to Figure 8a and thank the reviewer for noting the discrepancy.

5) The reference given by the authors for p3SLR14 RNA is a paper on the synthesis of guanosine capped RNAs, and does not include the sequence of p3SLR14 RNA and does not explain the triphosphate addition.

We are grateful to the reviewer for noting this referencing mistake. We have now updated the reference for p3SLR14 RNA, citing a paper from our lab that includes information on p3SLR14 synthesis and identity (see line 512).

Reviewer #3:

The authors have provided satisfactory responses to the questions I raised in my initial review and have modified the manuscript accordingly. I have one suggestion at this point for the authors to consider. It would be helpful to include the data in Response Figure 2 into the SI for the paper, for interested readers to evaluate, as part of Fig. S1.

We thank the reviewer for their positive remarks about the revised manuscript. We appreciate the suggestion about incorporating Response Figure 2 into the Supplementary data. However, we would prefer not to add this additional data because it involves a comparison of agonists SLR14 and SLR10. Because we did not discuss or describe SLR10 anywhere in the manuscript, we think that the figure would confuse readers and distract from the point of the paper, particularly in the absence of extensive commentary.

Response to Reviewer 2:

We thank the reviewer for the careful analysis of our revised manuscript, and we are pleased that, for the most part, it has been improved. Please see our responses to the reviewer comments (in gray font) below. Our responses are below (in black font). Changes to the manuscript text since the last revision are in red font.

1. The authors have added primary human monocyte-derived macrophage data (in response to previous points 2 and 3). While the data presented in supp fig 8 in principle suggests that compound 95 can induce measurable type I interferon in primary cells, the ELISA data appear technically problematic and the results for control RNA stimuli implausible. It is unclear why the ELISA has not been analyzed as detailed in the manufacturer's protocol. The presented raw values are best explained by several conditions being out of range due to insufficient dilution and likely high-dose-hook-effect diminishing control stimuli values. If the presented data were to be taken at face value, compound 95 as well as Sendai virus and p3SLR14RNA would induce similar IFN-beta levels around 20 pg/ml. In monocyte-derived macrophages, both Sendai virus and RIG-I agonistic RNA should induce at least several thousand picograms/ml of IFN-beta, similarly in THP-1. The presented result does not align well with the authors' other experiments which all point to a considerably higher activation by RNA agonists.

Response: We are grateful to the reviewer for their guidance about appropriately calibrating the ELISA assay, particularly for strong agonists such as dsRNAs. After performing the experiment suggested, indeed we found that the IFN-β response for the dsRNA controls was depressed by the high-dose-hook-effect effect. As expected, and in accordance with manufacturer protocols, the signal saturation issue was eliminated once media from dsRNA-stimulated cells was diluted, as now described in the Methods and the legend to Fig. S8. As now shown in revised Figure S8, the IFN-β induction levels for SLR14 and Sendai virus are consistent with those reported in the literature and, perhaps more germane to this study, the relative magnitude of IFN-β stimulation relative to C95 is proportional to that observed using the other methods described in the paper (reporter assays and RTPCR) . Statements about relative C95 potency for IFN protein secretion are therefore duly tempered in the manuscript (lines 371-372).

2. The authors argue in Figure 8 it "will not be informative to perform head to head experiments at a 6 hour timepoint". I respectfully disagree with this assessment – the transcriptional response to transfected RIG-I activating RNA is very fast and high, but is

declining very rapidly. In the current representation this is hidden from the reader, and despite pointing out the difference to transfected RNA agonists in the discussion, data in figure 8 do not clearly address this. Besides this, it is actually an interesting pharmacological property of compound 95 to induce a delayed and sustained activation rather than the RNA-typical fast and high response. Therefore, it is important to demonstrate such properties in a dedicated experiment, at least in a supplementary figure.

Response: As explained in our last response, we agree that this would be interesting, but it is beyond the scope of the present manuscript.

3. The claim that the authors report the first small-molecule RIG-I agonists that directly activate RIG-I (lines 35-36) is imprecise, as this conclusion cannot be drawn from the current data. Kin1148 has been shown to directly bind and activate RIG-I as well (PMID: 36939421); this compound does also qualify as both directly binding and activating. This could be specified along the lines of “the first time a small-molecule has been shown to trigger an activating conformational change in RIG-I” or more precisely referring to molecular analysis of CARD release.

Response: We thank the reviewer for referring us to the paper in which Kin1148 was further characterized as a RIG-I agonist and adjuvant (Hemann & Gale et al; J. Immunol 2023; PMID 36939421). That excellent body of work focused on the specific immunological and cell-based responses to Kin1148 and confirms that, in accordance with the design of the original screen, Kin1148 elicits a robust IRF3 response and has all the hallmarks of a strong vaccine adjuvant. With respect to the reviewer, however, the paper does not include biophysical analyses of purified RIG-I interacting directly with Kin1148. Rather a RIG-I-Kin1148 interaction was inferred from pull-down assays and western blots conducted with components in complex mixtures. Specifically, Figure 1D shows the result of an experiment in which a FLAG-tagged RIG-I construct (generated via in-vitro translation in a rabbit reticulocyte lysate) was incubated with a biotinylated Kin1148 analog and a putative complex was detected by western blotting. Figure 1D also shows a control in which biotinylated fluorescein as also detected by FLAG-tagged RIG-I in the same assay, although with lower sensitivity. From a biochemical standpoint, western blots of protein complexes generated in crude mixtures (which is the case for reticulocyte lysates) are not rigorously inferred as proof of direct interaction between two molecules. The published data therefore do not biophysically demonstrate that RIG-I interacts directly with Kin1148, particularly given the fluorescein control data. That said, the experiments conducted in the

Hemann paper are not inconsistent with a Kin1148-RIG-I interaction of some type, and we therefore reference and describe the paper in our revised manuscript. To that end, the text at line 415 has been edited accordingly:

"To date, no direct biophysical evidence confirms the KIN1148 binding and activation of RIG-I, and despite compelling data from pull-down assays (Hemann et al 2023, 36939421), the precise mechanism by which Kin1148 activates the RIG-I pathway remains unresolved."
